# PredicTF: prediction of bacterial transcription factors in complex microbial communities using deep learning

**DOI:** 10.1186/s40793-021-00394-x

**Published:** 2022-02-08

**Authors:** Lummy Maria Oliveira Monteiro, João Pedro Saraiva, Rodolfo Brizola Toscan, Peter F. Stadler, Rafael Silva-Rocha, Ulisses Nunes da Rocha

**Affiliations:** 1 Helmholtz Center for Environmental Research (UFZ), Leipzig, Germany; 2 Bioinformatics Group, Institute of Computer Science, Universität Leipzig, Leipzig, Germany; 3 Ribeirão Preto Medical School (FMRP), University of São Paulo (USP), Ribeirão Prêto, Brazil

**Keywords:** Gene regulation, Transcription factors, Deep learning, Transcription factor database, Microbial communities

## Abstract

**Background:**

Transcription factors (TFs) are proteins controlling the flow of genetic information by regulating cellular gene expression. A better understanding of TFs in a bacterial community context may open novel revenues for exploring gene regulation in ecosystems where bacteria play a key role. Here we describe PredicTF, a platform supporting the prediction and classification of novel bacterial TF in single species and complex microbial communities. PredicTF is based on a deep learning algorithm.

**Results:**

To train PredicTF, we created a TF database (BacTFDB) by manually curating a total of 11,961 TF distributed in 99 TF families. Five model organisms were used to test the performance and the accuracy of PredicTF. PredicTF was able to identify 24–62% of the known TFs with an average precision of 88% in our five model organisms. We demonstrated PredicTF using pure cultures and a complex microbial community. In these demonstrations, we used (meta)genomes for TF prediction and (meta)transcriptomes for determining the expression of putative TFs.

**Conclusion:**

PredicTF demonstrated high accuracy in predicting transcription factors in model organisms. We prepared the pipeline to be easily implemented in studies profiling TFs using (meta)genomes and (meta)transcriptomes. PredicTF is an open-source software available at https://github.com/mdsufz/PredicTF.

**Supplementary Information:**

The online version contains supplementary material available at 10.1186/s40793-021-00394-x.

## Background

The functional potential of microbial communities can be determined by the genetic content of their constituent members. However, genetic content alone does not guarantee that a given function or enzymatic reaction would be performed [1]. Transcription Factor proteins (TFs) play a central and critical role in gene regulation in this scenario. These proteins are indirectly responsible for optimizing proteins and structural RNAs and the subsequent levels of metabolites and other properties, ensuring the survival and adaptation of organisms to the most diverse types of stress and environmental changes [2]. Environmental signals modulate the activity and expression levels of bacterial TFs (e.g., changes in the oxygen condition, temperature, pH or the lack of a specific substrate) [3]. Additionally, for many promoters, combinations of transcription factors work together to integrate different signals [2, 4]. TFs can also work with other DNA-binding proteins whose primary role is to sculpt the bacterial folded chromosome [2, 4]. Knowledge of the TFs profile expressed by an organism is the first step to better understand the regulatory network that controls protein expression in an organism or a community.

Since TFs may determine when and which genes are expressed [1–3], profiling TFs can help understand gene expression regulation and build regulatory networks in complex microbial communities. Further, defining which factors control gene expression may offer insights into the mechanisms controlling ecosystem processes and even interactions between species of a microbial community [5]. However, current TF databases are focused on single or small groups of genomes. Mostly, these databases are manually curated based on literature evidence and pairwise sequence comparison of genomes from model organisms. Examples of these databases include RegulonDB for *Escherichia coli* K-12 [6], DBTBS for *Bacillus subtilis* [7], FlyBase for *Drosophila* [8], and FTFD for fungal species [9]. DBD [10] is a database generated from the prediction of TFs from 150 sequenced genomes across the tree of life. Unfortunately, DBD has not been updated for more than nine years.

One of the primary goals in manipulating microbiomes for ecological and biotechnological applications is to control the outcome of their functions [11]. As TFs are key to potentially controlling which genes are expressed, one of the best ways to study and understand gene regulation in a microbiome may be to profile its TFs. To date, only a few tools such as P2TF [12] and DeepTFactor [13] have been developed to predict and classify bacterial TFs. P2TF [12] performs domain analysis of each protein sequence using RPS-BLAST. TF candidates are selected if they present an e-value below 0.01 and a minimum alignment coverage of 50% for each domain length. DeepTFactor [13] employs convolutional neural networks to extract features from an embedded matrix (generated from a protein sequence) and predict if a given protein sequence is a transcription factor or not. This tool showed higher specificity rates when compared to P2TF [13].

Deep learning approaches have been used to predict DNA sequence affinities [14] and identify TF-binding sites in humans [15]. Although deep learning has been used in gene regulation, it has rarely been used to predict bacterial TFs. Further, the need for a user-friendly tool for predicting TFs that could assist in gene regulation analysis motivated the development of PredicTF. Here, we constructed a robust database for bacterial transcriptional factors (BacTFDB) that was used to train our deep learning model. PredicTF was evaluated by assessing its ability to predict known and described TFs in model organisms accurately. PredicTF is a deep learning tool able to predict and annotate TFs in (meta)genomes from full protein-length sequences and can be found at https://github.com/mdsufz/PredicTF.

## Results and discussion

**Fig. 1:**
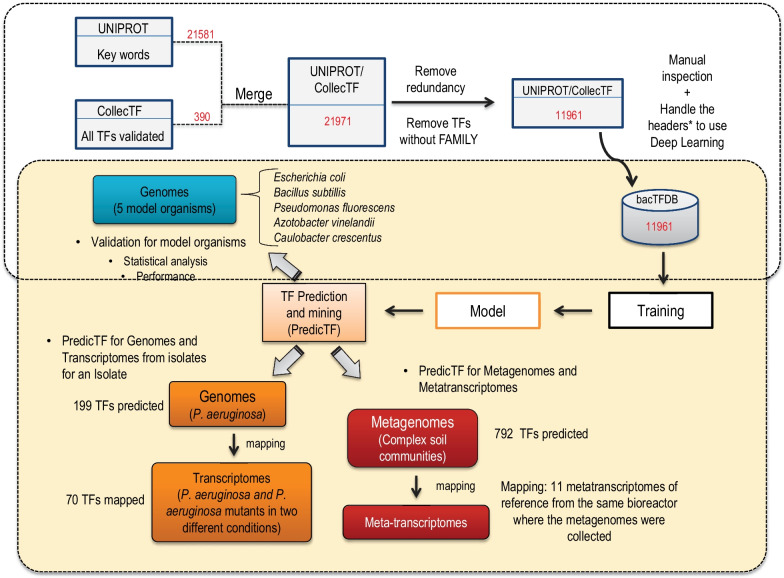
PredicTF workflow and testing. We collected publicly available data on TFs from two different databases: CollecTF and UniProtKB. After removing redundancies and filtering TFs well characterized, this data (BacTFDB) was used to train a deep learning model to predict new TFs and their families. Five model organisms (*Escherichia coli, Bacillus subtillis, Pseudomonas fluorescens, Azotobacter vinelandii and Caulobacter crescentus*) were used to test the accuracy of PredicTF. Later, we used the same approach to predict TFs from an isolate (*P. aeruginosa*) and mapped TFs predicted in transcriptomics data (*P. aeruginosa* and mutants in two experimental conditions). Finally, we used our tool to predict TF in complex communities (metagenome) and mapped these TFs in their respective meta-transcriptomes

PredictTF is a command-line software for the prediction of novel transcription factors from genomic and metagenomic data. We created a bacterial transcription factor database (BacTFDB) by merging and manually curating TFs present in CollectTF [16] and UniProtKB/Swiss-Prot, a manually annotated and reviewed section of the UniProt Knowledgebase (UniProtKB) [17]. CollectTF provides well-described and characterized in vivo validated TFs, while UniProtKB is a comprehensive resource for protein sequence and annotation data. We used BacTFDB to train a deep learning model to predict new TFs and their families in genomes and metagenomes. Five model organisms (*Escherichia coli, Bacillus subtillis, Pseudomonas fluorescens, Azotobacter vinelandii and Caulobacter crescentus*) were used to test the performance and accuracy of PredicTF. To this purpose, we removed the TFs for each of these model organism’s genus from our database to remove the bias of having sequences from closely related species during the PredicTF performance. Additionally, we removed from the database the *Enterobacteriaceae* family to evaluate the TF predictions in *E. coli*. We used the same approach to predict TFs from a clinical isolate (*P. aeruginosa* PAO1) and a metagenome sample isolated from an anaerobic ammonium oxidation community. We also determined if the predicted TFs were expressed in transcriptomes (isolate) and metatranscriptomes (microbial community), respectively (Fig. 1).

### Database

**Fig. 2:**
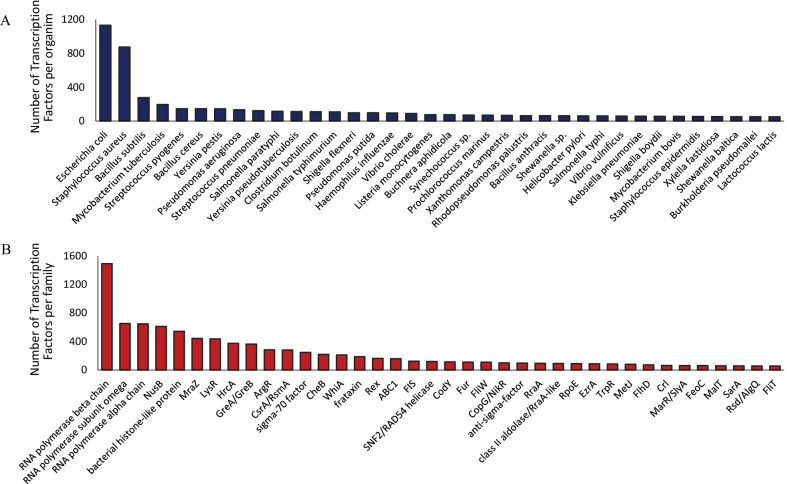
Database composition: Transcription Factor Database (BacTFDB) distribution. **A** Database distribution based on the TFs and **B** regulatory elements families and organisms species. These graphics show only families with up to 50 sequences and only organisms that contributed with more than 50 sequences

BacTFDB is a robust bacterial TF database containing 11,691 TFs amino acid sequences spanning 1049 TF families and 720 different bacterial species. Figure 2 shows the database distribution based on TF families and regulatory elements (Fig. 2a) and the distribution based on bacterial species (Fig. 2b). Although BacTFDB comprises a significant and diverse amount of TF sequences, Fig. 2 shows that many TFs families and organisms accumulate more than 50 sequences each. We will update BacTFDB annually by adding novel entries deposited in UniProtKB and CollecTF. BacTFDB was used in PredicTF’s deep learning model training, and this model was later used to predict new TFs and their families in genomes and metagenomes.

### Performance and accuracy

We evaluated the performance and accuracy of PredicTF through the prediction of TFs in five model organisms (*E. coli, B. subtillis, P. fluorescens, A. vinelandii and C. crescentus*). We trained a different PredicTF model for each model organism to predict TFs from full protein-length sequences (described in the “Materials and methods” session). In parallel, we performed full genome annotation of each model organism using Prokka [18] with default parameters (e.g., non-redundant database).

**Fig. 3:**
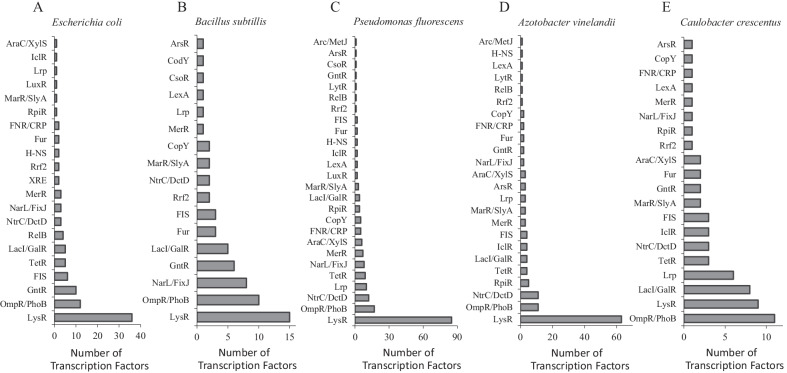
Prediction of TFs by PredicTF for genomes of model organisms. Prediction of TFs or 5 model organisms sorted by family. **A** *Escherichia coli* **B** *Bacillus subtillis* **C** *Caulobacter crescentus* **D** *Pseudomonas fluorescens* **E** *Azotobacter vinelandii*

Identification of TFs using PredicTF in the different model organisms ranged from 24 to 62% of the proteins described as TFs in model organisms’ genomes. The accuracy for experimentally validated TFs ranged from 87.76% and 98.69% (Table 1). Further, PredicTF was able to identify putative annotated TFs in the genomes of *E. coli* and *B. subtillis* with accuracies of 85.71%, and 100%, respectively (Table 1). No potentially novel TF was predicted in the genome of *C. crescentus*, *P. fluorescens* and *A. vinelandii* (Table 1). TFs predicted by PredicTF for each organism, sorted by TF family, are shown in Fig. 3. For all organisms tested, the most predicted TF family was LysR, which represents the most abundant type of transcriptional regulator in the prokaryotic kingdom, and OmpR/PhoB, a global transcriptional regulator implicated in the control of various cellular processes and functions in many Gram-negative bacteria. The degree of accuracy obtained by PredicTF suggests that the deep learning strategy used is promising for the prediction of TFs in genomic or metagenomic data of bacterial species. PredicTF performance and accuracy can be further improved by expanding the number and diversity of sequences present in BacTFDB. As BacTFDB will be updated yearly, we expect to improve TF identification with every update.

**Table 1:** PredicTF performance, accuracy for experimentally validated Transcription Factors (Accuracy EV), and accuracy for putative Transcription Factors (Accuracy PU) in genomes of model organisms. We removed the sequences from the genus and/or family of the different model organisms from our TF database (BacTFDB) before model training to reduce the chances of false positives (i.e., the presence of identical sequences in the training dataset)

Organism	Performance^a^ (%)	Accuracy^b^ EV^b^ (%)	Model^c^	Accuracy PU^d^
*E. coli k12*	33.33	94.44	PredicTF-no- *Escherichia*	85.71
*E. coli k12*	31.27	95.12	PredicTF-no- *Enterobacteriaceae*	85.71
*B. subtillis*	24.26	87.76	PredicTF-no- *Bacillus*	100
*C. crescentus*	34.36	96.00	PredicTF-no- *Caulobacter*	-^e^
*P. fluorescens*	46.44	98.69	PredicTF-no- *Pseudomonas*	-
*A.vinelandii*	62.28	98.43	PredicTF-no- *Azotobacter*	-

^a^Performance was calculated by the ratio of the total number of TFs predicted by PredicTF (*Predicted TFs*) to the total number of proteins annotated as TFs in NCBI (*Annotated TFs*) multiplied by 100

^b^Accuracy EV was determined by the ratio of the total number of TFs predicted by PredicTF in agreement with NCBI annotation (*TFs predicted correctly*) to the total number of TFs predicted by PredicTF (*TFs predicted*) multiplied by 100

^c^PrecicTF Model used for the prediction of TF to the specific organism

^d^Accuracy TU was determined by the total number of putative TFs predicted correctly divided by putative TFs predicted multiplied by 100; *Putative TFs predicted correctly is the total number of putative TFs predicted correctly by PredicTF in agreement with NCBI annotation*; and, *Putative TFs predicted* is the total number of putative TFs predicted by PredicTF

^e^Currently there are no putative annotated TFs described in the genome of *C. crescentus, P.* fluorescens and *A.vinelandii*

PredicTF showed an average precision, recall, and F1 scores of 0.88, 0.40, and 0.54, while Prokka showed an average of 0.93, 0.83, and 0.88, respectively (Additional file 4: Table S1). However, recall using Prokka is twice of that observed for PredicTF. A possible explanation for lower precision and recall rates using PredicTF is derived from the removal of species-specific TFs for each model organism (which was done to avoid overfitting).

Although PredicTF showed lower recall rates in the model organisms, it presented fewer false positives when compared to Prokka for four out of the five model organisms tested (Additional file 4: Table S1). PredicTF *C. vibrioides* (strain NA1000/CB15N) false positives were 3 times fold lower when compared to Prokka. *E. coli* (strain K-12 sub strain MG1655), *B. subtilis* (strain 168), and *P. fluorescens* (strain F113) PredicTF’s results were, respectively, 46.7%, 21.0%, and 9.1% fewer than those observed through Prokka. Prokka only showed fewer false positives than PredicTF for the isolate *A. vinelandii* (strain DJ / ATCC BAA-1303), respectively 46.5% (Additional file 4: Table S1). Further, extracting the predicted TFs from Prokka results requires manual labor. In contrast, predicted TFs using PredicTF are presented in a clear table (e.g., https://github.com/mdsufz/PredicTF/blob/master/Examples/predictf_output.txt). Nevertheless, we expect to improve PredicTF’s performance by increasing sequences in future updates to BacTFDB.

### Mining and predicting TFs in genomes and transcriptomes from a bacterial isolate using PredicTF

PredicTF was used to predict TFs on the genome of *P. aeruginosa* PAO1 and these TFs were mapped in transcriptomes from the same isolate [19]. PredicTF predicted a total of 199 TFs in the *P. aeruginosa* PAO1 genome shown in Additional file 1: Fig. S1 A by a family’s distribution graphic. These 199 TFs were mapped in the transcriptomic data of a reference of *P. aeruginosa* PAO1. Initially, the mapping was done in the transcriptome of *P. aeruginosa* PAO1 cultured in LB media. Using this strategy, we were able to map 69 of the 199 predicted TFs to the transcriptomes under the experimental conditions carried out by Hwang and Yoon, 2019 (Additional file 1: Fig. S1 B) [19]. Next, the mappings were done for another three clinical mutants of *P. aeruginosa* PAO1 (Y82, Y71, Y89) cultured in LB media (absence of an antibiotic cocktail) (Additional file 2: Fig. S2 A, C and F). The TFs family’s distribution for each *P. aeruginosa* PAO1 mutant cultured in the presence of antibiotic cocktail is shown in the supplementary data (Additional file 2: Fig. S2 B, D, and F). These results demonstrate the potential of PredicTF in mapping regulatory elements in bacterial genomes and the use of this tool to map and compare TFs profiles under different environmental conditions. Nevertheless, further studies are necessary to validate if the predicted TFs are indeed transcription factors.

### Mining and predicting TFs in a metagenome and metatranscriptome using PredicTF

**Fig. 4:**
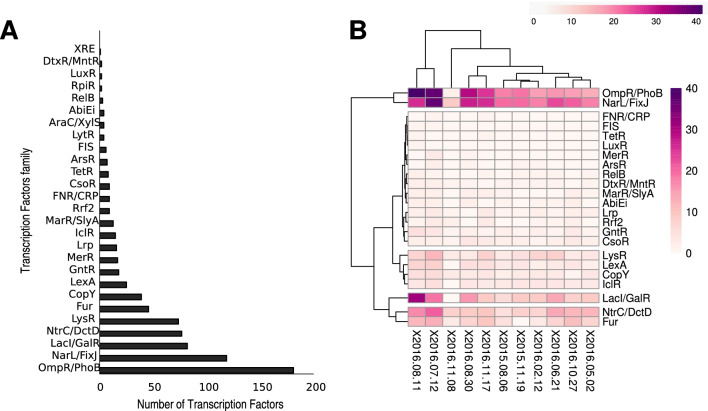
Recovery of novel Transcription Factors in one metagenome and eleven metatranscriptomes. **a** PredicTF predicted 792 TFs were predicted in one anaerobic ammonium oxidizing microbial communities from anammox membrane bioreactor (LAC_MetaG_1) and were grouped by family. **b** Using 792 TFs predicted in one metagenome, we mapped these TFs for 11 metatranscriptomes of reference from the same bioreactor where the metagenome was recovered

PredicTF was used to profile TFs in one metagenome recovered from an anaerobic ammonium oxidation community [20] followed by mapping the predicted TFs in metatranscriptomes recovered from the same community (metatranscriptomes accession numbers can be found in Additional file 5: Table S2). We predicted 792 TFs (Fig. 4a) in LAC_MetaG_1, an anaerobic ammonium oxidizing microbial community from an anammox membrane bioreactor [20]. These 792 TFs belong to 27 different TF families (Fig. 4a). They are related to the regulation of functions such as the oxygen limitation response and late symbiotic functions (NarL/FixJ), phosphate regulon response (OmpR/PhoB), transcriptional activator for nitrogen-regulated promoters (NtrC/DctD), and ferric uptake regulation (Fur). To determine how a traditional annotation pipeline identifies potential TFs, we used Prokka [18]. This tool was able to identify 1815 ORFs (Additional file 6: Table S3). PredicTF can be used with no previous knowledge regarding transcription factors, it is fast, and it requires low memory when compared to Blast-based annotation. It indicates only results of TFs with a specific TF family annotation. On the other hand, to identify TFs using Prokka, one would need specialized training to mine the general annotation. Therefore, scientists with a general microbiology background may take a long time to undergo this task. Further, Prokka does not indicate the TF families of the putative annotated TFs. Time is also a drawback of using Prokka to mine TFs. We calculated we needed over 400 h to mine one single metagenomic library; in comparison, PredicTF needed 2 h for the same task. Also, users can quickly generate new models for predicting TFs once BacTFDB is updated.

Next, the 792 TFs were mapped in 11 metatranscriptomes collected on different dates from the same bioreactor where the metagenome was recovered Additional file 7: Table S4, Fig. 4b). Clustering analysis demonstrated the presence of five different groups of TFs families based on the number of transcription factor families expressed in each library (Fig. 4b). It is interesting to note that the two most abundant clusters in the heatmap are directly related to the oxygen limitation caused by the anaerobic ammonium oxidizing cultivation. In a bioreactor where oxygen is limited, an increase in nitrogen and phosphate is expected. The presence of N and P diverts the metabolism of the microbial community towards the production of regulators (TFs) that help maintain community stability. Clustering analysis can be helpful to demonstrate the similarity between metatranscriptomic libraries based on the occurrence of TFs. This strategy can be useful to compare the profiles of TFs expressed in different environmental situations (comparing libraries with different metadata), creating patterns of TFs expression. The exploration of TF profiling in microbial communities (metagenomes or metatranscriptomes) will allow the exploration of regulation within complex microbial communities. Further, the recovery of metagenome-assembled genomes is becoming standard in metagenomics studies [21–23]. The use of PredicTF together with the recovery of metagenome-assembled genomes will allow the exploration of species-specific molecular mechanisms involved in the regulation of different ecosystem processes.

### Conclusions

A better understanding of TFs in a bacterial community context opens avenues for the exploration of gene regulation in ecosystems where bacteria play a key role. Our deep learning strategy was based on a novel and robust TF bacterial database (BacTFDB) with over 11 thousand TFs and their respective families. BacTFDB is a unique resource for studies involving TFs, and it provided the data to train a model within PredicTF capable of predicting novel TFs from genomes and metagenomes. PredicTF is the first pipeline designed to predict and annotate TFs in complex microbial communities. The prediction of TFs can provide information for those aiming to study and understand bacterial communities within a context of gene regulation. We also demonstrated that PredicTF could be used to predict novel TFs in metagenomes and metatrascriptomes, creating the potential profile for regulatory elements in complex microbial communities.

PredicTF is a user-friendly and open-source pipeline able to predict and annotate TFs in genomes and metagenomes and can be found at https://github.com/mdsufz/PredicTF.

## Materials and methods

### BacTFDB: Bacterial transcription factor data base

We collected data from two publicly available databases to create a novel Bacterial Transcription Factor Database (BacTFDB). Initially, we collected data from CollecTF [16], a well-described and characterized database. Since CollecTF does not provide an application programming interface (API) for bulk download, we developed a Python code (version 2.7) using the Beautiful Soup 4.4.0 library to recover the data from CollecTF. With this strategy, we listed 390 TF experimentally validated amino acid sequences distributed over 44 TF families. The script can be found at https://github.com/mdsufz/PredicTF.

Additionally, we retrieved TF amino acid sequences from UniProtKB using UniProt’s API. We downloaded sequences of interest from UniProtKB that belonged to the bacteria taxonomy by adding a filter with the keywords (Transcription factor, transcriptional factor, regulator, transcriptional repressor, transcriptional activator, transcriptional regulator). We accessed the UniProtKB API on 8^th^ September-2019, and a total of 21.581 TF amino acid sequences, with applied filters, were collected. We merged the data collected from CollecTF and UniProtKB databases resulting in 21.971 TFs. Next, we removed redundant TF entries and TF sequences lacking a TF family since PredicTF was also designed to assign a TF family. Finally, we performed a manual inspection to remove case sensitivity and the characters associated with the database header. The first version of BacTFDB contains a total of 11.691 unique TF sequences. A summary of the information contained in BacTFDB can be found in the supplementary data (Additional file 3: Fig. S3). To evaluate PredicTF in model organisms, we created five subsets of BacTFDB. The description of these subsets can be found in the supplementary data (Additional file 8: Table S5).

### Mapping transcription factors using PredictTF

We used the deep learning approach developed by Gustavo and collaborators [24]. Supervised machine learning methods are usually performed in three stages: characterization, training, and evaluation. Briefly, the characterization relied on the concept of dissimilarity-based classification [25], where sequences are represented and featured by their sequence similarity to known genes. This deep learning approach also considers both the similarity distribution of sequences in the database used for training and the best hit. We maintained the same parameters as in the study by Gustavo and collaborators [24]. Briefly, The protein sequences used in the deep neural network are aligned to the sequences in BacTFDB using DIAMOND with very relaxed constraints (10,000 maximum number of hits, a minimum of 20% identity score, and a maximum e-value of 1e−10). Next, the normalized bit score is used to represent the sequence similarity as a distance to known transcription factors, which, in turn, is used to populate a feature matrix. The latter is used to calculate the identity distance distribution of target sequences to all sequences in BacTFDB, which is propagated throughout the hidden layers of the neural network. We set the number of dense hidden layers used to propagate the bit score distribution to dense and abstract features to four.

Lastly, we calculated the probability of the target sequence to each TF family. For more details on the deep learning model generation, please see Gustavo et al. [24]. The complete set of transcription factors in BacTFDB used to train and test the deep learning models, and the model itself (defined as PredicTF) are publicly available in (https://github.com/mdsufz/PredicTF). Next, PredicTF was used to predict TFs from full protein-length sequences in non-model organisms and one metagenome. After prediction, the data was mapped to transcriptomes and metatranscriptomes from samples where we determined the genetic potential.

To ensure that the training and independent test sets do not have identical or near-identical examples, we trained five different models—one for each model organism (Additional file 5: Table S2). For each model, the TFs affiliated with the respective model organism genus and/or family were removed before training to avoid overfitting due to the homology of the sequences. PredicTF-no-*Escherichia* and PredicTF-no-*Enterobacteriaceae* were trained to predict TFs in *E. coli,* PredicTF-no-*Bacillus* was trained to predict TFs in *B. subtilis,* PredicTF-no-*Caulobacter* was trained to predict TFs in *C. crescentus*, PredicTF-no-*Pseudomonas* was trained to predict TFs in *P. fluorescens* and PredicTF-no-*Azotobacterial* was trained to predict TFs in *A. vinelandii*.

### Performance and accuracy calculation

We evaluated PredicTF by calculating accuracy and performance. Performance was calculated by quantifying the number of TFs that PredicTF was able to predict divided by the number of TFs already described and annotated in our model organisms (Additional file 9: Equation 1). We determined accuracy by calculating the number of TFs correctly predicted divided by the total number of TFs predicted by PredicTF in each model organism. We divided accuracy into two categories. In the first accuracy category, we determined accuracy against experimentally validated TFs (Additional file 9: Equation 2). In the second accuracy category, we determined accuracy against TFs without experimental validation (Additional file 9: Equation 3), putative TFs. The performance, accuracy, and accuracy for putative TFs were calculated as the ratio of predicted to annotated TFs.Precision, recall, and F1-scores equations are shown in Additional file 9: Equations 4, 5, and 6.

### Prediction of transcription factors in model organisms

We selected bacterial species that have been widely studied as model organisms. Some bacterial species became model organisms for TF studies because they are easy to maintain and grow in a laboratory setting and manipulate in pure culture experiments. Five complete genomes from model organisms (*E. coli, B. subtillis, P. fluorescens, A. vinelandii and C. crescentus*) were downloaded directly from NCBI. The strains details and accession number (RefSeq) for all selected organisms are listed in the supplementary data (Additional file 5: Table S2). By evaluating PredicTF using model organisms (Additional file 7: Table S4) we extrapolated the performance of our deep learning model. Since known TFs for each organism were removed from each training dataset, we eliminate the possibility of mapping TFs already known and annotated for each of the different species (i.e., avoiding overfitting). Performance for putative TFs of PredicTF for the selected five model organisms was calculated using the equations described in the Additional file 9: Equations).

To have a baseline comparison with a traditional annotation pipeline, we used Prokka [18] to annotate each model organism using default parameters.

### Prediction of transcription factors in a clinical isolate

We demonstrated the use of PredicTF in a previously sequenced *P. aeruginosa* (PAO1) genome, a clinical isolate publicly available in NCBI (accession number NC_002516.2). *P. aeruginosa* PAO1 was selected because its genome has been sequenced and because of the availability of transcriptomes from three clinical mutants of PAO1 (Y71, Y82, and Y89) grown in the presence and absence of an antibiotic cocktail. The transcriptomes of *P. aeruginosa* PAO1 mutants Y71, Y82, and Y89 are available in NCBI (Bioproject identifier PRJNA479711) [18]. These clinical *P. aeruginosa* PAO1 mutants were isolated from the sputa of three different pneumonia patients. Transcriptomes of *P. aeruginosa* PAO1 wild type and its mutants cultured in two different conditions (LB medium and LB medium in the presence of antibiotic cocktail) have been previously described [19]. We used this data to determine the TF profile in these *P. aeruginosa* PAO1 mutants grown in two different conditions.

PredicTF was first used to predict TFs in the *P. aeruginosa* PAO1 genome. Next, the predicted TFs were mapped to the transcriptomes of the *P. aeruginosa* PAO1 mutants Y71, Y82 and Y89 (see later). Further description of the mapping of the transcriptomes to the genomes is available at https://github.com/mdsufz/PredicTF. We trained the PredicTF model used in this step with the full database (BacTFDB). All accession numbers used in this work are listed in the supplementary data (Additional file 5: Table S2).

### Prediction of transcription factors in complex microbial communities

To test PredicTF in a complex microbial community, we used an anaerobic ammonium oxidizing (anammox) microbial community from an anammox membrane bioreactor metagenome (LAC_MetaG_1) (data publicly available at NCBI bioproject via accession number PRJNA511011) [20]. According to the developer’s instructions, we removed short and low-quality reads using Trim Galore—v0.0.4 dev [26]. Over 50 million reads survived this step and were assembled using the de novo assembler metaSPADES—v3.12.0 [27]. The assembly was translated from nucleotide to amino acid sequences, considering all possible translation frames, using emboss transeq [28]. The translated assembly was then used as input to predict transcription factors using PredicTF. Then, we extracted the region from each predicted TF. These putative TFs were later used when mapping TFs to metatranscriptomes.

We checked if the putative TFs predicted in the metagenomes were transcribed by checking if the metatranscriptomic libraries were mapping to those regions. The metatranscriptomic and metagenomic libraries used in this step belonged to the same bioreactor. These metatranscriptomes are publicly available at the European Nucleotide Archive under the accession numbers SRR7091385, SRR7523233, SRR7523244, SRR7523245, SRR7091400, SRR7091401, SRR7091381, SRR7091402, SRR7091406, SRR7523243, SRR7523246. These 11 metatranscriptomes were used to demonstrate the effectiveness of the pipeline and to indicate the potential of PredicTF to profile transcription factors in complex microbial communities. All accession numbers used in this work are listed in the supplementary data (Additional file 5: Table S2).

To have a baseline comparison with a traditional annotation pipeline, we used Prokka [18] to annotate the same anammox membrane bioreactor metagenome (LAC_MetaG_1). We mined the annotation by hand with the knowledge of scientists specialized in Transcription Factors. We did not determine the families as this work would need to be done for every single hit individually using the output of Prokka.

### Mapping transcription factors to transcriptomes and metatranscriptomes

Each transcriptomic and metatranscriptomic library was quality controlled by removing short and low-quality reads using Trim Galore—v0.0.4 dev [26]. After quality checking, the seven transcriptomic libraries for the *P. aeruginosa* PAO1 wild type and mutants showed at least 26 million paired-end reads. The 11 metatranscriptomic libraries yielded over 50 million reads per library after quality check. After, the remaining transcriptomic and metatranscriptomic reads were mapped to their respective assembled genome or metagenome using Bowtie2—v2.3.0 [29]. The number of reads mapped, and the regions covered was extracted using SAMTools—v1.9 [30] and python 2.7. The regions of the genome or metagenome assembly covered by transcriptomic or metatranscriptomic reads were then crossed-referenced with the regions of their respective assembly, which PredicTF assigned as putative TFs creating a TF profile for each transcript and metatranscriptome. A detailed description of how we mapped the RNA-seq data to their respective genome or metagenome assembly can be found at the PredicTF github (https://github.com/mdsufz/PredicTF).

## Supplementary Information

**Additional file 1: Fig. S1**. Transcription factor (TF) families predicted for *Pseudomonas aeruginosa* PAO1 genome (accession number NC_002516.2) (18) using PredicTF and their mapping to *P. aeruginosa* PAO1 growing in LB medium. A) A total of 199 TFs distributed in 25 TF families were predicted in the *P. aeruginosa* PAO1 genome. B) These 199 TFs were mapped in the transcriptomic data of a reference of *P. aeruginosa* PAO1 (Bioproject identifier PRJNA479711) (18). Initially, we did the mapping in the transcriptome of *P. aeruginosa* PAO1 cultured in LB media. Using this strategy, we were able to map 69 of the 199 predicted TFs to the transcriptome (PDF 73 kb)

**Additional file 2: Fig. S2**. Transcription Factor (TF) family profiles in three *Pseudomonas aeruginosa* PAO1 mutants. After the prediction of Transcription Factors (TFs) using *P. aeruginosa* PAO1 genome, we mapped transcriptomes from three *P. aeruginosa* PAO1 mutants (Y82, Y71, Y89) cultured in LB media (A, C, and F). After, we did the mapping for each *P. aeruginosa* PAO1 mutant cultured in the presence of an antibiotic cocktail (B, D, and E). *P. aeruginosa* PAO1 mutant Y82 (A, B); P. aeruginosa PAO1 mutant Y71 (C, D); *P. aeruginosa* PAO1 mutant Y89 (E, F)

**Additional file 3: Fig. S3**. Bacterial Transcription Factor Data Base (BacTFDB) was created from two publicly available databases. We collected 390 TFs from CollecTF and 21.581 from UniProtKB (accessed 8-Sep-2019), accumulating 21.581 Transcription Factor (TF) amino acid sequences. We merged the data from CollecTF and UniProtKB databases resulting in a total of 21.971 TFs amino acids. We removed redundant TF entries, and since PredicTF was also designed to assign TF family, TF sequences lacking a TF family were removed. Finally, we performed a manual inspection to remove misleading spelling, case sensitivity, and characters associated with the database header. The final database (BacTFDB) contains a total of 11.691 TF unique sequences

**Additional file 4: Table S1**. Confusion matrices, precision, recall, and F1-scores for prediction of transcription factors in each model organism using PredicTF and Prokka.

**Additional file 5: Table S2**. The accession numbers for the five model organisms, *Pseudomonas aeruginosa* PAO1 genome and transcriptomes, and Complex Microbial Communities used to validate and test PredicTF.

**Additional file 6: Table S3**. Transcription factors from the metagenome of an anaerobic ammonium oxidizing microbial community from an anammox membrane bioreactor (LAC_MetaG_1) that we mined and hand-curated from a general annotation generated using Prokka (18).

**Additional file 7: Table S4**. The number of Transcription Factors (TFs) per TF family mapped to each of the 11 metatranscriptomes of reference from the same bioreactor where the metagenome (accession number PRJNA511011, NCBI) used to predict the putative TFs was collected. Their European Nucleotide Archive accession numbers represent the different metatranscriptomes.

**Additional file 8: Table S5**. Description of the bacterial transcriptional factors database (BacTFDB) subsets used to train models to predict Transcription Factors in model organisms.

**Additional file 9: Equations**. The different equations we used to calculate PredicTF’s accuracy and performance.

## Data Availability

Project name: PredicTF. Project home page: https://github.com/mdsufz/PredicTF. Operating system: Linux64. Programming languages: Python 2.7. Other requirements: DIAMOND [31]; Nolearn Lasagne deep learning library [32]; Sklearn machine learning routines (https://scikit-learn.org/stable/) [33]; Theano (http://deeplearning.net/software/theano/) [34]. Trim Galore—v0.0.4 dev (https://www.bioinformatics.babraham.ac.uk/projects/trim_galore/) [26]. MetaSPADES—v3.12.0 (https://github.com/ablab/spades#meta) [27]. Emboss transeq (http://www.bioinformatics.nl/cgi-bin/emboss/transeq) [28]. Bowtie2—v2.3.0 (https://sourceforge.net/projects/bowtie-bio/) [29]. SAMTools—v1.9 (http://github.com/samtools/) [30]. Genomes of the model organisms used in the Tool Validation step are available at the National Center for Biotechnology Information (https://www.ncbi.nlm.nih.gov/) under the accession numbers NC_000913.3, NC_000964.3, NC_011916.1, NC_021149.1, and NC_016830. The datasets supporting the Prediction of Transcription Factors in a clinical isolate of this article are available at National Center for Biotechnology Information (https://www.ncbi.nlm.nih.gov/) under the accession number NC_002516.2 (genome) and study accession PRJNA479711 (transcriptomes). The datasets used for the Prediction of Transcription Factors in Complex Microbial Communities of this study are available at National Center for Biotechnology Information (https://www.ncbi.nlm.nih.gov/) under the study accession PRJNA511011. The respective data sets of metatranscriptomes used are available at National Center for Biotechnology Information (https://www.ncbi.nlm.nih.gov/) under the SRA numbers SRR7091385, SRR7523233, SRR7523244, SRR7523245, SRR7091400, SRR7091401, SRR7091381, SRR7091402, SRR7091406, SRR7523243, SRR7523246 and the Joint Genome Institute (https://jgi.doe.gov/) under the Gold Analysis Project identifiers Gp0267156, Gp0267150, Gp0267154, Gp0267155, Gp0267157, Gp0267158, Gp026715, Gp0267159, Gp0267152, Gp0267153, Gp0267160. All analysis, results and scripts used to generate figures are available at https://github.com/mdsufz/PredicTF.

## Abbreviations

TFs: Transcription factors
BacTFDB: Bacterial Transcription Factor Data Base
TFBSs: Transcription factor binding sites
anammox: Anaerobic ammonium oxidizing

## References

[1] Liu J, Meng Z, Liu X, Zhang X-H (2019). Microbial assembly, interaction, functioning, activity and diversification: a review derived from community compositional data. Mar Life Sci Technol.

[2] Browning DF, Butala M, Busby SJW (2019). Bacterial transcription factors: regulation by Pick “N” Mix. J Mol Biol.

[3] Browning DF, Busby SJW (2016). Local and global regulation of transcription initiation in bacteria. Nat Rev Microbiol.

[4] Browning DF, Grainger DC, Busby SJ (2010). Effects of nucleoid-associated proteins on bacterial chromosome structure and gene expression. Curr Opin Microbiol.

[5] Morales SE, Holben WE (2011). Linking bacterial identities and ecosystem processes: can ‘omic’ analyses be more than the sum of their parts?. FEMS Microbiol Ecol.

[6] Gama-Castro S, Salgado H, Santos-Zavaleta A, Ledezma-Tejeida D, Muñiz-Rascado L, García-Sotelo JS, Alquicira-Hernández K, Martínez-Flores I, Pannier L, Castro-Mondragón JA, Medina-Rivera A, Solano-Lira H, Bonavides-Martínez C, Pérez-Rueda E, Alquicira-Hernández S, Porrón-Sotelo L, López-Fuentes A, Hernández-Koutoucheva A, Del Moral-Chávez V, Rinaldi F, Collado-Vides J (2016). RegulonDB version 9.0: high-level integration of gene regulation, coexpression, motif clustering and beyond. Nucleic Acids Res.

[7] Sierro N, Makita Y, de Hoon M, Nakai K (2008). DBTBS: a database of transcriptional regulation in *Bacillus subtilis* containing upstream intergenic conservation information. Nucleic Acids Res.

[8] The FlyBase consortium (1997). FlyBase: a Drosophila database. Nucleic Acids Res.

[9] Park J, Park J, Jang S, Kim S, Kong S, Choi J, Ahn K, Kim J, Lee S, Kim S, Park B, Jung K, Kim S, Kang S, Lee Y-H (2008). FTFD: an informatics pipeline supporting phylogenomic analysis of fungal transcription factors. Bioinformatics.

[10] Kummerfeld SK, Teichmann SA (2006). DBD: a transcription factor prediction database. Nucleic Acids Res.

[11] Widder S, Allen RJ, Pfeiffer T, Curtis TP, Wiuf C, Sloan WT, Cordero OX, Brown SP, Momeni B, Shou W, Kettle H, Flint HJ, Haas AF, Laroche B, Kreft J-U, Rainey PB, Freilich S, Schuster S, Milferstedt K, van der Meer JR, Groβkopf T, Huisman J, Free A, Picioreanu C, Quince C, Klapper I, Labarthe S, Smets BF, Wang H, Fellows INI, Soyer OS (2016). Challenges in microbial ecology: building predictive understanding of community function and dynamics. ISME J.

[12] Ortet P, De Luca G, Whitworth DE, Barakat M (2012). P2TF: a comprehensive resource for analysis of prokaryotic transcription factors. BMC Genom.

[13] Kim GB, Gao Y, Palsson BO, Lee SY (2021). DeepTFactor: a deep learning-based tool for the prediction of transcription factors. Proc Natl Acad Sci USA.

[14] Alipanahi B, Delong A, Weirauch MT, Frey BJ (2015). Predicting the sequence specificities of DNA- and RNA-binding proteins by deep learning. Nat Biotechnol.

[15] Pan X, Shen H-B (2017). RNA-protein binding motifs mining with a new hybrid deep learning based cross-domain knowledge integration approach. BMC Bioinform.

[16] Kiliç S, White ER, Sagitova DM, Cornish JP, Erill I (2014). CollecTF: a database of experimentally validated transcription factor-binding sites in Bacteria. Nucleic Acids Res.

[17] The UniProt Consortium (2021). UniProt: the universal protein knowledgebase in 2021. Nucleic Acids Res.

[18] Seemann T (2014). Prokka: rapid prokaryotic genome annotation. Bioinformatics.

[19] Hwang W, Yoon SS (2019). Virulence characteristics and an action mode of antibiotic resistance in multidrug-resistant *Pseudomonas aeruginosa*. Sci Rep.

[20] Keren R, Lawrence JE, Zhuang W, Jenkins D, Banfield JF, Alvarez-Cohen L, Zhou L, Yu K (2020). Increased replication of dissimilatory nitrate-reducing bacteria leads to decreased anammox bioreactor performance. Microbiome.

[21] Parks DH, Rinke C, Chuvochina M, Chaumeil P-A, Woodcroft BJ, Evans PN, Hugenholtz P, Tyson GW (2017). Recovery of nearly 8,000 metagenome-assembled genomes substantially expands the tree of life. Nat Microbiol.

[22] Pasolli E, Asnicar F, Manara S, Zolfo M, Karcher N, Armanini F, Beghini F, Manghi P, Tett A, Ghensi P, Collado MC, Rice BL, DuLong C, Morgan XC, Golden CD, Quince C, Huttenhower C, Segata N (2019). Extensive unexplored human microbiome diversity revealed by over 150,000 genomes from metagenomes spanning age, geography, and lifestyle. Cell.

[23] Tully BJ, Graham ED, Heidelberg JF (2018). The reconstruction of 2,631 draft metagenome-assembled genomes from the global oceans. Sci Data.

[24] Arango-Argoty G, Garner E, Pruden A, Heath LS, Vikesland P, Zhang L (2018). DeepARG: a deep learning approach for predicting antibiotic resistance genes from metagenomic data. Microbiome.

[25] Sørensen L, Loog M, Lo P, Ashraf H, Dirksen A, Duin RPW, de Bruijne M, Jiang T, Navab N, Pluim JPW, Viergever MA (2010). Image dissimilarity-based quantification of lung disease from CT. Medical image computing and computer-assisted intervention—MICCAI 2010.

[26] Babraham Bioinformatics - Trim Galore!.

[27] Nurk S, Meleshko D, Korobeynikov A, Pevzner PA (2017). metaSPAdes: a new versatile metagenomic assembler. Genome Res.

[28] Madeira F, Park YM, Lee J, Buso N, Gur T, Madhusoodanan N, Basutkar P, Tivey ARN, Potter SC, Finn RD, Lopez R (2019). The EMBL-EBI search and sequence analysis tools APIs in 2019. Nucleic Acids Res.

[29] Langmead B, Salzberg SL (2012). Fast gapped-read alignment with Bowtie 2. Nat Methods.

[30] Li H, Handsaker B, Wysoker A, Fennell T, Ruan J, Homer N, Marth G, Abecasis G, Durbin R (2009). The sequence alignment/map format and SAMtools. Bioinformatics.

[31] Buchfink B, Xie C, Huson DH (2015). Fast and sensitive protein alignment using DIAMOND. Nat Methods.

[32] van Merriënboer B, Bahdanau D, Dumoulin V, Serdyuk D, Warde-Farley D, Chorowski J, Bengio Y (2015) Blocks and fuel: frameworks for deep learning. arXiv:150600619 [cs, stat].

[33] Pedregosa F, Varoquaux G, Gramfort A, Michel V, Thirion B, Grisel O, Blondel M, Prettenhofer P, Weiss R, Dubourg V, Vanderplas J, Passos A, Cournapeau D. Scikit-learn: machine learning in Python. Machine learning in Python 6.

[34] The Theano Development Team, Al-Rfou R, Alain G, Almahairi A, Angermueller C, Bahdanau D, Ballas N, Bastien F, Bayer J, Belikov A, Belopolsky A, Bengio Y, Bergeron A, Bergstra J, Bisson V, Snyder JB, Bouchard N, Boulanger-Lewandowski N, Bouthillier X, de Brébisson A, Breuleux O, Carrier P-L, Cho K, Chorowski J, Christiano P, Cooijmans T, Côté M-A, Côté M, Courville A, Dauphin YN, Delalleau O, Demouth J, Desjardins G, Dieleman S, Dinh L, Ducoffe M, Dumoulin V, Kahou SE, Erhan D, Fan Z, Firat O, Germain M, Glorot X, Goodfellow I, Graham M, Gulcehre C, Hamel P, Harlouchet I, Heng J-P, Hidasi B, Honari S, Jain A, Jean S, Jia K, Korobov M, Kulkarni V, Lamb A, Lamblin P, Larsen E, Laurent C, Lee S, Lefrancois S, Lemieux S, Léonard N, Lin Z, Livezey JA, Lorenz C, Lowin J, Ma Q, Manzagol P-A, Mastropietro O, McGibbon RT, Memisevic R, van Merriënboer B, Michalski V, Mirza M, Orlandi A, Pal C, Pascanu R, Pezeshki M, Raffel C, Renshaw D, Rocklin M, Romero A, Roth M, Sadowski P, Salvatier J, Savard F, Schlüter J, Schulman J, Schwartz G, Serban IV, Serdyuk D, Shabanian S, Simon É, Spieckermann S, Subramanyam SR, Sygnowski J, Tanguay J, van Tulder G, Turian J, Urban S, Vincent P, Visin F, de Vries H, Warde-Farley D, Webb DJ, Willson M, Xu K, Xue L, Yao L, Zhang S, Zhang Y (2016( Theano: a Python framework for fast computation of mathematical expressions. arXiv:160502688 [cs].

