# Supplementary material for: PredicTF: prediction of bacterial transcription factors in complex microbial communities using deep learning
Source: Environ Microbiome. 2022 Feb 8;17:7. doi: 10.1186/s40793-021-00394-x (PMC8822659; doi:10.1186/s40793-021-00394-x)
Supplement: Supplementary file 1 — Additional file 1: Fig. S1. Transcription factor (TF) families predicted for Pseudomonas aeruginosa PAO1 genome (accession number NC_002516.2) (18) using PredicTF and their mapping to P. aeruginosa PAO1 growing in LB medium. A) A total of 199 TFs distributed in 25 TF families were predicted in the P. aeruginosa PAO1 genome. B) These 199 TFs were mapped in the transcriptomic data of a reference of P. aeruginosa PAO1 (Bioproject identifier PRJNA479711) (18). Initially, we did the mapping in the transcriptome of P. aeruginosa PAO1 cultured in LB media. Using this strategy, we were able to map 69 of the 199 predicted TFs to the transcriptome (PDF 73 kb) [file 40793_2021_394_MOESM1_ESM.pdf]

PredicTF: prediction of bacterial transcription factors in complex microbial communities using deep learning

Lummy Maria Oliveira Monteiro[1,2,3], Joao Saraiva[1], Rodolfo Brizola Toscan[1], Peter F Stadler[2], Rafael Silva-Rocha[3], Ulisses Nunes da Rocha[1]*

[1] Helmholtz Center for Environmental Research (UFZ), Leipzig, Germany
[2] Universität Leipzig (UL), Leipzig, Germany
[3] Ribeirão Preto Medical School (FMRP), University of São Paulo (USP), Ribeirão Preto, Brazil

*Correspondence: Ulisses Nunes da Rocha, ulisses.rocha@ufz.de

Figure S1

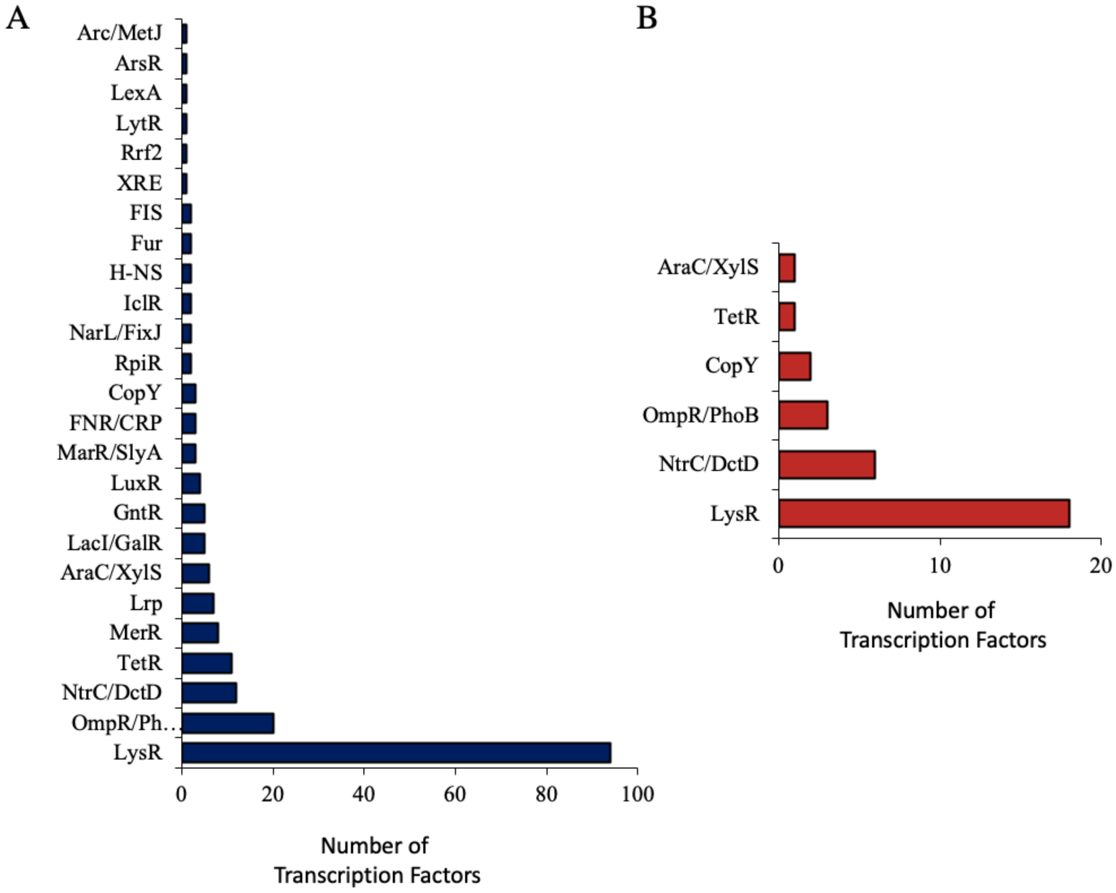

Legend. Transcription factor (TF) families predicted for *Pseudomonas aeruginosa* PAO1 genome (accession number NC_002516.2) (18) using PredicTF and their mapping to *P. aeruginosa* PAO1 growing in LB medium. A) A total of 199 TFs distributed in 25 TF families were predicted in the *P. aeruginosa* PAO1 genome. B) These 199 TFs were mapped in the transcriptomic data of a reference of *P. aeruginosa* PAO1 (Bioproject identifier PRJNA479711) (18). Initially, we did the mapping in the transcriptome of *P. aeruginosa* PAO1 cultured in LB media. Using this strategy, we were able to map 69 of the 199 predicted TFs to the transcriptome.
