# Supplementary material for: PredicTF: prediction of bacterial transcription factors in complex microbial communities using deep learning
Source: Environ Microbiome. 2022 Feb 8;17:7. doi: 10.1186/s40793-021-00394-x (PMC8822659; doi:10.1186/s40793-021-00394-x)
Supplement: Supplementary file 2 — Additional file 2: Fig. S2. Transcription Factor (TF) family profiles in three Pseudomonas aeruginosa PAO1 mutants. After the prediction of Transcription Factors (TFs) using P. aeruginosa PAO1 genome, we mapped transcriptomes from three P. aeruginosa PAO1 mutants (Y82, Y71, Y89) cultured in LB media (A, C, and F). After, we did the mapping for each P. aeruginosa PAO1 mutant cultured in the presence of an antibiotic cocktail (B, D, and E). P. aeruginosa PAO1 mutant Y82 (A, B); P. aeruginosa PAO1 mutant Y71 (C, D); P. aeruginosa PAO1 mutant Y89 (E, F) [file 40793_2021_394_MOESM2_ESM.pdf]

# PredicTF: prediction of bacterial transcription factors in complex microbial communities using deep learning

Lummy Maria Oliveira Monteiro[1,2,3], Joao Saraiva[1], Rodolfo Brizola Toscan[1], Peter F Stadler[2], Rafael Silva-Rocha[3], Ulisses Nunes da Rocha[1]*

[1] Helmholtz Center for Environmental Research (UFZ), Leipzig, Germany

[2] Universität Leipzig (UL), Leipzig, Germany

[3] Ribeirão Preto Medical School (FMRP), University of São Paulo (USP), Ribeirão Preto, Brazil

*Correspondence: Ulisses Nunes da Rocha, ulisses.rocha@ufz.de

**Figure S2**

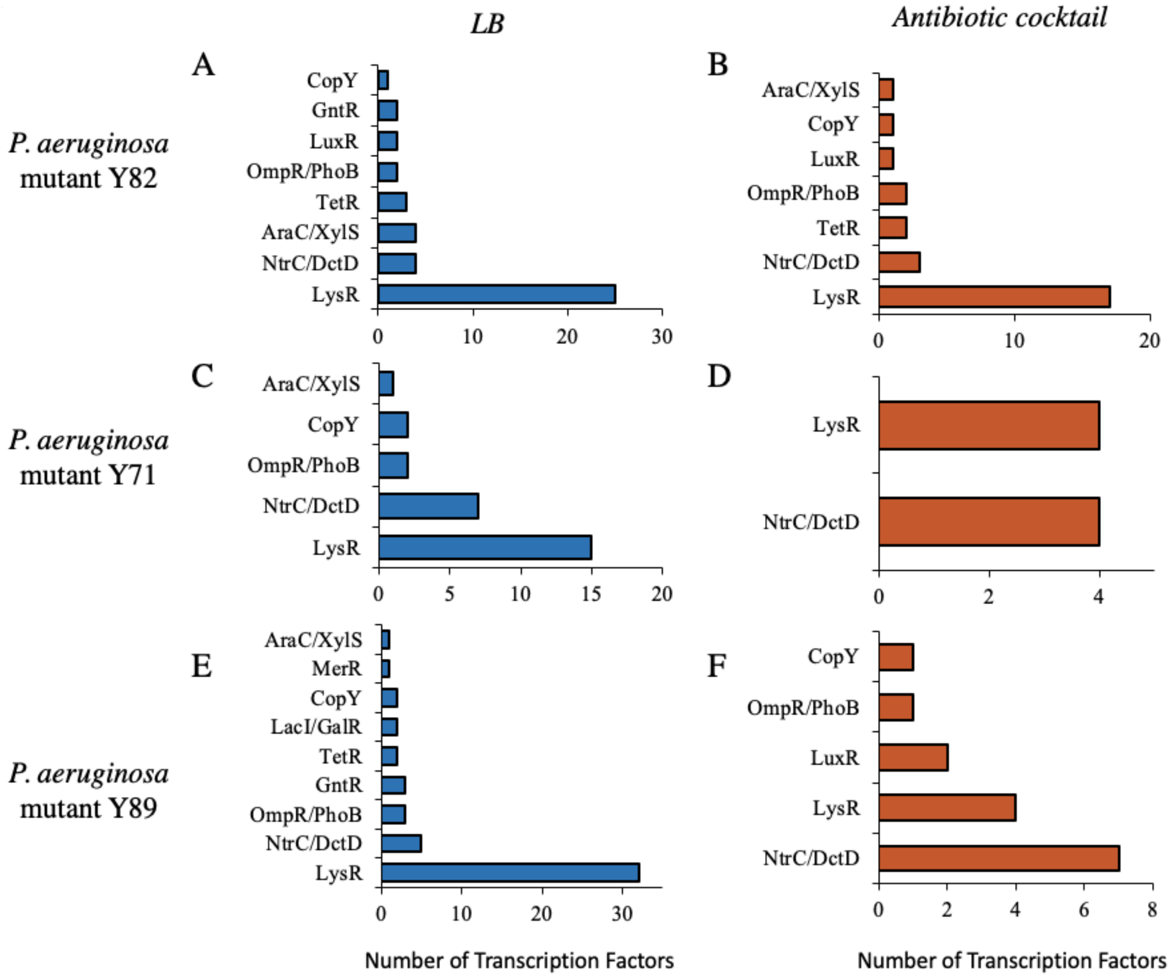


**Legend.** Transcription Factor (TF) family profiles in three *Pseudomonas aeruginosa* PAO1 mutants. After the prediction of Transcription Factors (TFs) using *P. aeruginosa* PAO1 genome, we mapped transcriptomes from three *P. aeruginosa* PAO1 mutants (Y82, Y71, Y89) cultured in LB media (A, C, and F). After, we did the mapping for each *P. aeruginosa* PAO1 mutant cultured in the presence of an antibiotic cocktail (B, D, and E). *P. aeruginosa* PAO1 mutant Y82 (A, B); P. aeruginosa PAO1 mutant Y71 (C, D); *P. aeruginosa* PAO1 mutant Y89 (E, F).
