# Supplementary material for: PredicTF: prediction of bacterial transcription factors in complex microbial communities using deep learning
Source: Environ Microbiome. 2022 Feb 8;17:7. doi: 10.1186/s40793-021-00394-x (PMC8822659; doi:10.1186/s40793-021-00394-x)
Supplement: Supplementary file 3 — Additional file 3: Fig. S3. Bacterial Transcription Factor Data Base (BacTFDB) was created from two publicly available databases. We collected 390 TFs from CollecTF and 21.581 from UniProtKB (accessed 8-Sep-2019), accumulating 21.581 Transcription Factor (TF) amino acid sequences. We merged the data from CollecTF and UniProtKB databases resulting in a total of 21.971 TFs amino acids. We removed redundant TF entries, and since PredicTF was also designed to assign TF family, TF sequences lacking a TF family were removed. Finally, we performed a manual inspection to remove misleading spelling, case sensitivity, and characters associated with the database header. The final database (BacTFDB) contains a total of 11.691 TF unique sequences [file 40793_2021_394_MOESM3_ESM.pdf]

PredicTF: prediction of bacterial transcription factors in complex microbial communities using deep learning

Lummy Maria Oliveira Monteiro[1,2,3], Joao Saraiva[1], Rodolfo Brizola Toscan[1], Peter F Stadler[2], Rafael Silva-Rocha[3], Ulisses Nunes da Rocha[1]*

[1] Helmholtz Center for Environmental Research (UFZ), Leipzig, Germany
[2] Universität Leipzig (UL), Leipzig, Germany
[3] Ribeirão Preto Medical School (FMRP), University of São Paulo (USP), Ribeirão Preto, Brazil

---

*Correspondence: Ulisses Nunes da Rocha, ulisses.rocha@ufz.de

Figure S3

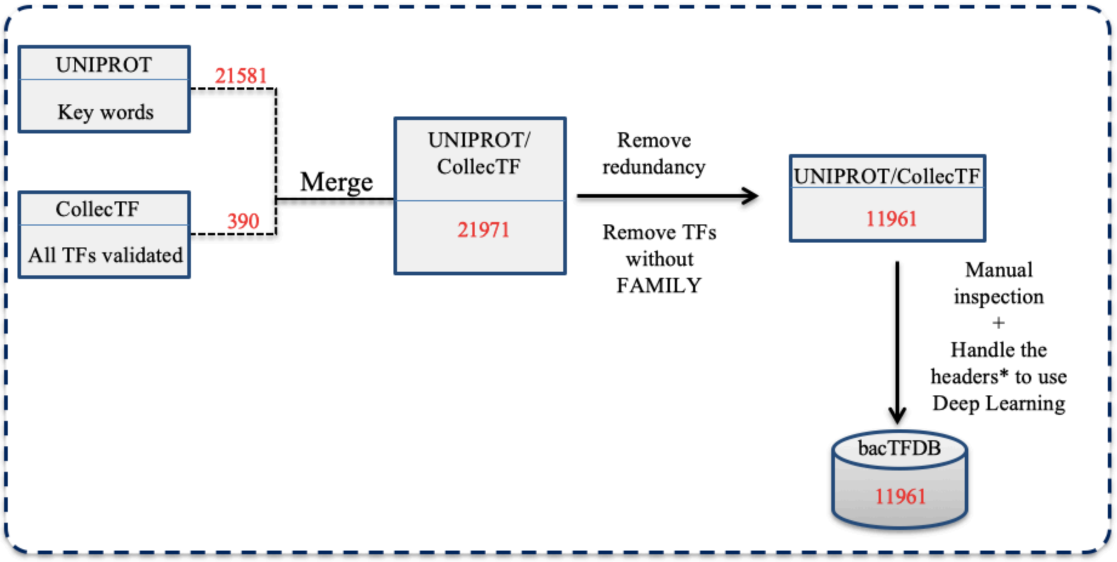

Legend. Bacterial Transcription Factor Data Base (BacTFDB) was created from two publicly available databases. We collected 390 TFs from CollecTF and 21.581 from UniProtKB (accessed 8-Sep-2019), accumulating 21.581 Transcription Factor (TF) amino acid sequences. We merged the data from CollecTF and UniProtKB databases resulting in a total of 21.971 TFs amino acids. We removed redundant TF entries, and since PredicTF was also designed to assign TF family, TF sequences lacking a TF family were removed. Finally, we performed a manual inspection to remove misleading spelling, case sensitivity, and characters associated with the database header. The final database (BacTFDB) contains a total of 11.691 TF unique sequences.
