# Supplementary material for: PredicTF: prediction of bacterial transcription factors in complex microbial communities using deep learning
Source: Environ Microbiome. 2022 Feb 8;17:7. doi: 10.1186/s40793-021-00394-x (PMC8822659; doi:10.1186/s40793-021-00394-x)
Supplement: Supplementary file 4 — Additional file 4: Table S1. Confusion matrices, precision, recall, and F1-scores for prediction of transcription factors in each model organism using PredicTF and Prokka. [file 40793_2021_394_MOESM4_ESM.pdf]

PredicTF: prediction of bacterial transcription factors in complex microbial communities using deep learning

Lummy Maria Oliveira Monteiro[1,2,3], Joao Saraiva[1], Rodolfo Brizola Toscan[1], Peter F Stadler[2], Rafael Silva-Rocha[3], Ulisses Nunes da Rocha[1]*

[1] Helmholtz Center for Environmental Research (UFZ), Leipzig, Germany

[2] Universität Leipzig (UL), Leipzig, Germany

[3] Ribeirão Preto Medical School (FMRP), University of São Paulo (USP), Ribeirão Preto, Brazil

---

*Correspondence: Ulisses Nunes da Rocha, ulisses.rocha@ufz.de

Table S1. Confusion matrices, precision, recall, and F1-scores for prediction of transcription factors in each model organism using PredicTF and Prokka.

PredicTF

Azotobacter	Predicted TF	Predicted non-TF
Verified TF	142	86
Verified non-TF	15	4805

**Precision**	0.9044586
**Recall**	0.62280702
**F1**	0.73766234

Prokka

Azotobacter	Predicted TF	Predicted non-TF
Verified TF	211	17
Verified non-TF	7	4830

**Precision**	0.96788991
**Recall**	0.9254386
**F1**	0.94618834

PredicTF

Bacillus	Predicted TF	Predicted non-TF
Verified TF	57	178
Verified non-TF	19	8220

**Precision**	0.75
**Recall**	0.24255319
**F1**	0.36655949

Prokka

Bacillus	Predicted TF	Predicted non-TF
Verified TF	203	32
Verified non-TF	23	8216

**Precision**	0.89823009
**Recall**	0.86382979
**F1**	0.88069414

PredicTF

Caulobacter	Predicted TF	Predicted non-TF
Verified TF	56	107
Verified non-TF	3	3720

**Precision**	0.94915254
**Recall**	0.34355828
**F1**	0.5045045

Prokka

Caulobacter	Predicted TF	Predicted non-TF
Verified TF	105	58
Verified non-TF	9	3714

**Precision**	0.92105263
**Recall**	0.64417178
**F1**	0.75812274

PredicTF

Pseudomonas	Predicted TF	Predicted non-TF
Verified TF	176	203
Verified non-TF	11	5472

**Precision**	0.94117647
**Recall**	0.46437995
**F1**	0.62190813

Prokka

Pseudomonas	Predicted TF	Predicted non-TF
Verified TF	336	43
Verified non-TF	12	5471

**Precision**	0.96551724
**Recall**	0.88654354
**F1**	0.92434663

PredicTF

Escherichia	Predicted TF	Predicted non-TF
Verified TF	97	194
Verified non-TF	15	3973

**Precision**	0.86607143
**Recall**	0.33333333
**F1**	0.48138958

Prokka

Escherichia	Predicted TF	Predicted non-TF
Verified TF	244	47
Verified non-TF	22	3966

**Precision**	0.91729323
**Recall**	0.83848797
**F1**	0.87612208
