# Supplementary material for: PredicTF: prediction of bacterial transcription factors in complex microbial communities using deep learning
Source: Environ Microbiome. 2022 Feb 8;17:7. doi: 10.1186/s40793-021-00394-x (PMC8822659; doi:10.1186/s40793-021-00394-x)
Supplement: Supplementary file 5 — Additional file 5: Table S2. The accession numbers for the five model organisms, Pseudomonas aeruginosa PAO1 genome and transcriptomes, and Complex Microbial Communities used to validate and test PredicTF. [file 40793_2021_394_MOESM5_ESM.pdf]

PredicTF: prediction of bacterial transcription factors in complex microbial communities using deep learning

Lummy Maria Oliveira Monteiro[1,2,3], Joao Saraiva[1], Rodolfo Brizola Toscan[1], Peter F Stadler[2], Rafael Silva-Rocha[3], Ulisses Nunes da Rocha[1]*

[1] Helmholtz Center for Environmental Research (UFZ), Leipzig, Germany
[2] Universität Leipzig (UL), Leipzig, Germany
[3] Ribeirão Preto Medical School (FMRP), University of São Paulo (USP), Ribeirão Preto, Brazil

*Correspondence: Ulisses Nunes da Rocha, ulisses.rocha@ufz.de

Table S2 The accession numbers for the five model organisms, *Pseudomonas aeruginosa* PAO1 genome and transcriptomes, and Complex Microbial Communities used to validate and test PredicTF

Model Organisms

Description	RefSeq	Location
***Escherichia coli* (strain K-12 sub strain MG1655)**	NC_000913.3	(NCBI) Genbank
***Bacillus subtilis* (strain 168)**	NC_000964.3	(NCBI) Genbank
***Caulobacter vibrioides* (strain NA1000 / CB15N) (*Caulobacter crescentus*)**	NC_011916.1	(NCBI) Genbank
***Azotobacter vinelandii* (strain DJ / ATCC BAA-1303)**	NC_021149.1	(NCBI) Genbank
***Pseudomonas fluorescens* (strain F113)**	NC_016830.1	(NCBI) Genbank

Clinical Isolate

Description	RefSeq	Location
***Pseudomonas aeruginosa* (PAO1) genome**	NC_002516.2	(NCBI) Genbank
**Transcriptomes from three clinical isolates (Y71, Y82, and Y89)**	PRJNA479711	(NCBI) Genbank

Complex Microbial Communities

Description	RefSeq	Identification	Date of Collection	Location
**Metagenomic dataset**	PRJNA511011	LAC_MetaG_1		Genbank (NCBI)
**Meta-transcriptomes of anaerobic ammonium oxidizing microbial communities from anammox membrane bioreactor (MBR)**	SRR7091385	LAC_MetaT_1	2015-08-06	European Nucleotide Archive (ENA)
	SRR7523233	LAC_MetaT_2	2015-11-19	
	SRR7523244	LAC_MetaT_3	2016-02-12	
	SRR7523245	LAC_MetaT_4	2016-05-02	
	SRR7091400	LAC_MetaT_5	2016-06-21	
	SRR7091401	LAC_MetaT_6	2016-07-12	
	SRR7091381	LAC_MetaT_7	2016-08-11	
	SRR7091402	LAC_MetaT_8	2016-08-30	
	SRR7091406	LAC_MetaT_9	2016-10-27	
	SRR7523243	LAC_MetaT_10	2016-11-08	
	SRR7523246	LAC_MetaT_11	2016-11-17	
