# Supplementary material for: PredicTF: prediction of bacterial transcription factors in complex microbial communities using deep learning
Source: Environ Microbiome. 2022 Feb 8;17:7. doi: 10.1186/s40793-021-00394-x (PMC8822659; doi:10.1186/s40793-021-00394-x)
Supplement: Supplementary file 6 — Additional file 6: Table S3. Transcription factors from the metagenome of an anaerobic ammonium oxidizing microbial community from an anammox membrane bioreactor (LAC_MetaG_1) that we mined and hand-curated from a general annotation generated using Prokka (18). [file 40793_2021_394_MOESM6_ESM.pdf]

PredicTF: prediction of bacterial transcription factors in complex microbial communities using deep learning

Lummy Maria Oliveira Monteiro[1,2,3], Joao Saraiva[1], Rodolfo Brizola Toscan[1], Peter F Stadler[2], Rafael Silva-Rocha[3], Ulisses Nunes da Rocha[1]*

[1] Helmholtz Center for Environmental Research (UFZ), Leipzig, Germany
[2] Universität Leipzig (UL), Leipzig, Germany
[3] Ribeirão Preto Medical School (FMRP), University of São Paulo (USP), Ribeirão Preto, Brazil

*Correspondence: Ulisses Nunes da Rocha, ulisses.rocha@ufz.de

Table S3. Transcription factors from the metagenome of an anaerobic ammonium oxidizing microbial community from an anammox membrane bioreactor (LAC_MetaG_1) that we mined and hand-curated from a general annotation generated using Prokka (18).

locus_tag	product
**KCPMINPF_00018**	Regulatory protein RecX
**KCPMINPF_00282**	Phosphate regulon transcriptional regulatory protein PhoB
**KCPMINPF_00285**	Transcriptional regulatory protein CusR
**KCPMINPF_00319**	Transcriptional regulatory protein OmpR
**KCPMINPF_00332**	Regulatory protein LuxO
**KCPMINPF_00360**	DnaA regulatory inactivator Hda
**KCPMINPF_00389**	Hydrogen peroxide-inducible genes activator
**KCPMINPF_00667**	Copper-sensing transcriptional repressor CsoR
**KCPMINPF_00697**	Transcriptional regulatory protein QseB
**KCPMINPF_00700**	Transcriptional activator protein AnoR
**KCPMINPF_01226**	Nitrogen regulatory protein
**KCPMINPF_01534**	Transcriptional regulatory protein ZraR
**KCPMINPF_01666**	Bifunctional transcriptional activator/DNA repair enzyme Ada
**KCPMINPF_01678**	Heat-inducible transcription repressor HrcA
**KCPMINPF_01778**	Transcriptional repressor NrdR
**KCPMINPF_01864**	Transcriptional regulatory protein ZraR
**KCPMINPF_01871**	flagellum biosynthesis repressor protein FlbT
**KCPMINPF_01880**	RNA polymerase-binding transcription factor DksA
**KCPMINPF_02102**	Transcriptional activator protein CopR
**KCPMINPF_02113**	Redox-sensing transcriptional repressor Rex 1
**KCPMINPF_02156**	KDP operon transcriptional regulatory protein KdpE
**KCPMINPF_02187**	Transcriptional regulatory protein LnrK
**KCPMINPF_02223**	DNA-binding transcriptional activator DevR/DosR
**KCPMINPF_02225**	Alkaline phosphatase synthesis transcriptional regulatory protein PhoP
**KCPMINPF_02284**	Regulatory protein AtoC
**KCPMINPF_02362**	Regulatory protein RecX
**KCPMINPF_02483**	Heat-inducible transcription repressor HrcA

**KCPMINPF_02687**	Transcriptional regulatory protein LiaR
**KCPMINPF_02701**	Transcriptional regulatory protein LnrK
**KCPMINPF_02805**	Ribose operon repressor
**KCPMINPF_02822**	Transcriptional regulatory protein WalR
**KCPMINPF_02855**	N-acetylglucosamine repressor
**KCPMINPF_02954**	Bifunctional transcriptional activator/DNA repair enzyme Ada
**KCPMINPF_02998**	DNA-binding transcriptional regulator BolA
**KCPMINPF_03057**	LexA repressor
**KCPMINPF_03096**	Sigma factor AlgU regulatory protein MucB
**KCPMINPF_03148**	Regulatory protein AtoC
**KCPMINPF_03226**	Transcriptional repressor NrdR
**KCPMINPF_03275**	Transcriptional regulatory protein BaeR
**KCPMINPF_03362**	Phosphate regulon transcriptional regulatory protein PhoB
**KCPMINPF_03435**	Regulatory protein AtoC
**KCPMINPF_03461**	DNA-binding transcriptional activator HyfR
**KCPMINPF_03514**	Pca regulon regulatory protein
**KCPMINPF_03553**	Phosphate regulon transcriptional regulatory protein PhoB
**KCPMINPF_03610**	Regulatory protein RecX
**KCPMINPF_03643**	Regulatory protein AtoC
**KCPMINPF_03670**	Transcriptional regulator MraZ
**KCPMINPF_03672**	Penicillin-binding protein activator LpoA
**KCPMINPF_03761**	Phosphate regulon transcriptional regulatory protein PhoB
**KCPMINPF_03788**	Transcriptional regulatory protein DegU
**KCPMINPF_03814**	Oxygen regulatory protein NreC
**KCPMINPF_03841**	Transcriptional regulatory protein LiaR
**KCPMINPF_03977**	Ribose operon repressor
**KCPMINPF_04134**	Alkaline phosphatase synthesis transcriptional regulatory protein PhoP
**KCPMINPF_04355**	Iron-dependent repressor IdeR
**KCPMINPF_04394**	Methanol dehydrogenase activator
**KCPMINPF_04549**	Transcriptional regulatory protein DegU
**KCPMINPF_04560**	Transcriptional regulatory protein LiaR
**KCPMINPF_04586**	RNA polymerase-binding transcription factor CarD
**KCPMINPF_04647**	LexA repressor
**KCPMINPF_04672**	DNA-binding transcriptional regulator NtrC
**KCPMINPF_04801**	Transcriptional regulatory protein DegU
**KCPMINPF_04820**	Copper-sensing transcriptional repressor CsoR
**KCPMINPF_04926**	Zinc-specific metallo-regulatory protein
**KCPMINPF_04974**	Transcriptional repressor NrdR
**KCPMINPF_05013**	Transcriptional regulatory protein AfsQ1
**KCPMINPF_05015**	Alkaline phosphatase synthesis transcriptional regulatory protein SphR
**KCPMINPF_05050**	Transcriptional regulatory protein LiaR
**KCPMINPF_05125**	Heat-inducible transcription repressor HrcA
**KCPMINPF_05233**	Transcriptional regulatory protein DegU

**KCPMINPF_05321**	Regulatory protein AtoC
**KCPMINPF_05367**	Alkaline phosphatase synthesis transcriptional regulatory protein PhoP
**KCPMINPF_05405**	Leucine-responsive regulatory protein
**KCPMINPF_05586**	Alkaline phosphatase synthesis transcriptional regulatory protein PhoP
**KCPMINPF_05718**	Transcriptional regulator KdgR
**KCPMINPF_05762**	Regulatory protein RecX
**KCPMINPF_05970**	Transcriptional regulatory protein ComA
**KCPMINPF_06062**	Leucine-responsive regulatory protein
**KCPMINPF_06066**	DNA-binding transcriptional activator DecR
**KCPMINPF_06091**	Transcriptional activator protein NhaR
**KCPMINPF_06212**	Arginine repressor
**KCPMINPF_06233**	RNA polymerase-binding transcription factor DksA
**KCPMINPF_06252**	Nitrogen regulatory protein P-II
**KCPMINPF_06316**	Transcriptional regulatory protein ZraR
**KCPMINPF_06414**	DNA-binding transcriptional regulator NtrC
**KCPMINPF_06592**	Transcriptional regulator SlyA
**KCPMINPF_06679**	Heat-inducible transcription repressor HrcA
**KCPMINPF_06875**	Transcriptional regulatory protein DegU
**KCPMINPF_06907**	ATP phosphoribosyltransferase regulatory subunit
**KCPMINPF_06920**	Fumarate and nitrate reduction regulatory protein
**KCPMINPF_06922**	Transcriptional regulatory protein OmpR
**KCPMINPF_06982**	DNA-binding transcriptional regulator NtrC
**KCPMINPF_07122**	DNA-binding transcriptional regulator BolA
**KCPMINPF_07148**	Transcriptional regulator HilA
**KCPMINPF_07186**	Phosphoenolpyruvate synthase regulatory protein
**KCPMINPF_07242**	Transcriptional regulatory protein OmpR
**KCPMINPF_07483**	Transcriptional activator protein CopR
**KCPMINPF_07529**	Transcriptional regulator MtlR
**KCPMINPF_07659**	Transcriptional regulatory protein WalR
**KCPMINPF_07664**	Alkaline phosphatase synthesis transcriptional regulatory protein PhoP
**KCPMINPF_07670**	Transcriptional regulatory protein BaeR
**KCPMINPF_07839**	Transcriptional regulator LsrR
**KCPMINPF_07913**	Transcriptional regulatory protein QseB
**KCPMINPF_07929**	Photosynthetic apparatus regulatory protein RegA
**KCPMINPF_07947**	Transcriptional regulatory protein OmpR
**KCPMINPF_08029**	Nitrogen regulatory protein P-II
**KCPMINPF_08069**	Transcriptional activator HlyU
**KCPMINPF_08095**	Transcriptional activator protein Anr
**KCPMINPF_08265**	Transcriptional activatory protein AadR
**KCPMINPF_08293**	Transcriptional activator protein Anr
**KCPMINPF_08477**	Alkaline phosphatase synthesis transcriptional regulatory protein PhoP
**KCPMINPF_08914**	Regulatory protein RecX
**KCPMINPF_09287**	Transcriptional regulatory protein OmpR

**KCPMINPF_09336**	Transcriptional regulator MraZ
**KCPMINPF_09794**	Regulatory protein RecX
**KCPMINPF_09875**	LexA repressor
**KCPMINPF_09896**	Heat-inducible transcription repressor HrcA
**KCPMINPF_09913**	Transcriptional regulatory protein CusR
**KCPMINPF_100034**	Nitrogen regulatory protein P-II
**KCPMINPF_100042**	Transcriptional regulatory protein DegU
**KCPMINPF_10006**	Transcriptional repressor NrdR
**KCPMINPF_100095**	Peroxide-responsive repressor PerR
**KCPMINPF_100115**	Oxygen regulatory protein NreC
**KCPMINPF_10023**	Transcriptional regulator MraZ
**KCPMINPF_100316**	Transcriptional regulatory protein LnrK
**KCPMINPF_100329**	Transcriptional regulatory protein CusR
**KCPMINPF_100353**	RNA polymerase-binding transcription factor DksA
**KCPMINPF_100610**	Heat-inducible transcription repressor HrcA
**KCPMINPF_100669**	Regulatory protein MsrR
**KCPMINPF_100805**	Transcriptional activator protein CzcR
**KCPMINPF_100910**	RNA polymerase-binding transcription factor DksA
**KCPMINPF_100923**	Transcriptional regulatory protein LiaR
**KCPMINPF_101018**	Transcriptional regulatory protein WalR
**KCPMINPF_101141**	DnaA regulatory inactivator Hda
**KCPMINPF_101153**	Transcriptional regulatory protein WalR
**KCPMINPF_101238**	Transcriptional activator protein CopR
**KCPMINPF_101305**	Transcriptional repressor MprA
**KCPMINPF_101364**	Mercuric resistance operon regulatory protein
**KCPMINPF_101377**	LexA repressor
**KCPMINPF_101378**	LexA repressor
**KCPMINPF_101479**	Transcriptional regulatory protein LiaR
**KCPMINPF_101497**	RNA polymerase-binding transcription factor DksA
**KCPMINPF_101677**	Heat-inducible transcription repressor HrcA
**KCPMINPF_101709**	Alkaline phosphatase synthesis transcriptional regulatory protein PhoP
**KCPMINPF_101727**	Transcriptional regulatory protein DegU
**KCPMINPF_101778**	Transcriptional regulator SlyA
**KCPMINPF_101802**	Oxygen regulatory protein NreC
**KCPMINPF_101862**	Regulatory protein AtoC
**KCPMINPF_101870**	Bifunctional ligase/repressor BirA
**KCPMINPF_101933**	Transcriptional repressor NrdR
**KCPMINPF_102016**	Transcriptional regulatory protein KdpE
**KCPMINPF_102034**	Transcriptional regulatory protein LiaR
**KCPMINPF_102065**	Alkaline phosphatase synthesis transcriptional regulatory protein SphR
**KCPMINPF_10207**	Transcriptional regulatory protein LiaR
**KCPMINPF_102099**	ATP phosphoribosyltransferase regulatory subunit
**KCPMINPF_102305**	Iron-dependent repressor IdeR

**KCPMINPF_102379**	Alkaline phosphatase synthesis transcriptional regulatory protein PhoP
**KCPMINPF_102454**	Transcriptional regulatory protein tctD
**KCPMINPF_102468**	Transcriptional regulatory protein RcsB
**KCPMINPF_102654**	RNA polymerase-binding transcription factor CarD
**KCPMINPF_102789**	Glucitol operon repressor
**KCPMINPF_102849**	N-acetylglucosamine repressor
**KCPMINPF_102891**	Transcriptional regulatory protein SrrA
**KCPMINPF_102904**	Transcriptional regulatory protein DegU
**KCPMINPF_102960**	Transcriptional regulatory protein LiaR
**KCPMINPF_103119**	Transcriptional regulatory protein LiaR
**KCPMINPF_103161**	LexA repressor
**KCPMINPF_10320**	CdaA regulatory protein CdaR
**KCPMINPF_103220**	Transcriptional regulatory protein SrrA
**KCPMINPF_103306**	Regulatory protein RecX
**KCPMINPF_103311**	Heat-inducible transcription repressor HrcA
**KCPMINPF_103320**	Alkaline phosphatase synthesis transcriptional regulatory protein SphR
**KCPMINPF_103402**	Sensory/regulatory protein RpfC
**KCPMINPF_103414**	Transcriptional regulatory protein PhoP
**KCPMINPF_103575**	Transcriptional regulatory protein OmpR
**KCPMINPF_103583**	Penicillin-binding protein activator LpoA
**KCPMINPF_103609**	Transcriptional regulatory protein ZraR
**KCPMINPF_103616**	Transcriptional activator protein CopR
**KCPMINPF_10392**	Bifunctional ligase/repressor BirA
**KCPMINPF_103943**	Bifunctional transcriptional activator/DNA repair enzyme Ada
**KCPMINPF_103947**	Heat-inducible transcription repressor HrcA
**KCPMINPF_103960**	Glucitol operon repressor
**KCPMINPF_104117**	Mercuric resistance operon regulatory protein
**KCPMINPF_104159**	Transcriptional regulatory protein DegU
**KCPMINPF_104242**	RNA polymerase-binding transcription factor CarD
**KCPMINPF_104270**	Alkaline phosphatase synthesis transcriptional regulatory protein PhoP
**KCPMINPF_104298**	Redox-sensing transcriptional repressor Rex 1
**KCPMINPF_104326**	Murein hydrolase activator EnvC
**KCPMINPF_104435**	Alkaline phosphatase synthesis transcriptional regulatory protein PhoP
**KCPMINPF_104462**	Bifunctional transcriptional activator/DNA repair enzyme Ada
**KCPMINPF_104487**	Transcriptional regulatory protein LiaR
**KCPMINPF_104552**	Transcriptional regulatory protein KdpE
**KCPMINPF_104569**	Transcriptional regulatory protein TcrA
**KCPMINPF_104665**	Transcriptional repressor SmtB
**KCPMINPF_104738**	Redox-sensing transcriptional repressor Rex 1
**KCPMINPF_10476**	Murein hydrolase activator EnvC
**KCPMINPF_104981**	Nitrogen regulatory protein P-II
**KCPMINPF_105078**	KDP operon transcriptional regulatory protein KdpE
**KCPMINPF_105130**	Arginine repressor

**KCPMINPF_105269**	Transcriptional regulatory protein KdpE
**KCPMINPF_105271**	Transcriptional regulatory protein WalR
**KCPMINPF_105397**	Transcriptional regulatory protein DegU
**KCPMINPF_105477**	Anaerobic regulatory protein
**KCPMINPF_105524**	Transcriptional repressor NrdR
**KCPMINPF_105526**	LexA repressor
**KCPMINPF_105654**	Oxygen regulatory protein NreC
**KCPMINPF_105687**	Oxygen regulatory protein NreC
**KCPMINPF_105689**	Transcriptional regulatory protein LiaR
**KCPMINPF_105726**	Phosphate regulon transcriptional regulatory protein PhoB
**KCPMINPF_105761**	Transcriptional regulatory protein DegU
**KCPMINPF_105781**	Transcriptional regulatory protein DegU
**KCPMINPF_106058**	Heat-inducible transcription repressor HrcA
**KCPMINPF_106090**	Transcriptional regulatory protein KdpE
**KCPMINPF_106230**	Transcriptional regulatory protein LiaR
**KCPMINPF_106231**	Transcriptional regulatory protein LnrK
**KCPMINPF_106242**	Transcriptional regulatory protein LiaR
**KCPMINPF_106254**	Transcriptional repressor SmtB
**KCPMINPF_106318**	Transcriptional repressor SmtB
**KCPMINPF_106346**	Alkaline phosphatase synthesis transcriptional regulatory protein PhoP
**KCPMINPF_106570**	RNA polymerase-binding transcription factor DksA
**KCPMINPF_106683**	Transcriptional regulatory protein CusR
**KCPMINPF_106705**	Transcriptional regulatory protein DegU
**KCPMINPF_106737**	Transcriptional regulatory protein LiaR
**KCPMINPF_106899**	RNA polymerase-binding transcription factor CarD
**KCPMINPF_107106**	Transcriptional regulatory protein DegU
**KCPMINPF_107190**	Copper-sensing transcriptional repressor CsoR
**KCPMINPF_107208**	Alkaline phosphatase synthesis transcriptional regulatory protein PhoP
**KCPMINPF_107323**	Transcriptional regulatory protein HprR
**KCPMINPF_107451**	Photosynthetic apparatus regulatory protein RegA
**KCPMINPF_107488**	Ribose operon repressor
**KCPMINPF_107499**	Ribose operon repressor
**KCPMINPF_107571**	Transcriptional regulatory protein KdpE
**KCPMINPF_107640**	Regulatory protein AtoC
**KCPMINPF_107768**	Regulatory protein AtoC
**KCPMINPF_108102**	Alkaline phosphatase synthesis transcriptional regulatory protein PhoP
**KCPMINPF_108151**	LexA repressor
**KCPMINPF_108220**	LexA repressor
**KCPMINPF_108366**	Alkaline phosphatase synthesis transcriptional regulatory protein PhoP
**KCPMINPF_108450**	Alkaline phosphatase synthesis transcriptional regulatory protein SphR
**KCPMINPF_10847**	Transcriptional regulatory protein AfsQ1
**KCPMINPF_10855**	Transcriptional regulator MraZ
**KCPMINPF_108655**	Transcriptional regulatory protein LiaR

**KCPMINPF_108708**	Transcriptional regulatory protein LnrK
**KCPMINPF_108769**	Transcriptional regulatory protein OmpR
**KCPMINPF_108884**	Redox-sensing transcriptional repressor Rex
**KCPMINPF_108939**	Transcriptional regulatory protein OmpR
**KCPMINPF_109002**	RNA polymerase-binding transcription factor DksA
**KCPMINPF_109053**	Transcriptional regulatory protein LiaR
**KCPMINPF_10930**	Alkaline phosphatase synthesis transcriptional regulatory protein PhoP
**KCPMINPF_109347**	Transcriptional regulatory protein DegU
**KCPMINPF_109374**	Bifunctional transcriptional activator/DNA repair enzyme Ada
**KCPMINPF_109620**	Transcriptional regulatory protein DesR
**KCPMINPF_109831**	Oxygen regulatory protein NreC
**KCPMINPF_10988**	Alkaline phosphatase synthesis transcriptional regulatory protein PhoP
**KCPMINPF_110015**	Oxygen regulatory protein NreC
**KCPMINPF_110035**	Transcriptional repressor PaaX
**KCPMINPF_110160**	DNA-binding transcriptional activator DevR/DosR
**KCPMINPF_110161**	Alkaline phosphatase synthesis transcriptional regulatory protein PhoP
**KCPMINPF_110195**	Transcriptional regulatory protein LiaR
**KCPMINPF_110206**	Transcriptional regulatory protein LiaR
**KCPMINPF_110415**	Transcriptional regulatory protein WalR
**KCPMINPF_110494**	Transcriptional regulatory protein WalR
**KCPMINPF_110495**	DNA-binding transcriptional activator DevR/DosR
**KCPMINPF_110502**	Oxygen regulatory protein NreC
**KCPMINPF_110589**	Penicillinase repressor
**KCPMINPF_110614**	Met repressor
**KCPMINPF_110785**	Transcriptional regulatory protein LiaR
**KCPMINPF_110823**	Mercuric resistance operon regulatory protein
**KCPMINPF_111024**	Lactose operon repressor
**KCPMINPF_111032**	Methanol dehydrogenase activator
**KCPMINPF_111040**	Redox-sensing transcriptional repressor Rex 1
**KCPMINPF_111071**	DNA-binding transcriptional regulator NtrC
**KCPMINPF_111189**	Regulatory protein AtoC
**KCPMINPF_111507**	Phosphate regulon transcriptional regulatory protein PhoB
**KCPMINPF_111546**	Nitrogen regulatory protein P-II 1
**KCPMINPF_111590**	Nitrogen regulatory protein
**KCPMINPF_111620**	Transcriptional activator HlyU
**KCPMINPF_11170**	Alkaline phosphatase synthesis transcriptional regulatory protein PhoP
**KCPMINPF_111719**	Oxygen regulatory protein NreC
**KCPMINPF_111761**	LexA repressor
**KCPMINPF_111788**	Transcriptional regulatory protein DegU
**KCPMINPF_111821**	Lactose operon repressor
**KCPMINPF_111944**	Alkaline phosphatase synthesis transcriptional regulatory protein PhoP
**KCPMINPF_111950**	Transcriptional regulatory protein PmpR
**KCPMINPF_112134**	Transcriptional regulatory protein FixJ

**KCPMINPF_112174**	Glucitol operon repressor
**KCPMINPF_112259**	Transcriptional regulatory protein LiaR
**KCPMINPF_11242**	Redox-sensing transcriptional repressor Rex
**KCPMINPF_112522**	Ribose operon repressor
**KCPMINPF_112596**	Transcriptional regulatory protein WalR
**KCPMINPF_112710**	Transcriptional regulatory protein DegU
**KCPMINPF_113414**	Transcriptional regulator KdgR
**KCPMINPF_11354**	ATP phosphoribosyltransferase regulatory subunit
**KCPMINPF_113549**	DNA-binding transcriptional activator DecR
**KCPMINPF_113563**	Oxygen regulatory protein NreC
**KCPMINPF_113604**	Ribose operon repressor
**KCPMINPF_113719**	Photosynthetic apparatus regulatory protein RegA
**KCPMINPF_113728**	Redox-sensing transcriptional repressor Rex 1
**KCPMINPF_113817**	Alkaline phosphatase synthesis transcriptional regulatory protein PhoP
**KCPMINPF_113903**	Regulatory protein RecX
**KCPMINPF_114045**	Arabinose metabolism transcriptional repressor
**KCPMINPF_114059**	DNA-binding transcriptional activator DecR
**KCPMINPF_11409**	Phosphate regulon transcriptional regulatory protein PhoB
**KCPMINPF_114149**	Transcriptional regulatory protein WalR
**KCPMINPF_114166**	Transcriptional regulatory protein DegU
**KCPMINPF_114168**	Regulatory protein AtoC
**KCPMINPF_114246**	Transcriptional regulatory protein SrrA
**KCPMINPF_114288**	Transcriptional regulatory protein ZraR
**KCPMINPF_114301**	Iron-dependent repressor IdeR
**KCPMINPF_114346**	CdaA regulatory protein CdaR
**KCPMINPF_114474**	Transcriptional regulatory protein LiaR
**KCPMINPF_114527**	Mercuric resistance operon regulatory protein
**KCPMINPF_114636**	Oxygen regulatory protein NreC
**KCPMINPF_114639**	ATP phosphoribosyltransferase regulatory subunit
**KCPMINPF_114748**	Transcriptional regulator LsrR
**KCPMINPF_11475**	Transcriptional regulator MraZ
**KCPMINPF_114785**	Transcriptional activator protein CzcR
**KCPMINPF_114921**	Alkaline phosphatase synthesis transcriptional regulatory protein PhoP
**KCPMINPF_115006**	Transcriptional regulatory protein KdpE
**KCPMINPF_115101**	Transcriptional regulatory protein KdpE
**KCPMINPF_115108**	Transcriptional regulatory protein KdpE
**KCPMINPF_115229**	Hca operon transcriptional activator HcaR
**KCPMINPF_115412**	Oxygen regulatory protein NreC
**KCPMINPF_115453**	N-acetylglucosamine repressor
**KCPMINPF_115539**	Transcriptional regulatory protein KdpE
**KCPMINPF_115559**	Transcriptional regulatory protein LiaR
**KCPMINPF_115775**	Methanol dehydrogenase activator
**KCPMINPF_115788**	Transcriptional regulatory protein DegU

**KCPMINPF_115807**	Transcriptional regulatory protein LiaR
**KCPMINPF_115950**	Transcriptional regulatory protein SrrA
**KCPMINPF_116004**	Transcriptional regulatory protein WalR
**KCPMINPF_116084**	Bifunctional transcriptional activator/DNA repair enzyme Ada
**KCPMINPF_116514**	N-acetylglucosamine repressor
**KCPMINPF_116617**	Alkaline phosphatase synthesis transcriptional regulatory protein SphR
**KCPMINPF_116631**	Redox-sensing transcriptional repressor Rex
**KCPMINPF_116642**	Regulatory protein RecX
**KCPMINPF_116647**	Arabinose operon regulatory protein
**KCPMINPF_116656**	Transcriptional regulator MraZ
**KCPMINPF_116846**	Redox-sensing transcriptional repressor Rex 1
**KCPMINPF_116879**	Transcriptional regulatory protein LnrK
**KCPMINPF_116950**	Transcriptional regulator SlyA
**KCPMINPF_11700**	LexA repressor
**KCPMINPF_117261**	Transcriptional repressor NrdR
**KCPMINPF_117602**	Transcriptional regulatory protein OmpR
**KCPMINPF_117837**	Transcriptional regulatory protein SrrA
**KCPMINPF_117889**	Transcriptional regulator WhiB
**KCPMINPF_117977**	Transcriptional regulatory protein LiaR
**KCPMINPF_118040**	Copper-sensing transcriptional repressor CsoR
**KCPMINPF_118147**	Transcriptional activator protein CopR
**KCPMINPF_118195**	Ribose operon repressor
**KCPMINPF_118329**	Pca regulon regulatory protein
**KCPMINPF_11833**	Transcriptional regulatory protein PmpR
**KCPMINPF_118357**	Oxygen regulatory protein NreC
**KCPMINPF_118514**	Transcriptional regulatory protein CseB
**KCPMINPF_118639**	Transcriptional regulatory protein LnrK
**KCPMINPF_118865**	Phosphate regulon transcriptional regulatory protein PhoB
**KCPMINPF_118895**	Glucitol operon repressor
**KCPMINPF_118955**	Alkaline phosphatase synthesis transcriptional regulatory protein PhoP
**KCPMINPF_119051**	Transcriptional regulatory protein WalR
**KCPMINPF_119154**	Alkaline phosphatase synthesis transcriptional regulatory protein PhoP
**KCPMINPF_119170**	Pca regulon regulatory protein
**KCPMINPF_119292**	Oxygen regulatory protein NreC
**KCPMINPF_119381**	Heat-inducible transcription repressor HrcA
**KCPMINPF_119499**	Transcriptional regulatory protein DegU
**KCPMINPF_119513**	Alkaline phosphatase synthesis transcriptional regulatory protein PhoP
**KCPMINPF_119586**	Oxygen regulatory protein NreC
**KCPMINPF_119628**	Nitrogen regulatory protein P-II
**KCPMINPF_119778**	RNA polymerase-binding transcription factor CarD
**KCPMINPF_119911**	Alkaline phosphatase synthesis transcriptional regulatory protein SphR
**KCPMINPF_120191**	Transcriptional regulatory protein DegU
**KCPMINPF_120206**	Oxygen regulatory protein NreC

**KCPMINPF_120404**	Luminescence regulatory protein LuxO
**KCPMINPF_120608**	Regulatory protein RecX
**KCPMINPF_120717**	Regulatory protein AtoC
**KCPMINPF_120847**	Nitrogen regulatory protein
**KCPMINPF_121039**	Transcriptional regulator KdgR
**KCPMINPF_121057**	Transcriptional regulatory protein LiaR
**KCPMINPF_121216**	Oxygen regulatory protein NreC
**KCPMINPF_121221**	Transcriptional regulatory protein LiaR
**KCPMINPF_12133**	Methanol dehydrogenase activator
**KCPMINPF_121435**	Phosphoenolpyruvate synthase regulatory protein
**KCPMINPF_121528**	Regulatory protein RecX
**KCPMINPF_121692**	Transcriptional activator protein CopR
**KCPMINPF_121769**	Regulatory protein RecX
**KCPMINPF_12193**	Regulatory protein AtoC
**KCPMINPF_121943**	Transcriptional regulatory protein LiaR
**KCPMINPF_121994**	Transcriptional regulatory protein LiaR
**KCPMINPF_122008**	Transcriptional regulatory protein WalR
**KCPMINPF_12210**	Regulatory protein AtoC
**KCPMINPF_122260**	Heat-inducible transcription repressor HrcA
**KCPMINPF_122403**	Oxygen regulatory protein NreC
**KCPMINPF_122591**	Nitrogen regulatory protein P-II 2
**KCPMINPF_122607**	Redox-sensing transcriptional repressor Rex 1
**KCPMINPF_122739**	Regulatory protein AtoC
**KCPMINPF_122778**	Transcriptional repressor NrdR
**KCPMINPF_122819**	N-acetylglucosamine repressor
**KCPMINPF_122942**	Mercuric resistance operon regulatory protein
**KCPMINPF_122959**	Leucine-responsive regulatory protein
**KCPMINPF_123215**	Alkaline phosphatase synthesis transcriptional regulatory protein PhoP
**KCPMINPF_123255**	Transcriptional regulatory protein QseB
**KCPMINPF_123322**	Transcriptional regulatory protein LiaR
**KCPMINPF_123457**	Copper-sensing transcriptional repressor CsoR
**KCPMINPF_123484**	Transcriptional regulatory protein ros
**KCPMINPF_123501**	Alkaline phosphatase synthesis transcriptional regulatory protein PhoP
**KCPMINPF_123573**	Transcriptional regulatory protein WalR
**KCPMINPF_12359**	Alkaline phosphatase synthesis transcriptional regulatory protein PhoP
**KCPMINPF_123664**	KDP operon transcriptional regulatory protein KdpE
**KCPMINPF_123738**	Alkaline phosphatase synthesis transcriptional regulatory protein PhoP
**KCPMINPF_123751**	Transcriptional regulatory protein LiaR
**KCPMINPF_12437**	Alkaline phosphatase synthesis transcriptional regulatory protein PhoP
**KCPMINPF_124548**	Transcriptional regulatory protein LiaR
**KCPMINPF_124609**	Negative regulatory protein YxlE
**KCPMINPF_124668**	Transcriptional regulatory protein BaeR
**KCPMINPF_124695**	Alkaline phosphatase synthesis transcriptional regulatory protein PhoP

**KCPMINPF_12480**	Transcriptional repressor SmtB
**KCPMINPF_124866**	Transcriptional regulatory protein ZraR
**KCPMINPF_124969**	Transcriptional regulatory protein OmpR
**KCPMINPF_12504**	Peroxide-responsive repressor PerR
**KCPMINPF_125113**	Leucine-responsive regulatory protein
**KCPMINPF_125287**	Lactose operon repressor
**KCPMINPF_125348**	Lactose operon repressor
**KCPMINPF_125443**	Transcriptional regulatory protein TcrA
**KCPMINPF_125480**	Leucine-responsive regulatory protein
**KCPMINPF_12549**	Transcriptional activator protein CzcR
**KCPMINPF_125505**	Transcriptional regulatory protein WalR
**KCPMINPF_125509**	Transcriptional regulatory protein DegU
**KCPMINPF_12562**	Heat-inducible transcription repressor HrcA
**KCPMINPF_125639**	LexA repressor
**KCPMINPF_12569**	Pur operon repressor
**KCPMINPF_125704**	Transcriptional regulatory protein LnrK
**KCPMINPF_125716**	Hydrogen peroxide-inducible genes activator
**KCPMINPF_125723**	Oxygen regulatory protein NreC
**KCPMINPF_125822**	Bifunctional ligase/repressor BirA
**KCPMINPF_125902**	Transcriptional regulatory protein QseB
**KCPMINPF_125919**	Transcriptional regulatory protein LiaR
**KCPMINPF_125931**	Alkaline phosphatase synthesis transcriptional regulatory protein PhoP
**KCPMINPF_12600**	PTS-dependent dihydroxyacetone kinase operon regulatory protein
**KCPMINPF_126061**	Transcriptional regulatory protein DegU
**KCPMINPF_126544**	Oxygen regulatory protein NreC
**KCPMINPF_126575**	Alkaline phosphatase synthesis transcriptional regulatory protein SphR
**KCPMINPF_126703**	DnaA regulatory inactivator Hda
**KCPMINPF_126714**	Negative regulatory protein YxlE
**KCPMINPF_12685**	Biofilm growth-associated repressor
**KCPMINPF_126913**	Murein hydrolase activator NlpD
**KCPMINPF_12692**	Oxygen regulatory protein NreC
**KCPMINPF_126938**	Alkaline phosphatase synthesis transcriptional regulatory protein PhoP
**KCPMINPF_12703**	Transcriptional regulatory protein DegU
**KCPMINPF_127153**	Virulence transcriptional regulatory protein PhoP
**KCPMINPF_127157**	LexA repressor
**KCPMINPF_127326**	RNA polymerase-binding transcription factor CarD
**KCPMINPF_127452**	Sigma54-dependent transcriptional activator SfnR
**KCPMINPF_12751**	Transcriptional regulatory protein WalR
**KCPMINPF_127551**	Alkaline phosphatase synthesis transcriptional regulatory protein PhoP
**KCPMINPF_127577**	Copper-sensing transcriptional repressor CsoR
**KCPMINPF_12764**	Regulatory protein RecX
**KCPMINPF_12769**	Transcriptional regulatory protein WalR
**KCPMINPF_127693**	Transcriptional regulatory protein WalR

**KCPMINPF_127695**	Regulatory protein AtoC
**KCPMINPF_127704**	Tetrathionate response regulatory protein TtrR
**KCPMINPF_127705**	Transcriptional regulatory protein TdiR
**KCPMINPF_127719**	Transcriptional activator NphR
**KCPMINPF_127749**	KDP operon transcriptional regulatory protein KdpE
**KCPMINPF_127815**	Transcriptional regulatory protein DegU
**KCPMINPF_127849**	Organic hydroperoxide resistance transcriptional regulator
**KCPMINPF_128038**	Transcriptional regulatory protein BaeR
**KCPMINPF_128112**	DNA-binding transcriptional regulator NtrC
**KCPMINPF_128143**	Transcriptional repressor NrdR
**KCPMINPF_128215**	Regulatory protein AtoC
**KCPMINPF_128269**	Transcriptional regulatory protein LiaR
**KCPMINPF_12827**	Transcriptional regulatory protein DegU
**KCPMINPF_128398**	Alkaline phosphatase synthesis transcriptional regulatory protein PhoP
**KCPMINPF_128508**	DNA-binding transcriptional activator DecR
**KCPMINPF_12851**	Transcriptional regulatory protein LnrK
**KCPMINPF_128534**	Alkaline phosphatase synthesis transcriptional regulatory protein PhoP
**KCPMINPF_128583**	Transcriptional regulator MraZ
**KCPMINPF_128812**	Transcriptional regulatory protein WalR
**KCPMINPF_128822**	(R)-phenyllactate dehydratase activator
**KCPMINPF_128823**	(R)-phenyllactate dehydratase activator
**KCPMINPF_128828**	Nitrogen regulatory protein P-II 2
**KCPMINPF_128901**	Alkaline phosphatase synthesis transcriptional regulatory protein PhoP
**KCPMINPF_128958**	Regulatory protein RecX
**KCPMINPF_129107**	Transcriptional regulatory protein ZraR
**KCPMINPF_129181**	Biofilm growth-associated repressor
**KCPMINPF_129283**	Transcriptional regulator LsrR
**KCPMINPF_129352**	Transcriptional repressor SmtB
**KCPMINPF_129576**	KDP operon transcriptional regulatory protein KdpE
**KCPMINPF_129684**	Sensory/regulatory protein RpfC
**KCPMINPF_129703**	Alkaline phosphatase synthesis transcriptional regulatory protein PhoP
**KCPMINPF_129932**	Transcriptional regulatory protein LiaR
**KCPMINPF_130040**	Lactose operon repressor
**KCPMINPF_130063**	Hydrogen peroxide-inducible genes activator
**KCPMINPF_130107**	Transcriptional regulatory protein DegU
**KCPMINPF_130409**	Heat-inducible transcription repressor HrcA
**KCPMINPF_130485**	Purine catabolism regulatory protein
**KCPMINPF_130501**	Transcriptional activator HlyU
**KCPMINPF_130542**	Transcriptional regulatory protein BtsR
**KCPMINPF_130547**	Bifunctional transcriptional activator/DNA repair enzyme Ada
**KCPMINPF_131121**	Transcriptional regulatory protein DegU
**KCPMINPF_131188**	Transcriptional regulatory protein DegU
**KCPMINPF_131403**	N-acetylglucosamine repressor

**KCPMINPF_131637**	Regulatory protein Spx
**KCPMINPF_131749**	Transcriptional activator protein CopR
**KCPMINPF_131818**	Nitrogen regulatory protein P-II
**KCPMINPF_131845**	LexA repressor
**KCPMINPF_132017**	Gliding motility regulatory protein
**KCPMINPF_132026**	Transcriptional regulator BlaI
**KCPMINPF_132028**	LexA repressor
**KCPMINPF_132283**	LexA repressor
**KCPMINPF_13232**	Transcriptional regulatory protein DegU
**KCPMINPF_132332**	Bifunctional ligase/repressor BirA
**KCPMINPF_132515**	LexA repressor
**KCPMINPF_132581**	Glycine cleavage system transcriptional activator
**KCPMINPF_13262**	Murein hydrolase activator EnvC
**KCPMINPF_132657**	Pyruvate dehydrogenase complex repressor
**KCPMINPF_132848**	Transcriptional regulator AcuR
**KCPMINPF_132880**	DNA-binding transcriptional regulator BolA
**KCPMINPF_132895**	Transcriptional regulatory protein KdpE
**KCPMINPF_132914**	Transcriptional regulatory protein WalR
**KCPMINPF_132964**	Glycine cleavage system transcriptional activator
**KCPMINPF_133106**	DNA-binding transcriptional regulator BolA
**KCPMINPF_13319**	Leucine-responsive regulatory protein
**KCPMINPF_133232**	Transcriptional activator HlyU
**KCPMINPF_133800**	Transcriptional regulatory protein LiaR
**KCPMINPF_133815**	Transcriptional regulatory protein LiaR
**KCPMINPF_13383**	Transcriptional regulatory protein ros
**KCPMINPF_133846**	Transcriptional regulatory protein ZraR
**KCPMINPF_133923**	RNA polymerase-binding transcription factor CarD
**KCPMINPF_133925**	Transcriptional regulatory protein ZraR
**KCPMINPF_13405**	Transcriptional regulatory protein OmpR
**KCPMINPF_134149**	RNA polymerase-binding transcription factor DksA
**KCPMINPF_134226**	Phosphate regulon transcriptional regulatory protein PhoB
**KCPMINPF_134257**	N-acetylglucosamine repressor
**KCPMINPF_134284**	N-acetylglucosamine repressor
**KCPMINPF_13453**	DNA-binding transcriptional regulator NtrC
**KCPMINPF_13455**	DNA-binding transcriptional regulator NtrC
**KCPMINPF_13480**	LexA repressor
**KCPMINPF_134831**	RNA polymerase-binding transcription factor CarD
**KCPMINPF_134839**	Alkaline phosphatase synthesis transcriptional regulatory protein SphR
**KCPMINPF_135134**	Nitrogen regulatory protein P-II
**KCPMINPF_135145**	Ribose operon repressor
**KCPMINPF_135163**	Transcriptional regulatory protein BtsR
**KCPMINPF_13523**	Transcriptional regulatory protein AfsQ1
**KCPMINPF_135283**	Transcriptional regulatory protein CusR

**KCPMINPF_135308**	Transcriptional regulatory protein LiaR
**KCPMINPF_135424**	N-acetylglucosamine repressor
**KCPMINPF_135501**	Transcriptional regulatory protein DesR
**KCPMINPF_135513**	Transcriptional regulator SlyA
**KCPMINPF_135554**	Transcriptional repressor IclR
**KCPMINPF_135560**	Sensory/regulatory protein RpfC
**KCPMINPF_135697**	N-acetylglucosamine repressor
**KCPMINPF_135737**	N-acetylglucosamine repressor
**KCPMINPF_13597**	Bifunctional ligase/repressor BirA
**KCPMINPF_135982**	Ribose operon repressor
**KCPMINPF_136083**	Oxygen regulatory protein NreC
**KCPMINPF_136128**	Transcriptional regulatory protein WalR
**KCPMINPF_136192**	Regulatory protein AtoC
**KCPMINPF_136265**	Transcriptional regulatory protein BtsR
**KCPMINPF_136286**	Transcriptional regulatory protein WalR
**KCPMINPF_13633**	Nitrogen regulatory protein P-II
**KCPMINPF_136389**	Transcriptional activatory protein BadR
**KCPMINPF_136562**	Transcriptional regulatory protein WalR
**KCPMINPF_136642**	RNA polymerase-binding transcription factor DksA
**KCPMINPF_136658**	Transcriptional regulator KdgR
**KCPMINPF_136714**	RNA polymerase-binding transcription factor DksA
**KCPMINPF_136860**	Bifunctional ligase/repressor BirA
**KCPMINPF_136869**	Psp operon transcriptional activator
**KCPMINPF_136973**	Transcriptional regulatory protein DesR
**KCPMINPF_136976**	Transcriptional repressor NrdR
**KCPMINPF_137078**	Transcriptional regulatory protein PrrA
**KCPMINPF_137144**	Transcriptional repressor SmtB
**KCPMINPF_137243**	Methanol dehydrogenase activator
**KCPMINPF_13727**	RNA polymerase-binding transcription factor DksA
**KCPMINPF_137271**	Transcriptional regulatory protein LnrK
**KCPMINPF_137304**	Transcriptional regulatory protein DegU
**KCPMINPF_137305**	Transcriptional regulatory protein DegU
**KCPMINPF_13743**	Regulatory protein AtoC
**KCPMINPF_13745**	Transcriptional regulatory protein AfsQ1
**KCPMINPF_137549**	Peroxide-responsive repressor PerR
**KCPMINPF_137688**	DNA-binding transcriptional regulator BolA
**KCPMINPF_137894**	Transcriptional regulatory protein QseB
**KCPMINPF_137930**	Phosphate regulon transcriptional regulatory protein PhoB
**KCPMINPF_138306**	Phosphate regulon transcriptional regulatory protein PhoB
**KCPMINPF_138547**	Bifunctional transcriptional activator/DNA repair enzyme Ada
**KCPMINPF_138564**	Glycerol-3-phosphate regulon repressor
**KCPMINPF_138598**	Transcriptional activator protein CzcR
**KCPMINPF_138636**	LexA repressor

**KCPMINPF_138749**	Transcriptional regulatory protein OmpR
**KCPMINPF_139117**	Gliding motility regulatory protein
**KCPMINPF_139149**	Transcriptional repressor NrdR
**KCPMINPF_139211**	Transcriptional regulatory protein WalR
**KCPMINPF_139235**	Alkaline phosphatase synthesis transcriptional regulatory protein SphR
**KCPMINPF_139246**	Nitrogen regulatory protein P-II
**KCPMINPF_139287**	Lactose operon repressor
**KCPMINPF_139367**	Transcriptional repressor FrmR
**KCPMINPF_139515**	Transcriptional regulatory protein KdpE
**KCPMINPF_139725**	Transcriptional regulatory protein BaeR
**KCPMINPF_139990**	Transcriptional regulatory protein DegU
**KCPMINPF_140269**	Pca regulon regulatory protein
**KCPMINPF_140489**	Alkaline phosphatase synthesis transcriptional regulatory protein PhoP
**KCPMINPF_140560**	Alkaline phosphatase synthesis transcriptional regulatory protein PhoP
**KCPMINPF_140567**	Glc operon transcriptional activator
**KCPMINPF_140687**	Glycerol-3-phosphate regulon repressor
**KCPMINPF_14079**	LexA repressor
**KCPMINPF_141147**	Transcriptional regulatory protein BaeR
**KCPMINPF_14116**	Regulatory protein AtoC
**KCPMINPF_141281**	Transcriptional activatory protein AadR
**KCPMINPF_14134**	LexA repressor
**KCPMINPF_141399**	Transcriptional regulatory protein ros
**KCPMINPF_141449**	Transcriptional regulatory protein WalR
**KCPMINPF_141698**	Transcriptional repressor PaaX
**KCPMINPF_14176**	Phosphate regulon transcriptional regulatory protein PhoB
**KCPMINPF_141857**	Phosphoglycerate transport regulatory protein PgtC
**KCPMINPF_141913**	Transcriptional regulatory protein FixJ
**KCPMINPF_142038**	Nitrogen regulatory protein P-II
**KCPMINPF_142106**	Heat-inducible transcription repressor HrcA
**KCPMINPF_142257**	Sensory/regulatory protein RpfC
**KCPMINPF_142350**	Transcriptional regulatory protein WalR
**KCPMINPF_142393**	Transcriptional activator protein CopR
**KCPMINPF_142440**	Regulatory protein RecX
**KCPMINPF_142567**	Transcriptional regulatory protein DegU
**KCPMINPF_142654**	Copper-sensing transcriptional repressor CsoR
**KCPMINPF_142789**	Tetracycline repressor protein class G
**KCPMINPF_142884**	Nitrogen regulatory protein P-II 2
**KCPMINPF_14294**	Transcriptional regulatory protein ZraR
**KCPMINPF_143054**	Regulatory protein AtoC
**KCPMINPF_143491**	Heat-inducible transcription repressor HrcA
**KCPMINPF_143512**	DNA-binding transcriptional regulator NtrC
**KCPMINPF_143577**	Iron-dependent repressor IdeR
**KCPMINPF_143595**	Glucitol operon repressor

**KCPMINPF_143615**	Penicillinase repressor
**KCPMINPF_143700**	Transcriptional regulatory protein DegU
**KCPMINPF_143802**	Heat-inducible transcription repressor HrcA
**KCPMINPF_143830**	Oxygen regulatory protein NreC
**KCPMINPF_143868**	Oxygen regulatory protein NreC
**KCPMINPF_144241**	Transcriptional regulatory protein WalR
**KCPMINPF_144359**	Sigma factor AlgU regulatory protein MucB
**KCPMINPF_144369**	Extracellular matrix regulatory protein A
**KCPMINPF_144413**	Transcriptional regulator KdgR
**KCPMINPF_144427**	ATP phosphoribosyltransferase regulatory subunit
**KCPMINPF_144473**	Bifunctional transcriptional activator/DNA repair enzyme Ada
**KCPMINPF_144595**	Peroxide-responsive repressor PerR
**KCPMINPF_144598**	Peroxide-responsive repressor PerR
**KCPMINPF_14481**	Methanol dehydrogenase activator
**KCPMINPF_144829**	Transcriptional repressor NrdR
**KCPMINPF_145066**	Transcriptional regulator MraZ
**KCPMINPF_145144**	Mercuric resistance operon regulatory protein
**KCPMINPF_14551**	LexA repressor
**KCPMINPF_145550**	Nitrogen regulatory protein P-II 1
**KCPMINPF_145586**	Hydrogen peroxide-inducible genes activator
**KCPMINPF_14562**	Regulatory protein RecX
**KCPMINPF_145750**	Transcriptional regulatory protein KdpE
**KCPMINPF_145808**	N-acetylglucosamine repressor
**KCPMINPF_146099**	Glc operon transcriptional activator
**KCPMINPF_146184**	Transcriptional regulatory protein WalR
**KCPMINPF_146314**	Glycine cleavage system transcriptional activator
**KCPMINPF_14635**	Copper-sensing transcriptional repressor CsoR
**KCPMINPF_146468**	Transcriptional regulatory protein OmpR
**KCPMINPF_146557**	LexA repressor
**KCPMINPF_146618**	Bifunctional ligase/repressor BirA
**KCPMINPF_146664**	Transcriptional regulatory protein WalR
**KCPMINPF_146842**	Hydrogen peroxide-inducible genes activator
**KCPMINPF_146955**	Iron-dependent repressor IdeR
**KCPMINPF_146956**	Iron-dependent repressor IdeR
**KCPMINPF_147142**	Hca operon transcriptional activator HcaR
**KCPMINPF_147332**	RNA polymerase-binding transcription factor DksA
**KCPMINPF_147445**	Transcriptional repressor SmtB
**KCPMINPF_147542**	Transcriptional regulatory protein OmpR
**KCPMINPF_14755**	Transcriptional regulatory protein WalR
**KCPMINPF_147596**	Transcriptional repressor NrdR
**KCPMINPF_147643**	Alkaline phosphatase synthesis transcriptional regulatory protein SphR
**KCPMINPF_147917**	Transcriptional regulatory protein KdpE
**KCPMINPF_148065**	CdaA regulatory protein CdaR

**KCPMINPF_148087**	Denitrification regulatory protein NirQ
**KCPMINPF_148188**	Oxygen regulatory protein NreC
**KCPMINPF_148630**	Ribose operon repressor
**KCPMINPF_148695**	Glycerol-3-phosphate regulon repressor
**KCPMINPF_148917**	Transcriptional repressor SmtB
**KCPMINPF_14896**	Transcriptional regulatory protein ZraR
**KCPMINPF_149005**	Transcriptional regulator SlyA
**KCPMINPF_149006**	Transcriptional regulator SlyA
**KCPMINPF_149057**	Transcriptional activator protein CopR
**KCPMINPF_149204**	Transcriptional regulatory protein WalR
**KCPMINPF_149398**	Transcriptional regulatory protein WalR
**KCPMINPF_14943**	Transcriptional regulatory protein BtsR
**KCPMINPF_14947**	Regulatory protein AtoC
**KCPMINPF_149514**	Alkaline phosphatase synthesis transcriptional regulatory protein PhoP
**KCPMINPF_149655**	Alkaline phosphatase synthesis transcriptional regulatory protein PhoP
**KCPMINPF_149735**	Oxygen regulatory protein NreC
**KCPMINPF_149777**	Transcriptional regulatory protein LnrK
**KCPMINPF_149901**	Transcriptional regulatory protein DegU
**KCPMINPF_15028**	Denitrification regulatory protein NirQ
**KCPMINPF_150351**	Leucine-responsive regulatory protein
**KCPMINPF_150389**	cAMP-activated global transcriptional regulator CRP
**KCPMINPF_150558**	Transcriptional repressor NrdR
**KCPMINPF_15065**	Hydrogen peroxide-inducible genes activator
**KCPMINPF_150753**	Bifunctional ligase/repressor BirA
**KCPMINPF_150894**	Transcriptional regulatory protein PhoP
**KCPMINPF_15128**	Transcriptional regulatory protein YpdB
**KCPMINPF_151416**	LexA repressor
**KCPMINPF_151463**	KDP operon transcriptional regulatory protein KdpE
**KCPMINPF_151511**	Transcriptional regulatory protein HprR
**KCPMINPF_151634**	Bifunctional ligase/repressor BirA
**KCPMINPF_151665**	Alkaline phosphatase synthesis transcriptional regulatory protein PhoP
**KCPMINPF_15171**	Murein hydrolase activator EnvC
**KCPMINPF_151748**	Exoenzyme S synthesis regulatory protein ExsA
**KCPMINPF_151779**	Transcriptional regulatory protein DegU
**KCPMINPF_151856**	Transcriptional regulatory protein FixJ
**KCPMINPF_151857**	Transcriptional regulatory protein TdiR
**KCPMINPF_151931**	Heat-inducible transcription repressor HrcA
**KCPMINPF_152113**	Sensory/regulatory protein RpfC
**KCPMINPF_15215**	Phosphate regulon transcriptional regulatory protein PhoB
**KCPMINPF_15219**	Transcriptional regulatory protein LiaR
**KCPMINPF_152919**	Transcriptional regulator MraZ
**KCPMINPF_152979**	Oxygen regulatory protein NreC
**KCPMINPF_15309**	Transcriptional regulatory protein KdpE

**KCPMINPF_153251**	Nitrogen regulatory protein P-II 2
**KCPMINPF_153398**	Bifunctional ligase/repressor BirA
**KCPMINPF_153675**	ATP phosphoribosyltransferase regulatory subunit
**KCPMINPF_153707**	Heat-inducible transcription repressor HrcA
**KCPMINPF_15431**	Transcriptional regulatory protein ZraR
**KCPMINPF_154332**	Oxygen regulatory protein NreC
**KCPMINPF_154384**	Copper-sensing transcriptional repressor CsoR
**KCPMINPF_154452**	Transcriptional regulatory protein FixJ
**KCPMINPF_15449**	Transcriptional regulatory protein WalR
**KCPMINPF_154635**	Formate hydrogenlyase transcriptional activator FhlA
**KCPMINPF_154810**	Transcriptional regulator SlyA
**KCPMINPF_15485**	Bifunctional ligase/repressor BirA
**KCPMINPF_154850**	Glucitol operon repressor
**KCPMINPF_154859**	Erythritol catabolism regulatory protein EryD
**KCPMINPF_155161**	Transcriptional regulatory protein BtsR
**KCPMINPF_15551**	Transcriptional regulatory protein SrrA
**KCPMINPF_155545**	Alkaline phosphatase synthesis transcriptional regulatory protein PhoP
**KCPMINPF_155589**	Transcriptional activator protein NhaR
**KCPMINPF_155637**	Iron-dependent repressor IdeR
**KCPMINPF_155794**	Transcriptional regulatory protein LiaR
**KCPMINPF_155797**	Transcriptional regulatory protein SrrA
**KCPMINPF_155933**	Sensory/regulatory protein RpfC
**KCPMINPF_155984**	Alkaline phosphatase synthesis transcriptional regulatory protein PhoP
**KCPMINPF_156098**	Heat-inducible transcription repressor HrcA
**KCPMINPF_156103**	Glycine cleavage system transcriptional activator
**KCPMINPF_15618**	LexA repressor
**KCPMINPF_156254**	Transcriptional regulatory protein QseB
**KCPMINPF_156270**	N-acetylglucosamine repressor
**KCPMINPF_15649**	Regulatory protein AtoC
**KCPMINPF_15652**	Transcriptional regulatory protein BtsR
**KCPMINPF_156563**	RNA polymerase-binding transcription factor DksA
**KCPMINPF_156752**	Oxygen regulatory protein NreC
**KCPMINPF_156768**	Transcriptional regulatory protein FixJ
**KCPMINPF_156902**	Transcriptional regulatory protein KdpE
**KCPMINPF_156955**	LexA repressor
**KCPMINPF_157052**	Transcriptional regulatory protein WalR
**KCPMINPF_157069**	Transcriptional repressor NrdR
**KCPMINPF_157140**	Transcriptional regulator SlyA
**KCPMINPF_157189**	Bifunctional ligase/repressor BirA
**KCPMINPF_15741**	RNA polymerase-binding transcription factor DksA
**KCPMINPF_157417**	Transcriptional regulatory protein LiaR
**KCPMINPF_15744**	Bifunctional transcriptional activator/DNA repair enzyme Ada
**KCPMINPF_157513**	Oxygen regulatory protein NreC

**KCPMINPF_157530**	DNA-binding transcriptional regulator NtrC
**KCPMINPF_15785**	Transcriptional regulatory protein ZraR
**KCPMINPF_157913**	Transcriptional regulator MraZ
**KCPMINPF_157957**	Transcriptional regulator MntR
**KCPMINPF_158082**	LexA repressor
**KCPMINPF_158109**	Nitrogen regulatory protein
**KCPMINPF_158183**	LexA repressor
**KCPMINPF_15838**	DNA-binding transcriptional regulator NtrC
**KCPMINPF_158479**	Pca regulon regulatory protein
**KCPMINPF_15884**	Transcriptional regulator MraZ
**KCPMINPF_158863**	Hydrogen peroxide-inducible genes activator
**KCPMINPF_159099**	LexA repressor
**KCPMINPF_159281**	Murein hydrolase activator NlpD
**KCPMINPF_159768**	Transcriptional regulatory protein YpdB
**KCPMINPF_160023**	Transcriptional regulatory protein QseB
**KCPMINPF_160131**	Copper-sensing transcriptional repressor CsoR
**KCPMINPF_16025**	Transcriptional regulator SlyA
**KCPMINPF_160321**	Transcriptional repressor NrdR
**KCPMINPF_160361**	Glucitol operon repressor
**KCPMINPF_160832**	Regulatory protein AtoC
**KCPMINPF_16084**	Transcriptional regulatory protein FixJ
**KCPMINPF_160857**	Transcriptional regulatory protein DegU
**KCPMINPF_160930**	Transcriptional regulatory protein DegU
**KCPMINPF_161389**	Anaerobic regulatory protein
**KCPMINPF_16144**	Regulatory protein AtoC
**KCPMINPF_161630**	Transcriptional regulatory protein QseB
**KCPMINPF_16166**	flagellum biosynthesis repressor protein FlbT
**KCPMINPF_16171**	Flagellar transcriptional regulator FtcR
**KCPMINPF_161755**	Oxygen regulatory protein NreC
**KCPMINPF_161834**	Oxygen regulatory protein NreC
**KCPMINPF_161885**	Oxygen regulatory protein NreC
**KCPMINPF_162049**	LexA repressor
**KCPMINPF_16214**	Transcriptional repressor SmtB
**KCPMINPF_162261**	Transcriptional regulatory protein DegU
**KCPMINPF_162332**	Alkaline phosphatase synthesis transcriptional regulatory protein PhoP
**KCPMINPF_162387**	Phosphate regulon transcriptional regulatory protein PhoB
**KCPMINPF_162389**	Transcriptional regulatory protein BtsR
**KCPMINPF_162579**	Alginate biosynthesis transcriptional regulatory protein AlgB
**KCPMINPF_16284**	Alkaline phosphatase synthesis transcriptional regulatory protein SphR
**KCPMINPF_162912**	Transcriptional regulator MraZ
**KCPMINPF_163010**	Regulatory protein AtoC
**KCPMINPF_163053**	Transcriptional regulatory protein WalR
**KCPMINPF_163085**	Transcriptional regulatory protein ros

**KCPMINPF_163146**	Photosynthetic apparatus regulatory protein RegA
**KCPMINPF_163177**	RNA polymerase-binding transcription factor DksA
**KCPMINPF_163211**	Mercuric resistance operon regulatory protein
**KCPMINPF_163346**	Transcriptional regulatory protein KdpE
**KCPMINPF_163466**	Transcriptional regulatory protein YpdB
**KCPMINPF_163725**	Transcriptional regulatory protein LnrK
**KCPMINPF_164123**	Nitrogen regulatory protein P-II 1
**KCPMINPF_164126**	Transcriptional regulatory protein OmpR
**KCPMINPF_164139**	Transcriptional regulatory protein ZraR
**KCPMINPF_164159**	Copper-sensing transcriptional repressor CsoR
**KCPMINPF_164306**	cAMP-activated global transcriptional regulator CRP
**KCPMINPF_164333**	DNA-binding transcriptional activator DecR
**KCPMINPF_164337**	Transcriptional regulatory protein CusR
**KCPMINPF_164377**	KDP operon transcriptional regulatory protein KdpE
**KCPMINPF_164400**	KDP operon transcriptional regulatory protein KdpE
**KCPMINPF_164618**	Transcriptional regulatory protein CusR
**KCPMINPF_164744**	DNA-binding transcriptional activator DevR/DosR
**KCPMINPF_164841**	Transcriptional regulator PerR
**KCPMINPF_165520**	LexA repressor
**KCPMINPF_165592**	LexA repressor
**KCPMINPF_165628**	Regulatory protein AtoC
**KCPMINPF_165872**	Transcriptional regulatory protein OmpR
**KCPMINPF_165904**	Nitrogen regulatory protein
**KCPMINPF_166015**	Flagellar transcriptional regulator FtcR
**KCPMINPF_16670**	flagellum biosynthesis repressor protein FlbT
**KCPMINPF_16678**	RNA polymerase-binding transcription factor DksA
**KCPMINPF_16768**	Transcriptional repressor PaaX
**KCPMINPF_16780**	Regulatory protein PchR
**KCPMINPF_17001**	Regulatory protein RecX
**KCPMINPF_17046**	Bifunctional ligase/repressor BirA
**KCPMINPF_17326**	Alkaline phosphatase synthesis transcriptional regulatory protein PhoP
**KCPMINPF_17429**	Transcriptional regulatory protein LiaR
**KCPMINPF_17503**	Bifunctional ligase/repressor BirA
**KCPMINPF_17644**	Transcriptional regulatory protein ZraR
**KCPMINPF_17658**	Regulatory protein AsnC
**KCPMINPF_17663**	Regulatory protein RecX
**KCPMINPF_17759**	Phosphate regulon transcriptional regulatory protein PhoB
**KCPMINPF_17847**	Nitrogen regulatory protein P-II
**KCPMINPF_17928**	Transcriptional regulatory protein BtsR
**KCPMINPF_18004**	Arsenic resistance transcriptional regulator ArsR2
**KCPMINPF_18212**	RNA polymerase-binding transcription factor DksA
**KCPMINPF_18326**	Transcriptional regulatory protein QseB
**KCPMINPF_18336**	Transcriptional regulatory protein CpxR

**KCPMINPF_18438**	Arginine repressor
**KCPMINPF_18446**	Transcriptional regulator MntR
**KCPMINPF_18520**	LexA repressor
**KCPMINPF_18556**	Bifunctional ligase/repressor BirA
**KCPMINPF_18617**	Transcriptional regulatory protein DegU
**KCPMINPF_18623**	RNA polymerase-binding transcription factor DksA
**KCPMINPF_18711**	Transcriptional repressor NrdR
**KCPMINPF_18725**	Leucine-responsive regulatory protein
**KCPMINPF_18970**	Transcriptional regulatory protein ZraR
**KCPMINPF_19003**	Transcriptional regulator MraZ
**KCPMINPF_19005**	Oxygen regulatory protein NreC
**KCPMINPF_19069**	Transcriptional regulatory protein ZraR
**KCPMINPF_19113**	LexA repressor
**KCPMINPF_19149**	Regulatory protein AtoC
**KCPMINPF_19167**	Transcriptional regulatory protein ZraR
**KCPMINPF_19183**	Transcriptional regulatory protein ZraR
**KCPMINPF_19254**	Nitrogen regulatory protein P-II 2
**KCPMINPF_19295**	Transcriptional regulatory protein QseB
**KCPMINPF_19364**	Transcriptional regulatory protein KdpE
**KCPMINPF_19376**	Nitrogen regulatory protein P-II 2
**KCPMINPF_19421**	KDP operon transcriptional regulatory protein KdpE
**KCPMINPF_19442**	Transcriptional regulatory protein tctD
**KCPMINPF_19588**	Fumarate and nitrate reduction regulatory protein
**KCPMINPF_19592**	Transcriptional regulatory protein BasR
**KCPMINPF_19600**	DnaA regulatory inactivator Hda
**KCPMINPF_19699**	Regulatory protein AtoC
**KCPMINPF_19910**	Heat-inducible transcription repressor HrcA
**KCPMINPF_19947**	Transcriptional regulator WhiB
**KCPMINPF_19962**	Transcriptional regulator WhiB
**KCPMINPF_20128**	LexA repressor
**KCPMINPF_20209**	Transcriptional repressor NrdR
**KCPMINPF_20269**	Formate hydrogenlyase transcriptional activator FhlA
**KCPMINPF_20497**	Octopine catabolism/uptake operon regulatory protein OccR
**KCPMINPF_20512**	Mannosyl-D-glycerate transport/metabolism system repressor MngR
**KCPMINPF_20568**	N-acetylglucosamine repressor
**KCPMINPF_20569**	Phosphate regulon transcriptional regulatory protein PhoB
**KCPMINPF_20582**	C4-dicarboxylate transport transcriptional regulatory protein DctD
**KCPMINPF_20605**	DNA-binding transcriptional regulator BolA
**KCPMINPF_20742**	Regulatory protein AtoC
**KCPMINPF_21072**	Transcriptional repressor NrdR
**KCPMINPF_21089**	Transcriptional regulator MraZ
**KCPMINPF_21156**	Transcriptional regulatory protein WalR
**KCPMINPF_21279**	Transcriptional repressor SmtB

**KCPMINPF_21282**	Transcriptional regulatory protein BaeR
**KCPMINPF_21348**	Transcriptional regulatory protein DegU
**KCPMINPF_21434**	Transcriptional regulatory protein LiaR
**KCPMINPF_21560**	Regulatory protein AtoC
**KCPMINPF_21568**	Transcriptional regulatory protein AfsQ1
**KCPMINPF_21572**	Oxygen regulatory protein NreC
**KCPMINPF_21636**	RNA polymerase-binding transcription factor DksA
**KCPMINPF_21815**	Regulatory protein AtoC
**KCPMINPF_21817**	RNA polymerase-binding transcription factor DksA
**KCPMINPF_21837**	Transcriptional regulatory protein CreB
**KCPMINPF_21934**	Aspartate carbamoyltransferase regulatory chain
**KCPMINPF_21974**	Regulatory protein AtoC
**KCPMINPF_22046**	Regulatory protein AtoC
**KCPMINPF_22131**	Glucitol operon repressor
**KCPMINPF_22202**	Ribose operon repressor
**KCPMINPF_22238**	Glucitol operon repressor
**KCPMINPF_22307**	LexA repressor
**KCPMINPF_22321**	Transcriptional regulator KdgR
**KCPMINPF_22353**	Transcriptional regulatory protein LiaR
**KCPMINPF_22573**	Transcriptional regulatory protein DegU
**KCPMINPF_22693**	Transcriptional regulatory protein WalR
**KCPMINPF_22729**	Transcriptional regulatory protein ZraR
**KCPMINPF_22733**	Regulatory protein AtoC
**KCPMINPF_22798**	Alkaline phosphatase synthesis transcriptional regulatory protein PhoP
**KCPMINPF_22886**	LexA repressor
**KCPMINPF_23065**	Alkaline phosphatase synthesis transcriptional regulatory protein PhoP
**KCPMINPF_23121**	Transcriptional regulatory protein LnrK
**KCPMINPF_23151**	p-hydroxybenzoate hydroxylase transcriptional activator
**KCPMINPF_23173**	Fumarate and nitrate reduction regulatory protein
**KCPMINPF_23272**	Transcriptional regulatory protein SrrA
**KCPMINPF_23635**	Zinc-specific metallo-regulatory protein
**KCPMINPF_23752**	Oxygen regulatory protein NreC
**KCPMINPF_23774**	Transcriptional regulatory protein YpdB
**KCPMINPF_23873**	Transcriptional regulatory protein ZraR
**KCPMINPF_23917**	Transcriptional regulatory protein BtsR
**KCPMINPF_24170**	Regulatory protein AtoC
**KCPMINPF_24222**	LexA repressor
**KCPMINPF_24243**	Alkaline phosphatase synthesis transcriptional regulatory protein PhoP
**KCPMINPF_24418**	DNA-binding transcriptional activator DecR
**KCPMINPF_24657**	Regulatory protein AtoC
**KCPMINPF_24709**	Transcriptional regulatory protein ZraR
**KCPMINPF_24827**	C4-dicarboxylate transport transcriptional regulatory protein DctD
**KCPMINPF_24907**	Regulatory protein AsnC

**KCPMINPF_25063**	Regulatory protein AtoC
**KCPMINPF_25086**	Alkaline phosphatase synthesis transcriptional regulatory protein PhoP
**KCPMINPF_25104**	Transcriptional regulatory protein DegU
**KCPMINPF_25133**	Redox-sensing transcriptional repressor Rex
**KCPMINPF_25381**	Transcriptional regulatory protein SrrA
**KCPMINPF_25476**	Phosphate regulon transcriptional regulatory protein PhoB
**KCPMINPF_25532**	Bifunctional ligase/repressor BirA
**KCPMINPF_25554**	Regulatory protein AtoC
**KCPMINPF_25555**	Regulatory protein AtoC
**KCPMINPF_25574**	Regulatory protein AtoC
**KCPMINPF_25628**	Heat-inducible transcription repressor HrcA
**KCPMINPF_25801**	Transcriptional regulatory protein OmpR
**KCPMINPF_25825**	Leucine-responsive regulatory protein
**KCPMINPF_25836**	Transcriptional regulator SlyA
**KCPMINPF_25992**	Redox-sensing transcriptional repressor Rex
**KCPMINPF_26221**	Arginine repressor
**KCPMINPF_26543**	Transcriptional activator protein CopR
**KCPMINPF_26745**	Diphtheria toxin repressor
**KCPMINPF_26773**	Transcriptional regulatory protein BtsR
**KCPMINPF_26895**	Transcriptional regulator WhiD
**KCPMINPF_26991**	Iron-dependent repressor IdeR
**KCPMINPF_26996**	Nitrogen regulatory protein P-II
**KCPMINPF_27010**	Transcriptional regulator WhiB
**KCPMINPF_27169**	Transcriptional regulatory protein OmpR
**KCPMINPF_27203**	Fumarate and nitrate reduction regulatory protein
**KCPMINPF_27447**	Alkaline phosphatase synthesis transcriptional regulatory protein PhoP
**KCPMINPF_27448**	Alkaline phosphatase synthesis transcriptional regulatory protein PhoP
**KCPMINPF_27480**	Alkaline phosphatase synthesis transcriptional regulatory protein PhoP
**KCPMINPF_27492**	Oxygen regulatory protein NreC
**KCPMINPF_27743**	Transcriptional regulatory protein PhoP
**KCPMINPF_27751**	Transcriptional regulatory protein CusR
**KCPMINPF_27907**	Bifunctional ligase/repressor BirA
**KCPMINPF_28236**	Nitrogen regulatory protein P-II
**KCPMINPF_28558**	Mannosyl-D-glycerate transport/metabolism system repressor MngR
**KCPMINPF_28624**	Transcriptional regulatory protein FixJ
**KCPMINPF_28672**	Hydrogen peroxide-inducible genes activator
**KCPMINPF_28714**	LexA repressor
**KCPMINPF_28832**	Transcriptional repressor IclR
**KCPMINPF_28851**	Transcriptional regulatory protein FixJ
**KCPMINPF_28863**	Transcriptional regulatory protein BtsR
**KCPMINPF_28874**	Nitrogen regulatory protein P-II
**KCPMINPF_29017**	Transcriptional regulatory protein ZraR
**KCPMINPF_29166**	Transcriptional regulatory protein LiaR

**KCPMINPF_29212**	Methanol dehydrogenase activator
**KCPMINPF_29330**	Penicillinase repressor
**KCPMINPF_29433**	Alkaline phosphatase synthesis transcriptional regulatory protein PhoP
**KCPMINPF_29688**	N-acetylglucosamine repressor
**KCPMINPF_29694**	N-acetylglucosamine repressor
**KCPMINPF_29784**	Hydrogen peroxide-inducible genes activator
**KCPMINPF_29790**	Glycerol-3-phosphate regulon repressor
**KCPMINPF_29836**	Pyruvate dehydrogenase complex repressor
**KCPMINPF_29840**	ATP phosphoribosyltransferase regulatory subunit
**KCPMINPF_29967**	Hydrogen peroxide-inducible genes activator
**KCPMINPF_30105**	LexA repressor
**KCPMINPF_30164**	Transcriptional regulatory protein KdpE
**KCPMINPF_30363**	Transcriptional regulator KdgR
**KCPMINPF_30384**	Transcriptional regulatory protein OmpR
**KCPMINPF_30427**	Leucine-responsive regulatory protein
**KCPMINPF_30519**	Transcriptional regulatory protein QseB
**KCPMINPF_30602**	Nitrogen regulatory protein P-II
**KCPMINPF_30631**	Regulatory protein AtoC
**KCPMINPF_30695**	Alkaline phosphatase synthesis transcriptional regulatory protein PhoP
**KCPMINPF_30838**	Transcriptional regulatory protein QseB
**KCPMINPF_30893**	Transcriptional regulator AcuR
**KCPMINPF_30958**	Transcriptional regulatory protein KdpE
**KCPMINPF_31039**	Transcriptional regulatory protein DegU
**KCPMINPF_31053**	Transcriptional regulator MraZ
**KCPMINPF_31108**	Bifunctional ligase/repressor BirA
**KCPMINPF_31246**	Transcriptional repressor NrdR
**KCPMINPF_31505**	Transcriptional regulatory protein DegU
**KCPMINPF_31626**	Transcriptional activatory protein BadR
**KCPMINPF_31686**	DNA-binding transcriptional activator DecR
**KCPMINPF_31731**	Transcriptional regulatory protein BaeR
**KCPMINPF_31832**	Transcriptional regulatory protein WalR
**KCPMINPF_31844**	Alkaline phosphatase synthesis transcriptional regulatory protein PhoP
**KCPMINPF_31922**	Transcriptional regulatory protein DegU
**KCPMINPF_31963**	Regulatory protein AtoC
**KCPMINPF_32046**	N-acetylglucosamine repressor
**KCPMINPF_32287**	Transcriptional regulatory protein CusR
**KCPMINPF_32291**	Alkaline phosphatase synthesis transcriptional regulatory protein PhoP
**KCPMINPF_32311**	Protein-arginine kinase activator protein
**KCPMINPF_32541**	Alkaline phosphatase synthesis transcriptional regulatory protein PhoP
**KCPMINPF_32690**	Transcriptional regulatory protein WalR
**KCPMINPF_32699**	Copper-sensing transcriptional repressor CsoR
**KCPMINPF_32758**	Transcriptional regulator WhiB
**KCPMINPF_32771**	Transcriptional regulatory protein DegU

**KCPMINPF_32777**	Hydrogen peroxide-inducible genes activator
**KCPMINPF_32886**	Transcriptional regulator SlyA
**KCPMINPF_32890**	Transcriptional regulatory protein BtsR
**KCPMINPF_32950**	Transcriptional regulatory protein DegU
**KCPMINPF_33034**	Heat-inducible transcription repressor HrcA
**KCPMINPF_33050**	Transcriptional repressor NrdR
**KCPMINPF_33057**	Regulatory protein AtoC
**KCPMINPF_33086**	Arginine repressor
**KCPMINPF_33202**	Regulatory protein AsnC
**KCPMINPF_33254**	Iron-dependent repressor IdeR
**KCPMINPF_33345**	Transcriptional regulator MraZ
**KCPMINPF_33556**	Fumarate and nitrate reduction regulatory protein
**KCPMINPF_33636**	Alkaline phosphatase synthesis transcriptional regulatory protein SphR
**KCPMINPF_33824**	Formate hydrogenlyase transcriptional activator FhlA
**KCPMINPF_33844**	Regulatory protein AtoC
**KCPMINPF_33847**	Regulatory protein AtoC
**KCPMINPF_33886**	Lactose operon repressor
**KCPMINPF_33899**	Transcriptional regulatory protein LiaR
**KCPMINPF_34023**	Heat-inducible transcription repressor HrcA
**KCPMINPF_34042**	Phosphoenolpyruvate synthase regulatory protein
**KCPMINPF_34054**	Glycine cleavage system transcriptional repressor
**KCPMINPF_34139**	DnaA regulatory inactivator Hda
**KCPMINPF_34414**	Bifunctional ligase/repressor BirA
**KCPMINPF_34513**	Murein hydrolase activator EnvC
**KCPMINPF_34534**	Regulatory protein AtoC
**KCPMINPF_34682**	LexA repressor
**KCPMINPF_34757**	LexA repressor
**KCPMINPF_34997**	Transcriptional activator protein Anr
**KCPMINPF_35009**	Leucine-responsive regulatory protein
**KCPMINPF_35030**	Transcriptional activator NphR
**KCPMINPF_35059**	Glycine cleavage system transcriptional activator
**KCPMINPF_35078**	Transcriptional regulatory protein QseB
**KCPMINPF_35238**	Methanol dehydrogenase activator
**KCPMINPF_35554**	Transcriptional regulatory protein LiaR
**KCPMINPF_35665**	Nitrogen regulatory protein P-II
**KCPMINPF_35763**	Transcriptional regulatory protein LiaR
**KCPMINPF_35852**	LexA repressor
**KCPMINPF_35901**	Heat-inducible transcription repressor HrcA
**KCPMINPF_35935**	Regulatory protein RecX
**KCPMINPF_35951**	Copper-sensing transcriptional repressor CsoR
**KCPMINPF_36159**	Leucine-responsive regulatory protein
**KCPMINPF_36201**	Biofilm growth-associated repressor
**KCPMINPF_36225**	Pca regulon regulatory protein

**KCPMINPF_36226**	Transcriptional regulator KdgR
**KCPMINPF_36374**	Transcriptional regulatory protein WalR
**KCPMINPF_36406**	LexA repressor
**KCPMINPF_36628**	Bifunctional transcriptional activator/DNA repair enzyme Ada
**KCPMINPF_36849**	Alkaline phosphatase synthesis transcriptional regulatory protein SphR
**KCPMINPF_36942**	DNA-binding transcriptional activator EvgA
**KCPMINPF_36979**	RNA polymerase-binding transcription factor DksA
**KCPMINPF_36984**	Hydrogen peroxide-inducible genes activator
**KCPMINPF_37021**	Regulatory protein AtoC
**KCPMINPF_37060**	PCP degradation transcriptional activation protein
**KCPMINPF_37168**	Alkaline phosphatase synthesis transcriptional regulatory protein PhoP
**KCPMINPF_37191**	Transcriptional regulatory protein OmpR
**KCPMINPF_37453**	Transcriptional regulatory protein TcrA
**KCPMINPF_37526**	Methanol dehydrogenase activator
**KCPMINPF_37612**	Regulatory protein AtoC
**KCPMINPF_37742**	Aspartate carbamoyltransferase regulatory chain
**KCPMINPF_37823**	Transcriptional regulatory protein LiaR
**KCPMINPF_38093**	Heat-inducible transcription repressor HrcA
**KCPMINPF_38173**	Transcriptional regulator MraZ
**KCPMINPF_38255**	Methanol dehydrogenase activator
**KCPMINPF_38287**	DNA-binding transcriptional regulator NtrC
**KCPMINPF_38520**	Transcriptional repressor IclR
**KCPMINPF_38522**	Transcriptional regulator KdgR
**KCPMINPF_38652**	Murein hydrolase activator EnvC
**KCPMINPF_38671**	KDP operon transcriptional regulatory protein KdpE
**KCPMINPF_38739**	Anaerobic regulatory protein
**KCPMINPF_38794**	Murein hydrolase activator EnvC
**KCPMINPF_38910**	Heat-inducible transcription repressor HrcA
**KCPMINPF_39079**	Heat-inducible transcription repressor HrcA
**KCPMINPF_39098**	Photosynthetic apparatus regulatory protein RegA
**KCPMINPF_39212**	Regulatory protein AtoC
**KCPMINPF_39333**	Regulatory protein AsnC
**KCPMINPF_39564**	Transcriptional regulator SlyA
**KCPMINPF_39726**	Transcriptional regulatory protein OmpR
**KCPMINPF_39828**	Transcriptional regulatory protein DegU
**KCPMINPF_39855**	Copper-sensing transcriptional repressor CsoR
**KCPMINPF_39872**	Transcriptional regulatory protein DegU
**KCPMINPF_39916**	Alkaline phosphatase synthesis transcriptional regulatory protein SphR
**KCPMINPF_39932**	Transcriptional repressor SmtB
**KCPMINPF_40000**	Regulatory protein RecX
**KCPMINPF_40096**	Transcriptional repressor NrdR
**KCPMINPF_40104**	Transcriptional regulator MraZ
**KCPMINPF_40136**	RNA polymerase-binding transcription factor CarD

**KCPMINPF_40203**	Leucine-responsive regulatory protein
**KCPMINPF_40415**	Transcriptional regulatory protein WalR
**KCPMINPF_40536**	Transcriptional repressor NrdR
**KCPMINPF_40563**	Alkaline phosphatase synthesis transcriptional regulatory protein PhoP
**KCPMINPF_40646**	Transcriptional regulatory protein DegU
**KCPMINPF_40845**	ATP phosphoribosyltransferase regulatory subunit
**KCPMINPF_40886**	Transcriptional regulatory protein WalR
**KCPMINPF_41011**	LexA repressor
**KCPMINPF_41015**	Regulatory protein AtoC
**KCPMINPF_41018**	Transcriptional regulatory protein LnrK
**KCPMINPF_41066**	Leucine-responsive regulatory protein
**KCPMINPF_41126**	LexA repressor
**KCPMINPF_41252**	Methanol dehydrogenase activator
**KCPMINPF_41338**	Transcriptional regulator LsrR
**KCPMINPF_41359**	Transcriptional regulatory protein AfsQ1
**KCPMINPF_41495**	Alkaline phosphatase synthesis transcriptional regulatory protein PhoP
**KCPMINPF_41507**	Transcriptional regulator SlyA
**KCPMINPF_41567**	Acetoin catabolism regulatory protein
**KCPMINPF_41594**	Alkaline phosphatase synthesis transcriptional regulatory protein SphR
**KCPMINPF_41654**	DNA-binding transcriptional activator DevR/DosR
**KCPMINPF_41655**	Transcriptional regulatory protein WalR
**KCPMINPF_41742**	Arginine repressor
**KCPMINPF_41798**	Murein hydrolase activator EnvC
**KCPMINPF_41982**	cAMP-activated global transcriptional regulator CRP
**KCPMINPF_42083**	Nitrogen regulatory protein P-II
**KCPMINPF_42111**	Bifunctional ligase/repressor BirA
**KCPMINPF_42141**	LexA repressor
**KCPMINPF_42340**	Biofilm regulatory protein A
**KCPMINPF_42342**	Transcriptional activator protein CopR
**KCPMINPF_42352**	Zinc-specific metallo-regulatory protein
**KCPMINPF_42412**	Regulatory protein AtoC
**KCPMINPF_42427**	Transcriptional regulator MraZ
**KCPMINPF_42508**	Regulatory protein AtoC
**KCPMINPF_42729**	Transcriptional regulatory protein KdpE
**KCPMINPF_42738**	Transcriptional regulatory protein WalR
**KCPMINPF_42849**	Regulatory protein AtoC
**KCPMINPF_42932**	Regulatory protein AsnC
**KCPMINPF_43013**	Transcriptional regulatory protein ZraR
**KCPMINPF_43385**	Transcriptional regulatory protein LiaR
**KCPMINPF_43401**	Alkaline phosphatase synthesis transcriptional regulatory protein PhoP
**KCPMINPF_43408**	Oxygen regulatory protein NreC
**KCPMINPF_43443**	Alkaline phosphatase synthesis transcriptional regulatory protein SphR
**KCPMINPF_43513**	Iron-dependent repressor IdeR

**KCPMINPF_43599**	Regulatory protein RecX
**KCPMINPF_43641**	Iron-dependent repressor IdeR
**KCPMINPF_43669**	N-acetylglucosamine repressor
**KCPMINPF_43730**	Transcriptional regulatory protein BaeR
**KCPMINPF_43747**	Transcriptional regulatory protein WalR
**KCPMINPF_43850**	Phosphate regulon transcriptional regulatory protein PhoB
**KCPMINPF_43868**	LexA repressor
**KCPMINPF_43896**	Transcriptional regulatory protein DegU
**KCPMINPF_43949**	Nitrogen regulatory protein
**KCPMINPF_43964**	Biofilm growth-associated repressor
**KCPMINPF_44024**	Anaerobic regulatory protein
**KCPMINPF_44306**	Alkaline phosphatase synthesis transcriptional regulatory protein SphR
**KCPMINPF_44355**	Copper-sensing transcriptional repressor CsoR
**KCPMINPF_44581**	Oxygen regulatory protein NreC
**KCPMINPF_44598**	Transcriptional regulatory protein DegU
**KCPMINPF_44665**	Transcriptional regulator SlyA
**KCPMINPF_44836**	Transcriptional regulatory protein LiaR
**KCPMINPF_44908**	DNA-binding transcriptional activator DevR/DosR
**KCPMINPF_44913**	Purine catabolism regulatory protein
**KCPMINPF_45017**	Bifunctional ligase/repressor BirA
**KCPMINPF_45308**	Copper-sensing transcriptional repressor CsoR
**KCPMINPF_45498**	Bifunctional ligase/repressor BirA
**KCPMINPF_45575**	Regulatory protein AtoC
**KCPMINPF_45946**	Heat-inducible transcription repressor HrcA
**KCPMINPF_45993**	Phosphate regulon transcriptional regulatory protein PhoB
**KCPMINPF_46066**	Leucine-responsive regulatory protein
**KCPMINPF_46070**	RNA polymerase-binding transcription factor DksA
**KCPMINPF_46141**	Transcriptional regulatory protein tctD
**KCPMINPF_46376**	Transcriptional regulatory protein FixJ
**KCPMINPF_46517**	Anaerobic regulatory protein
**KCPMINPF_46591**	Bifunctional ligase/repressor BirA
**KCPMINPF_46631**	Transcriptional regulator PerR
**KCPMINPF_46953**	Lactose operon repressor
**KCPMINPF_46974**	Glycerol-3-phosphate regulon repressor
**KCPMINPF_47012**	Regulatory protein AtoC
**KCPMINPF_47035**	Regulatory protein AtoC
**KCPMINPF_47219**	Hydrogen peroxide-inducible genes activator
**KCPMINPF_47223**	Glycine cleavage system transcriptional activator
**KCPMINPF_47241**	Transcriptional activator HlyU
**KCPMINPF_47292**	Transcriptional regulatory protein WalR
**KCPMINPF_47295**	Transcriptional regulatory protein QseB
**KCPMINPF_47307**	Transcriptional regulator SlyA
**KCPMINPF_47311**	Transcriptional regulatory protein DegU

**KCPMINPF_47465**	Transcriptional regulator MraZ
**KCPMINPF_47492**	Alkaline phosphatase synthesis transcriptional regulatory protein PhoP
**KCPMINPF_47598**	Copper-sensing transcriptional repressor CsoR
**KCPMINPF_47802**	Phosphate regulon transcriptional regulatory protein PhoB
**KCPMINPF_47803**	DNA-binding transcriptional activator DevR/DosR
**KCPMINPF_47865**	Transcriptional regulatory protein LnrK
**KCPMINPF_47954**	cAMP-activated global transcriptional regulator CRP
**KCPMINPF_47958**	Nitrogen regulatory protein P-II
**KCPMINPF_47992**	Transcriptional activator protein Anr
**KCPMINPF_48156**	Transcriptional regulatory protein DegU
**KCPMINPF_48199**	Regulatory protein MsrR
**KCPMINPF_48252**	Glycerol-3-phosphate regulon repressor
**KCPMINPF_48340**	KDP operon transcriptional regulatory protein KdpE
**KCPMINPF_48377**	Nitrogen regulatory protein P-II
**KCPMINPF_48423**	N-acetylglucosamine repressor
**KCPMINPF_48452**	Transcriptional regulator MraZ
**KCPMINPF_48548**	Transcriptional regulatory protein WalR
**KCPMINPF_48570**	Alkaline phosphatase synthesis transcriptional regulatory protein PhoP
**KCPMINPF_48651**	Alkaline phosphatase synthesis transcriptional regulatory protein PhoP
**KCPMINPF_48683**	Regulatory protein AtoC
**KCPMINPF_48704**	Transcriptional regulatory protein ZraR
**KCPMINPF_48716**	Transcriptional regulatory protein PmpR
**KCPMINPF_48778**	LexA repressor
**KCPMINPF_48887**	Bifunctional ligase/repressor BirA
**KCPMINPF_49066**	Regulatory protein AtoC
**KCPMINPF_49127**	Transcriptional repressor NrdR
**KCPMINPF_49139**	Regulatory protein AtoC
**KCPMINPF_49164**	Regulatory protein RecX
**KCPMINPF_49460**	Regulatory protein AtoC
**KCPMINPF_49467**	Alkaline phosphatase synthesis transcriptional regulatory protein PhoP
**KCPMINPF_49607**	Transcriptional regulator LsrR
**KCPMINPF_49661**	RNA polymerase-binding transcription factor DksA
**KCPMINPF_49689**	DNA-binding transcriptional regulator NtrC
**KCPMINPF_49694**	Alkaline phosphatase synthesis transcriptional regulatory protein PhoP
**KCPMINPF_49706**	Redox-sensing transcriptional repressor Rex
**KCPMINPF_49770**	Transcriptional regulatory protein KdpE
**KCPMINPF_49792**	Transcriptional regulatory protein QseB
**KCPMINPF_49826**	DNA-binding transcriptional activator DecR
**KCPMINPF_49858**	Transcriptional activator HlyU
**KCPMINPF_50095**	Glucitol operon repressor
**KCPMINPF_50101**	Transcriptional regulator ManR
**KCPMINPF_50202**	Transcriptional repressor NrdR
**KCPMINPF_50337**	RNA polymerase-binding transcription factor DksA

**KCPMINPF_50509**	Transcriptional repressor SmtB
**KCPMINPF_50524**	Transcriptional regulatory protein LiaR
**KCPMINPF_50535**	Regulatory protein AtoC
**KCPMINPF_50537**	Oxygen regulatory protein NreC
**KCPMINPF_50587**	Glucitol operon repressor
**KCPMINPF_50675**	Alkaline phosphatase synthesis transcriptional regulatory protein PhoP
**KCPMINPF_50785**	RNA polymerase-binding transcription factor DksA
**KCPMINPF_50815**	Transcriptional regulatory protein BtsR
**KCPMINPF_50836**	Nif-specific regulatory protein
**KCPMINPF_51012**	Transcriptional regulatory protein WalR
**KCPMINPF_51215**	Transcriptional regulator SlyA
**KCPMINPF_51230**	RNA polymerase-binding transcription factor DksA
**KCPMINPF_51249**	Octopine catabolism/uptake operon regulatory protein OccR
**KCPMINPF_51259**	Transcriptional regulator HilA
**KCPMINPF_51284**	(R)-phenyllactate dehydratase activator
**KCPMINPF_51285**	(R)-phenyllactate dehydratase activator
**KCPMINPF_51500**	Transcriptional regulatory protein LiaR
**KCPMINPF_51631**	Iron-dependent repressor IdeR
**KCPMINPF_51926**	Alkaline phosphatase synthesis transcriptional regulatory protein PhoP
**KCPMINPF_52055**	Transcriptional regulatory protein CusR
**KCPMINPF_52185**	Transcriptional regulatory protein LiaR
**KCPMINPF_52205**	Bifunctional transcriptional activator/DNA repair enzyme Ada
**KCPMINPF_52240**	Redox-sensing transcriptional repressor Rex
**KCPMINPF_52245**	Redox-sensing transcriptional repressor Rex
**KCPMINPF_52268**	Bifunctional ligase/repressor BirA
**KCPMINPF_52299**	Oxygen regulatory protein NreC
**KCPMINPF_52408**	Transcriptional regulator MraZ
**KCPMINPF_52683**	Acetoin catabolism regulatory protein
**KCPMINPF_52734**	RNA polymerase-binding transcription factor DksA
**KCPMINPF_52764**	Transcriptional regulator MraZ
**KCPMINPF_52838**	Regulatory protein AtoC
**KCPMINPF_52848**	Heat-inducible transcription repressor HrcA
**KCPMINPF_52898**	Regulatory protein AtoC
**KCPMINPF_52908**	Heat-inducible transcription repressor HrcA
**KCPMINPF_53053**	PCP degradation transcriptional activation protein
**KCPMINPF_53068**	Transcriptional regulatory protein WalR
**KCPMINPF_53076**	Nitrogen regulatory protein P-II 2
**KCPMINPF_53078**	Nitrogen regulatory protein P-II 2
**KCPMINPF_53172**	Transcriptional regulatory protein QseF
**KCPMINPF_53190**	Penicillin-binding protein activator LpoB
**KCPMINPF_53256**	Transcriptional regulator WhiB2
**KCPMINPF_53263**	Transcriptional regulator WhiB
**KCPMINPF_53403**	Regulatory protein AtoC

**KCPMINPF_53461**	Transcriptional regulatory protein DegU
**KCPMINPF_53726**	Photosynthetic apparatus regulatory protein RegA
**KCPMINPF_53751**	Transcriptional regulatory protein DegU
**KCPMINPF_54076**	RNA polymerase-binding transcription factor DksA
**KCPMINPF_54103**	Phosphoenolpyruvate synthase regulatory protein
**KCPMINPF_54189**	2-hydroxyisocaproyl-CoA dehydratase activator
**KCPMINPF_54190**	2-hydroxyisocaproyl-CoA dehydratase activator
**KCPMINPF_54195**	Transcriptional repressor PaaX
**KCPMINPF_54450**	Transcriptional regulatory protein BtsR
**KCPMINPF_54522**	DNA-binding transcriptional regulator NtrC
**KCPMINPF_54556**	Regulatory protein RecX
**KCPMINPF_54579**	Transcriptional regulator MraZ
**KCPMINPF_54887**	Transcriptional regulatory protein LiaR
**KCPMINPF_54900**	ATP phosphoribosyltransferase regulatory subunit
**KCPMINPF_54941**	Transcriptional regulatory protein LiaR
**KCPMINPF_55043**	Transcriptional regulatory protein WalR
**KCPMINPF_55096**	Alkaline phosphatase synthesis transcriptional regulatory protein SphR
**KCPMINPF_55191**	Glycerol operon regulatory protein
**KCPMINPF_55311**	Transcriptional regulatory protein LiaR
**KCPMINPF_55327**	Transcriptional regulatory protein DegU
**KCPMINPF_55361**	Iron-dependent repressor IdeR
**KCPMINPF_55375**	Alkaline phosphatase synthesis transcriptional regulatory protein PhoP
**KCPMINPF_55378**	Transcriptional repressor SmtB
**KCPMINPF_55400**	Nitrogen regulatory protein P-II
**KCPMINPF_55410**	Bifunctional ligase/repressor BirA
**KCPMINPF_55441**	Glycine cleavage system transcriptional activator
**KCPMINPF_55448**	DNA-binding transcriptional regulator NtrC
**KCPMINPF_55450**	DNA-binding transcriptional regulator NtrC
**KCPMINPF_55527**	Leucine-responsive regulatory protein
**KCPMINPF_55596**	Photosynthetic apparatus regulatory protein RegA
**KCPMINPF_55630**	Pca regulon regulatory protein
**KCPMINPF_55708**	Leucine-responsive regulatory protein
**KCPMINPF_55812**	Transcriptional regulator PerR
**KCPMINPF_56062**	Heat-inducible transcription repressor HrcA
**KCPMINPF_56146**	Alkaline phosphatase synthesis transcriptional regulatory protein PhoP
**KCPMINPF_56201**	RNA polymerase-binding transcription factor DksA
**KCPMINPF_56372**	Hca operon transcriptional activator HcaR
**KCPMINPF_56582**	Transcriptional regulatory protein AfsQ1
**KCPMINPF_56685**	Hydrogen peroxide-inducible genes activator
**KCPMINPF_56788**	Transcriptional regulatory protein KdpE
**KCPMINPF_56891**	Copper-sensing transcriptional repressor CsoR
**KCPMINPF_56910**	RNA polymerase-binding transcription factor DksA
**KCPMINPF_56913**	Phosphate regulon transcriptional regulatory protein PhoB

**KCPMINPF_57016**	Transcriptional regulatory protein BaeR
**KCPMINPF_57058**	Alkaline phosphatase synthesis transcriptional regulatory protein PhoP
**KCPMINPF_57063**	Alkaline phosphatase synthesis transcriptional regulatory protein PhoP
**KCPMINPF_57095**	Regulatory protein RecX
**KCPMINPF_57157**	Transcriptional regulatory protein LnrK
**KCPMINPF_57165**	Iron-dependent repressor IdeR
**KCPMINPF_57247**	Penicillin-binding protein activator LpoA
**KCPMINPF_57252**	Heat-inducible transcription repressor HrcA
**KCPMINPF_57302**	ATP phosphoribosyltransferase regulatory subunit
**KCPMINPF_57375**	Regulatory protein AtoC
**KCPMINPF_57454**	Transcriptional regulator MraZ
**KCPMINPF_57540**	Nitrogen regulatory protein P-II 2
**KCPMINPF_57643**	Transcriptional regulatory protein TcrA
**KCPMINPF_57648**	Transcriptional regulatory protein QseB
**KCPMINPF_57688**	Transcriptional regulatory protein SrrA
**KCPMINPF_57725**	Oxygen regulatory protein NreC
**KCPMINPF_57910**	Transcriptional repressor NrdR
**KCPMINPF_58026**	Transcriptional regulatory protein QseB
**KCPMINPF_58180**	N-acetylglucosamine repressor
**KCPMINPF_58313**	Transcriptional regulatory protein LiaR
**KCPMINPF_58380**	Alkaline phosphatase synthesis transcriptional regulatory protein PhoP
**KCPMINPF_58469**	Regulatory protein RecX
**KCPMINPF_58497**	DNA-binding transcriptional activator DecR
**KCPMINPF_58509**	Transcriptional regulator SlyA
**KCPMINPF_58603**	Phosphate regulon transcriptional regulatory protein PhoB
**KCPMINPF_58692**	Alkaline phosphatase synthesis transcriptional regulatory protein PhoP
**KCPMINPF_58828**	Transcriptional regulatory protein BtsR
**KCPMINPF_58868**	Denitrification regulatory protein NirQ
**KCPMINPF_58941**	Transcriptional regulatory protein AfsQ1
**KCPMINPF_59133**	Transcriptional regulatory protein DegU
**KCPMINPF_59283**	Regulatory protein RecX
**KCPMINPF_59286**	Transcriptional regulator MraZ
**KCPMINPF_59303**	Transcriptional repressor NrdR
**KCPMINPF_59349**	Transcriptional regulatory protein TcrA
**KCPMINPF_59519**	Transcriptional regulatory protein DegU
**KCPMINPF_59556**	Murein hydrolase activator EnvC
**KCPMINPF_59588**	Bifunctional ligase/repressor BirA
**KCPMINPF_59678**	N-acetylglucosamine repressor
**KCPMINPF_59981**	Iron-dependent repressor IdeR
**KCPMINPF_60032**	Transcriptional regulatory protein LnrK
**KCPMINPF_60061**	Transcriptional regulatory protein DesR
**KCPMINPF_60194**	Transcriptional repressor IclR
**KCPMINPF_60312**	RNA polymerase-binding transcription factor CarD

**KCPMINPF_60460**	Transcriptional regulator KdgR
**KCPMINPF_60468**	DNA-binding transcriptional regulator BolA
**KCPMINPF_60737**	Nitrogen regulatory protein
**KCPMINPF_60972**	Transcriptional regulatory protein WalR
**KCPMINPF_61337**	Heat-inducible transcription repressor HrcA
**KCPMINPF_61481**	Alkaline phosphatase synthesis transcriptional regulatory protein PhoP
**KCPMINPF_61691**	Transcriptional regulatory protein QseB
**KCPMINPF_61821**	Mercuric resistance operon regulatory protein
**KCPMINPF_61828**	Transcriptional activator protein CopR
**KCPMINPF_61836**	Transcriptional activator protein CopR
**KCPMINPF_61846**	Transcriptional regulatory protein LnrK
**KCPMINPF_61914**	RNA polymerase-binding transcription factor DksA
**KCPMINPF_62036**	Transcriptional regulatory protein WalR
**KCPMINPF_62088**	Transcriptional regulatory protein BtsR
**KCPMINPF_62127**	Murein hydrolase activator NlpD
**KCPMINPF_62229**	Arabinose metabolism transcriptional repressor
**KCPMINPF_62279**	Regulatory protein RecX
**KCPMINPF_62293**	Transcriptional regulatory protein WalR
**KCPMINPF_62370**	Transcriptional regulator PerR
**KCPMINPF_62424**	Transcriptional regulatory protein OmpR
**KCPMINPF_62471**	Transcriptional regulatory protein CreB
**KCPMINPF_62566**	Transcriptional regulatory protein ZraR
**KCPMINPF_62682**	Transcriptional regulatory protein PhoP
**KCPMINPF_62691**	Transcriptional regulatory protein CusR
**KCPMINPF_63293**	LexA repressor
**KCPMINPF_63427**	Transcriptional regulatory protein SrrA
**KCPMINPF_63558**	CdaA regulatory protein CdaR
**KCPMINPF_63626**	Transcriptional regulatory protein KdpE
**KCPMINPF_63648**	Regulatory protein AtoC
**KCPMINPF_63844**	Anaerobic regulatory protein
**KCPMINPF_63902**	Phosphate regulon transcriptional regulatory protein PhoB
**KCPMINPF_63950**	Bifunctional ligase/repressor BirA
**KCPMINPF_63968**	Transcriptional regulatory protein WalR
**KCPMINPF_64150**	Glycerol-3-phosphate regulon repressor
**KCPMINPF_64202**	Transcriptional regulatory protein LiaR
**KCPMINPF_64248**	Hca operon transcriptional activator HcaR
**KCPMINPF_64466**	KDP operon transcriptional regulatory protein KdpE
**KCPMINPF_64472**	Iron-dependent repressor IdeR
**KCPMINPF_64654**	Transcriptional regulatory protein ros
**KCPMINPF_64714**	Denitrification regulatory protein NirQ
**KCPMINPF_64778**	Nitrogen regulatory protein P-II
**KCPMINPF_64892**	N-acetylglucosamine repressor
**KCPMINPF_64916**	Transcriptional repressor SmtB

**KCPMINPF_64960**	Alkaline phosphatase synthesis transcriptional regulatory protein PhoP
**KCPMINPF_64999**	PTS-dependent dihydroxyacetone kinase operon regulatory protein
**KCPMINPF_65021**	Alkaline phosphatase synthesis transcriptional regulatory protein SphR
**KCPMINPF_65104**	Transcriptional regulatory protein DegU
**KCPMINPF_65242**	Transcriptional regulatory protein LiaR
**KCPMINPF_65263**	Transcriptional regulator MntR
**KCPMINPF_65361**	Hydrogen peroxide-inducible genes activator
**KCPMINPF_65553**	Transcriptional regulatory protein DegU
**KCPMINPF_65557**	Transcriptional regulatory protein QseB
**KCPMINPF_65792**	Sensory/regulatory protein RpfC
**KCPMINPF_65796**	Transcriptional repressor NrdR
**KCPMINPF_65828**	Bifunctional ligase/repressor BirA
**KCPMINPF_65869**	Alkaline phosphatase synthesis transcriptional regulatory protein PhoP
**KCPMINPF_66129**	LexA repressor
**KCPMINPF_66196**	Pyruvate dehydrogenase complex repressor
**KCPMINPF_66388**	N-acetylglucosamine repressor
**KCPMINPF_66583**	Transcriptional regulatory protein WalR
**KCPMINPF_66620**	Bifunctional ligase/repressor BirA
**KCPMINPF_66638**	Transcriptional activator NphR
**KCPMINPF_66645**	Penicillin-binding protein activator LpoA
**KCPMINPF_66675**	LexA repressor
**KCPMINPF_66683**	Extracellular matrix regulatory protein A
**KCPMINPF_66794**	Transcriptional regulatory protein DesR
**KCPMINPF_67028**	Transcriptional regulatory protein KdpE
**KCPMINPF_67156**	DNA-binding transcriptional activator DevR/DosR
**KCPMINPF_67161**	Transcriptional regulatory protein WalR
**KCPMINPF_67253**	Glucitol operon repressor
**KCPMINPF_67301**	Transcriptional regulator SdrP
**KCPMINPF_67381**	2-hydroxyisocaproyl-CoA dehydratase activator
**KCPMINPF_67382**	(R)-phenyllactate dehydratase activator
**KCPMINPF_67417**	Sigma factor AlgU regulatory protein MucB
**KCPMINPF_67536**	Transcriptional regulatory protein FixJ
**KCPMINPF_67774**	Transcriptional repressor NrdR
**KCPMINPF_67786**	Transcriptional regulatory protein LnrK
**KCPMINPF_67881**	Negative regulatory protein YxlE
**KCPMINPF_67972**	Oxygen regulatory protein NreC
**KCPMINPF_68032**	Alkaline phosphatase synthesis transcriptional regulatory protein PhoP
**KCPMINPF_68116**	Transcriptional regulatory protein DegU
**KCPMINPF_68273**	Regulatory protein AtoC
**KCPMINPF_68768**	Transcriptional regulatory protein WalR
**KCPMINPF_68788**	Alkaline phosphatase synthesis transcriptional regulatory protein PhoP
**KCPMINPF_68820**	Transcriptional repressor NrdR
**KCPMINPF_68942**	Ribose operon repressor

**KCPMINPF_69051**	Transcriptional regulatory protein LiaR
**KCPMINPF_69117**	Alkaline phosphatase synthesis transcriptional regulatory protein PhoP
**KCPMINPF_69210**	Transcriptional regulatory protein OmpR
**KCPMINPF_69293**	Regulatory protein RecX
**KCPMINPF_69553**	Glucitol operon repressor
**KCPMINPF_69647**	Transcriptional regulatory protein LnrK
**KCPMINPF_69779**	Alkaline phosphatase synthesis transcriptional regulatory protein PhoP
**KCPMINPF_69799**	Transcriptional activator protein CzcR
**KCPMINPF_69935**	Glycerol-3-phosphate regulon repressor
**KCPMINPF_70160**	Arginine repressor
**KCPMINPF_70217**	LexA repressor
**KCPMINPF_70240**	Alkaline phosphatase synthesis transcriptional regulatory protein PhoP
**KCPMINPF_70489**	RNA polymerase-binding transcription factor DksA
**KCPMINPF_70562**	Transcriptional regulatory protein ZraR
**KCPMINPF_70625**	Bifunctional ligase/repressor BirA
**KCPMINPF_70627**	Heat-inducible transcription repressor HrcA
**KCPMINPF_70645**	Leucine-responsive regulatory protein
**KCPMINPF_70842**	Transcriptional regulatory protein WalR
**KCPMINPF_70916**	Hydrogen peroxide-inducible genes activator
**KCPMINPF_71019**	Regulatory protein AtoC
**KCPMINPF_71117**	LexA repressor
**KCPMINPF_71143**	Transcriptional regulatory protein KdpE
**KCPMINPF_71232**	Nitrogen regulatory protein P-II
**KCPMINPF_71332**	(R)-phenyllactate dehydratase activator
**KCPMINPF_71333**	(R)-phenyllactate dehydratase activator
**KCPMINPF_71469**	Leucine-responsive regulatory protein
**KCPMINPF_71546**	Transcriptional regulator MraZ
**KCPMINPF_71581**	CdaA regulatory protein CdaR
**KCPMINPF_71694**	RNA polymerase-binding transcription factor DksA
**KCPMINPF_71710**	Redox-sensing transcriptional repressor Rex
**KCPMINPF_71754**	Regulatory protein AfsR
**KCPMINPF_71856**	Oxygen regulatory protein NreC
**KCPMINPF_71946**	Transcriptional regulatory protein DegU
**KCPMINPF_71970**	Bifunctional transcriptional activator/DNA repair enzyme Ada
**KCPMINPF_72018**	Alkaline phosphatase synthesis transcriptional regulatory protein PhoP
**KCPMINPF_72243**	Transcriptional regulatory protein CpxR
**KCPMINPF_72391**	Transcriptional regulatory protein DegU
**KCPMINPF_72405**	Heat-inducible transcription repressor HrcA
**KCPMINPF_72596**	Transcriptional regulatory protein TcrA
**KCPMINPF_72614**	Zinc-specific metallo-regulatory protein
**KCPMINPF_72746**	N-acetylglucosamine repressor
**KCPMINPF_72829**	Transcriptional regulatory protein LiaR
**KCPMINPF_72871**	Redox-sensing transcriptional repressor Rex

**KCPMINPF_72888**	Transcriptional regulatory protein DegU
**KCPMINPF_72909**	Transcriptional regulatory protein TcrA
**KCPMINPF_73033**	Transcriptional activator protein CopR
**KCPMINPF_73136**	RNA polymerase-binding transcription factor DksA
**KCPMINPF_73429**	Arsenic resistance transcriptional regulator ArsR1
**KCPMINPF_73591**	Bifunctional ligase/repressor BirA
**KCPMINPF_73671**	Transcriptional regulator MraZ
**KCPMINPF_73692**	Transcriptional repressor NrdR
**KCPMINPF_73801**	Transcriptional regulatory protein DegU
**KCPMINPF_73862**	Acetoin catabolism regulatory protein
**KCPMINPF_73939**	Ribose operon repressor
**KCPMINPF_73979**	Transcriptional regulatory protein OmpR
**KCPMINPF_74023**	Transcriptional regulatory protein TdiR
**KCPMINPF_74133**	Redox-sensing transcriptional repressor Rex 1
**KCPMINPF_74154**	Transcriptional regulatory protein DegU
**KCPMINPF_74155**	Transcriptional regulatory protein LnrK
**KCPMINPF_74190**	LexA repressor
**KCPMINPF_74216**	Transcriptional regulatory protein DegU
**KCPMINPF_74234**	Transcriptional regulator SdrP
**KCPMINPF_74242**	RNA polymerase-binding transcription factor CarD
**KCPMINPF_74286**	DnaA regulatory inactivator Hda
**KCPMINPF_74312**	Transcriptional regulatory protein DegU
**KCPMINPF_74348**	Transcriptional regulatory protein WalR
**KCPMINPF_74440**	Regulatory protein AtoC
**KCPMINPF_74559**	Heat-inducible transcription repressor HrcA
**KCPMINPF_74600**	Hydrogen peroxide-inducible genes activator
**KCPMINPF_74674**	Transcriptional repressor NrdR
**KCPMINPF_74805**	Transcriptional regulatory protein AfsQ1
**KCPMINPF_74827**	Bifunctional ligase/repressor BirA
**KCPMINPF_74829**	DNA-binding transcriptional regulator NtrC
**KCPMINPF_74863**	DNA-binding transcriptional activator DevR/DosR
**KCPMINPF_74986**	Regulatory protein AtoC
**KCPMINPF_75097**	Diphtheria toxin repressor
**KCPMINPF_75233**	Transcriptional regulator MraZ
**KCPMINPF_75347**	Transcriptional regulatory protein WalR
**KCPMINPF_75529**	Transcriptional regulatory protein DegU
**KCPMINPF_75559**	Copper-sensing transcriptional repressor CsoR
**KCPMINPF_75630**	Heat-inducible transcription repressor HrcA
**KCPMINPF_75669**	Transcriptional regulatory protein KdpE
**KCPMINPF_75693**	Bifunctional transcriptional activator/DNA repair enzyme Ada
**KCPMINPF_75783**	Transcriptional regulatory protein LiaR
**KCPMINPF_75806**	Transcriptional regulatory protein QseB
**KCPMINPF_75842**	Transcriptional regulatory protein WalR

**KCPMINPF_75992**	Nitrogen regulatory protein P-II 2
**KCPMINPF_76032**	Transcriptional regulatory protein QseF
**KCPMINPF_76367**	Alkaline phosphatase synthesis transcriptional regulatory protein PhoP
**KCPMINPF_76377**	Alkaline phosphatase synthesis transcriptional regulatory protein SphR
**KCPMINPF_76405**	Transcriptional regulator MraZ
**KCPMINPF_76476**	LexA repressor
**KCPMINPF_76531**	KDP operon transcriptional regulatory protein KdpE
**KCPMINPF_76624**	Transcriptional regulatory protein PmpR
**KCPMINPF_76745**	Bifunctional ligase/repressor BirA
**KCPMINPF_76784**	Hydrogenase transcriptional regulatory protein hupR1
**KCPMINPF_76936**	Transcriptional regulatory protein LiaR
**KCPMINPF_77006**	Transcriptional regulatory protein WalR
**KCPMINPF_77152**	LexA repressor
**KCPMINPF_77166**	Nitrogen regulatory protein P-II 2
**KCPMINPF_77289**	flagellum biosynthesis repressor protein FlbT
**KCPMINPF_77343**	ATP phosphoribosyltransferase regulatory subunit
**KCPMINPF_77813**	Transcriptional regulatory protein QseB
**KCPMINPF_77936**	Nitrogen regulatory protein
**KCPMINPF_78096**	Iron-dependent repressor IdeR
**KCPMINPF_78221**	Alkaline phosphatase synthesis transcriptional regulatory protein PhoP
**KCPMINPF_78253**	Alkaline phosphatase synthesis transcriptional regulatory protein PhoP
**KCPMINPF_78290**	Redox-sensing transcriptional repressor Rex 1
**KCPMINPF_78578**	Regulatory protein AtoC
**KCPMINPF_78614**	Transcriptional regulatory protein DegU
**KCPMINPF_78721**	Transcriptional regulatory protein DegU
**KCPMINPF_78739**	Leucine-responsive regulatory protein
**KCPMINPF_78793**	Transcriptional regulatory protein KdpE
**KCPMINPF_78883**	Transcriptional regulatory protein KdpE
**KCPMINPF_79061**	N-acetylglucosamine repressor
**KCPMINPF_79069**	Transcriptional regulatory protein DegU
**KCPMINPF_79184**	Transcriptional regulatory protein LiaR
**KCPMINPF_79333**	Oxygen regulatory protein NreC
**KCPMINPF_79378**	Bifunctional ligase/repressor BirA
**KCPMINPF_79506**	N-acetylglucosamine repressor
**KCPMINPF_79511**	N-acetylglucosamine repressor
**KCPMINPF_79703**	Luminescence regulatory protein LuxO
**KCPMINPF_79799**	Oxygen regulatory protein NreC
**KCPMINPF_79809**	Ribose operon repressor
**KCPMINPF_79975**	Regulatory protein RecX
**KCPMINPF_80045**	Transcriptional regulatory protein LiaR
**KCPMINPF_80130**	ATP phosphoribosyltransferase regulatory subunit
**KCPMINPF_80155**	Glycine cleavage system transcriptional activator
**KCPMINPF_80321**	LexA repressor

**KCPMINPF_80547**	Transcriptional regulatory protein LiaR
**KCPMINPF_80628**	Alkaline phosphatase synthesis transcriptional regulatory protein PhoP
**KCPMINPF_80669**	Phosphate regulon transcriptional regulatory protein PhoB
**KCPMINPF_80686**	Transcriptional regulatory protein WalR
**KCPMINPF_80922**	Transcriptional regulatory protein DegU
**KCPMINPF_80968**	cAMP-activated global transcriptional regulator CRP
**KCPMINPF_81053**	Transcriptional regulatory protein DesR
**KCPMINPF_81072**	RNA polymerase-binding transcription factor CarD
**KCPMINPF_81073**	Transcriptional regulatory protein DesR
**KCPMINPF_81119**	Transcriptional regulatory protein KdpE
**KCPMINPF_81131**	Regulatory protein AtoC
**KCPMINPF_81149**	RNA polymerase-binding transcription factor DksA
**KCPMINPF_81169**	Glucitol operon repressor
**KCPMINPF_81250**	Regulatory protein RecX
**KCPMINPF_81254**	Nitrogen regulatory protein
**KCPMINPF_81373**	Mercuric resistance operon regulatory protein
**KCPMINPF_81522**	Psp operon transcriptional activator
**KCPMINPF_81560**	Alkaline phosphatase synthesis transcriptional regulatory protein PhoP
**KCPMINPF_81561**	DNA-binding transcriptional activator DevR/DosR
**KCPMINPF_81781**	Transcriptional activator protein CopR
**KCPMINPF_81845**	Protein-arginine kinase activator protein
**KCPMINPF_81884**	Lactose operon repressor
**KCPMINPF_82008**	Heat-inducible transcription repressor HrcA
**KCPMINPF_82052**	Oxygen regulatory protein NreC
**KCPMINPF_82117**	Transcriptional regulatory protein LiaR
**KCPMINPF_82136**	Lactose operon repressor
**KCPMINPF_82176**	Transcriptional regulatory protein DegU
**KCPMINPF_82206**	Bifunctional ligase/repressor BirA
**KCPMINPF_82291**	Hydrogen peroxide-inducible genes activator
**KCPMINPF_82305**	Transcriptional regulatory protein DegU
**KCPMINPF_82551**	Glc operon transcriptional activator
**KCPMINPF_82718**	Transcriptional regulator SlyA
**KCPMINPF_82734**	Bifunctional transcriptional activator/DNA repair enzyme Ada
**KCPMINPF_82808**	Transcriptional regulatory protein ZraR
**KCPMINPF_82960**	PCP degradation transcriptional activation protein
**KCPMINPF_82993**	N-acetylglucosamine repressor
**KCPMINPF_83038**	Oxygen regulatory protein NreC
**KCPMINPF_83058**	Regulatory protein AtoC
**KCPMINPF_83093**	RNA polymerase-binding transcription factor CarD
**KCPMINPF_83238**	RNA polymerase-binding transcription factor CarD
**KCPMINPF_83360**	Transcriptional repressor NrdR
**KCPMINPF_83376**	Transcriptional regulator LsrR
**KCPMINPF_83467**	Transcriptional regulatory protein LnrK

**KCPMINPF_83504**	Oxygen regulatory protein NreC
**KCPMINPF_83603**	Glucitol operon repressor
**KCPMINPF_83605**	Transcriptional repressor IclR
**KCPMINPF_83633**	Oxygen regulatory protein NreC
**KCPMINPF_83688**	Ribose operon repressor
**KCPMINPF_83758**	Negative regulatory protein YxlE
**KCPMINPF_83772**	Oxygen regulatory protein NreC
**KCPMINPF_83780**	Purine catabolism regulatory protein
**KCPMINPF_83803**	Transcriptional regulatory protein AfsQ1
**KCPMINPF_84033**	Acetoin catabolism regulatory protein
**KCPMINPF_84095**	Methanol dehydrogenase activator
**KCPMINPF_84097**	Transcriptional regulatory protein DegU
**KCPMINPF_84159**	Transcriptional regulatory protein LiaR
**KCPMINPF_84367**	Bifunctional transcriptional activator/DNA repair enzyme Ada
**KCPMINPF_84406**	Transcriptional repressor NrdR
**KCPMINPF_84412**	Murein hydrolase activator EnvC
**KCPMINPF_84683**	Glycerol-3-phosphate regulon repressor
**KCPMINPF_84723**	Heat-inducible transcription repressor HrcA
**KCPMINPF_84759**	Transcriptional regulatory protein WalR
**KCPMINPF_84816**	Transcriptional regulator
**KCPMINPF_84819**	Alginate biosynthesis transcriptional regulatory protein AlgB
**KCPMINPF_84888**	LexA repressor
**KCPMINPF_84889**	Transcriptional regulator LdrP
**KCPMINPF_85012**	Transcriptional activator protein CzcR
**KCPMINPF_85169**	Transcriptional regulatory protein DegU
**KCPMINPF_85179**	Transcriptional regulatory protein WalR
**KCPMINPF_85265**	Transcriptional regulatory protein WalR
**KCPMINPF_85344**	Oxygen regulatory protein NreC
**KCPMINPF_85619**	LexA repressor
**KCPMINPF_85628**	C4-dicarboxylate transport transcriptional regulatory protein DctD
**KCPMINPF_85695**	KDP operon transcriptional regulatory protein KdpE
**KCPMINPF_85737**	Pectin degradation repressor protein KdgR
**KCPMINPF_85851**	KDP operon transcriptional regulatory protein KdpE
**KCPMINPF_85894**	Transcriptional regulatory protein LiaR
**KCPMINPF_85914**	Regulatory protein AtoC
**KCPMINPF_85999**	LexA repressor
**KCPMINPF_86288**	N-acetylglucosamine repressor
**KCPMINPF_86347**	Iron-dependent repressor IdeR
**KCPMINPF_86371**	Bifunctional transcriptional activator/DNA repair enzyme Ada
**KCPMINPF_86422**	Regulatory protein AtoC
**KCPMINPF_86423**	Transcriptional regulatory protein DegU
**KCPMINPF_86900**	Bifunctional ligase/repressor BirA
**KCPMINPF_86924**	Regulatory protein AtoC

**KCPMINPF_86993**	Transcriptional regulatory protein LiaR
**KCPMINPF_87007**	KDP operon transcriptional regulatory protein KdpE
**KCPMINPF_87109**	DNA-binding transcriptional activator DecR
**KCPMINPF_87209**	Regulatory protein AtoC
**KCPMINPF_87218**	Iron-dependent repressor IdeR
**KCPMINPF_87342**	Regulatory protein RecX
**KCPMINPF_87344**	Transcriptional regulator MraZ
**KCPMINPF_87390**	Transcriptional regulatory protein WalR
**KCPMINPF_87497**	Leucine-responsive regulatory protein
**KCPMINPF_87679**	Transcriptional regulator PerR
**KCPMINPF_87762**	Transcriptional regulator PerR
**KCPMINPF_87810**	N-acetylglucosamine repressor
**KCPMINPF_87872**	Transcriptional regulatory protein LiaR
**KCPMINPF_87987**	Heat-inducible transcription repressor HrcA
**KCPMINPF_88056**	Oxygen regulatory protein NreC
**KCPMINPF_88072**	Transcriptional regulatory protein LiaR
**KCPMINPF_88075**	Purine catabolism regulatory protein
**KCPMINPF_88083**	Zinc-specific metallo-regulatory protein
**KCPMINPF_88127**	Oxygen regulatory protein NreC
**KCPMINPF_88140**	RNA polymerase-binding transcription factor DksA
**KCPMINPF_88161**	Alkaline phosphatase synthesis transcriptional regulatory protein PhoP
**KCPMINPF_88313**	Transcriptional regulatory protein DegU
**KCPMINPF_88390**	Transcriptional regulatory protein LiaR
**KCPMINPF_88826**	Transcriptional repressor NrdR
**KCPMINPF_88888**	Alkaline phosphatase synthesis transcriptional regulatory protein PhoP
**KCPMINPF_89084**	Transcriptional regulatory protein WalR
**KCPMINPF_89205**	Iron-dependent repressor IdeR
**KCPMINPF_89298**	Regulatory protein AtoC
**KCPMINPF_89558**	Heat-inducible transcription repressor HrcA
**KCPMINPF_89813**	Transcriptional regulatory protein DegU
**KCPMINPF_89821**	Alkaline phosphatase synthesis transcriptional regulatory protein PhoP
**KCPMINPF_89849**	Oxygen regulatory protein NreC
**KCPMINPF_90092**	Transcriptional regulatory protein QseB
**KCPMINPF_90179**	Transcriptional regulatory protein FixJ
**KCPMINPF_90243**	Transcriptional activator protein CzcR
**KCPMINPF_90307**	Bifunctional transcriptional activator/DNA repair enzyme Ada
**KCPMINPF_90319**	Transcriptional regulatory protein WalR
**KCPMINPF_90368**	Transcriptional regulatory protein WalR
**KCPMINPF_90371**	Acetoin catabolism regulatory protein
**KCPMINPF_90591**	Oxygen regulatory protein NreC
**KCPMINPF_90628**	Transcriptional regulatory protein KdpE
**KCPMINPF_90676**	Transcriptional regulator LsrR
**KCPMINPF_90699**	Transcriptional regulator AcuR

**KCPMINPF_90753**	Transcriptional regulatory protein BaeR
**KCPMINPF_90869**	KDP operon transcriptional regulatory protein KdpE
**KCPMINPF_90920**	Transcriptional regulatory protein QseF
**KCPMINPF_90955**	Transcriptional regulator PerR
**KCPMINPF_90984**	Bifunctional ligase/repressor BirA
**KCPMINPF_91187**	Photosynthetic apparatus regulatory protein RegA
**KCPMINPF_91188**	Trans-acting regulatory protein HvrA
**KCPMINPF_91199**	Transcriptional regulatory protein LiaR
**KCPMINPF_91347**	Oxygen regulatory protein NreC
**KCPMINPF_91438**	Transcriptional activator protein CopR
**KCPMINPF_91487**	Regulatory protein AtoC
**KCPMINPF_91558**	Transcriptional regulatory protein DegU
**KCPMINPF_91653**	Regulatory protein AtoC
**KCPMINPF_91661**	Transcriptional regulatory protein KdpE
**KCPMINPF_91669**	PTS-dependent dihydroxyacetone kinase operon regulatory protein
**KCPMINPF_91756**	Flagellar transcriptional regulator FlhD
**KCPMINPF_91757**	Flagellar transcriptional regulator FlhC
**KCPMINPF_91762**	Transcriptional regulatory protein ZraR
**KCPMINPF_91793**	Photosynthetic apparatus regulatory protein RegA
**KCPMINPF_91811**	Transcriptional regulatory protein LiaR
**KCPMINPF_92189**	Transcriptional regulatory protein AfsQ1
**KCPMINPF_92281**	Transcriptional regulatory protein SrrA
**KCPMINPF_92433**	Glucitol operon repressor
**KCPMINPF_92516**	Hydrogenase transcriptional regulatory protein hupR1
**KCPMINPF_92598**	Transcriptional regulatory protein LiaR
**KCPMINPF_92708**	Glycine cleavage system transcriptional activator
**KCPMINPF_92720**	Transcriptional regulator MraZ
**KCPMINPF_92828**	Transcriptional repressor NrdR
**KCPMINPF_92850**	Transcriptional regulatory protein WalR
**KCPMINPF_92928**	Oxygen regulatory protein NreC
**KCPMINPF_92934**	Transcriptional activator protein CzcR
**KCPMINPF_92949**	Transcriptional regulatory protein WalR
**KCPMINPF_93293**	Transcriptional regulatory protein DegU
**KCPMINPF_93331**	Bifunctional transcriptional activator/DNA repair enzyme Ada
**KCPMINPF_93402**	Transcriptional regulatory protein WalR
**KCPMINPF_93601**	N-acetylglucosamine repressor
**KCPMINPF_93673**	Oxygen regulatory protein NreC
**KCPMINPF_93719**	Regulatory protein AtoC
**KCPMINPF_93960**	Transcriptional regulatory protein LnrK
**KCPMINPF_94036**	Transcriptional regulatory protein DegU
**KCPMINPF_94038**	Oxygen regulatory protein NreC
**KCPMINPF_94345**	Transcriptional regulator MraZ
**KCPMINPF_94808**	Phosphate regulon transcriptional regulatory protein PhoB

**KCPMINPF_94863**	Ribose operon repressor
**KCPMINPF_94987**	Transcriptional regulator KdgR
**KCPMINPF_95120**	Transcriptional repressor SmtB
**KCPMINPF_95214**	Transcriptional regulatory protein RcsB
**KCPMINPF_95233**	Transcriptional regulatory protein LiaR
**KCPMINPF_95384**	Luminescence regulatory protein LuxO
**KCPMINPF_95761**	Redox-sensing transcriptional repressor Rex
**KCPMINPF_95773**	Erythritol catabolism regulatory protein EryD
**KCPMINPF_95856**	Transcriptional regulatory protein TcrA
**KCPMINPF_95869**	Alkaline phosphatase synthesis transcriptional regulatory protein SphR
**KCPMINPF_95889**	Alkaline phosphatase synthesis transcriptional regulatory protein PhoP
**KCPMINPF_95913**	Phosphate regulon transcriptional regulatory protein PhoB
**KCPMINPF_96173**	Transcriptional regulatory protein WalR
**KCPMINPF_96312**	Transcriptional regulatory protein DegU
**KCPMINPF_96363**	Copper-sensing transcriptional repressor CsoR
**KCPMINPF_96483**	Oxygen regulatory protein NreC
**KCPMINPF_96485**	Redox-sensing transcriptional repressor Rex 1
**KCPMINPF_96507**	Oxygen regulatory protein NreC
**KCPMINPF_96531**	Glucitol operon repressor
**KCPMINPF_96631**	DNA-binding transcriptional regulator NtrC
**KCPMINPF_96633**	DNA-binding transcriptional regulator NtrC
**KCPMINPF_97044**	Transcriptional regulatory protein WalR
**KCPMINPF_97111**	Iron-dependent repressor IdeR
**KCPMINPF_97427**	Alkaline phosphatase synthesis transcriptional regulatory protein PhoP
**KCPMINPF_97479**	DNA-binding transcriptional activator DevR/DosR
**KCPMINPF_97607**	Ribose operon repressor
**KCPMINPF_97694**	Alkaline phosphatase synthesis transcriptional regulatory protein PhoP
**KCPMINPF_97699**	Transcriptional regulatory protein WalR
**KCPMINPF_97709**	Ribose operon repressor
**KCPMINPF_97739**	KDP operon transcriptional regulatory protein KdpE
**KCPMINPF_97743**	Transcriptional regulatory protein DegU
**KCPMINPF_97774**	Transcriptional activator protein CzcR
**KCPMINPF_97783**	PTS-dependent dihydroxyacetone kinase operon regulatory protein
**KCPMINPF_97855**	Alkaline phosphatase synthesis transcriptional regulatory protein PhoP
**KCPMINPF_97880**	Transcriptional regulator SlyA
**KCPMINPF_97886**	Copper-sensing transcriptional repressor CsoR
**KCPMINPF_98002**	Transcriptional regulatory protein KdpE
**KCPMINPF_98013**	DNA-binding transcriptional activator DevR/DosR
**KCPMINPF_98024**	N-acetylglucosamine repressor
**KCPMINPF_98107**	Bifunctional ligase/repressor BirA
**KCPMINPF_98151**	Bifunctional ligase/repressor BirA
**KCPMINPF_98319**	Oxygen regulatory protein NreC
**KCPMINPF_98341**	Redox-sensing transcriptional repressor Rex 1

**KCPMINPF_98357**	Transcriptional regulatory protein LiaR
**KCPMINPF_98374**	DNA-binding transcriptional activator DevR/DosR
**KCPMINPF_98432**	Alkaline phosphatase synthesis transcriptional regulatory protein PhoP
**KCPMINPF_98456**	Purine catabolism regulatory protein
**KCPMINPF_98625**	Transcriptional regulatory protein DegU
**KCPMINPF_98642**	Alkaline phosphatase synthesis transcriptional regulatory protein PhoP
**KCPMINPF_98703**	Trp operon repressor
**KCPMINPF_98723**	Alkaline phosphatase synthesis transcriptional regulatory protein PhoP
**KCPMINPF_98935**	Transcriptional repressor IclR
**KCPMINPF_98943**	Transcriptional regulatory protein DegU
**KCPMINPF_98947**	Oxygen regulatory protein NreC
**KCPMINPF_99053**	Transcriptional regulatory protein LiaR
**KCPMINPF_99066**	Alkaline phosphatase synthesis transcriptional regulatory protein PhoP
**KCPMINPF_99198**	Leucine-responsive regulatory protein
**KCPMINPF_99214**	Transcriptional repressor NrdR
**KCPMINPF_99327**	Transcriptional regulator BlaI
**KCPMINPF_99342**	Transcriptional regulatory protein BasR
**KCPMINPF_99353**	Transcriptional regulatory protein DegU
**KCPMINPF_99450**	Oxygen regulatory protein NreC
**KCPMINPF_99576**	Transcriptional regulatory protein WalR
**KCPMINPF_99607**	RNA polymerase-binding transcription factor DksA
**KCPMINPF_99641**	Arabinose metabolism transcriptional repressor
**KCPMINPF_99962**	DnaA regulatory inactivator Hda
**KCPMINPF_99992**	Regulatory protein AtoC
**KCPMINPF_99994**	Transcriptional regulatory protein LiaR
