# Supplementary material for: PredicTF: prediction of bacterial transcription factors in complex microbial communities using deep learning
Source: Environ Microbiome. 2022 Feb 8;17:7. doi: 10.1186/s40793-021-00394-x (PMC8822659; doi:10.1186/s40793-021-00394-x)
Supplement: Supplementary file 7 — Additional file 7: Table S4. The number of Transcription Factors (TFs) per TF family mapped to each of the 11 metatranscriptomes of reference from the same bioreactor where the metagenome (accession number PRJNA511011, NCBI) used to predict the putative TFs was collected. Their European Nucleotide Archive accession numbers represent the different metatranscriptomes. [file 40793_2021_394_MOESM7_ESM.pdf]

PredicTF: prediction of bacterial transcription factors in complex microbial communities using deep learning

Lummy Maria Oliveira Monteiro[1,2,3], Joao Saraiva[1], Rodolfo Brizola Toscan[1], Peter F Stadler[2], Rafael Silva-Rocha[3], Ulisses Nunes da Rocha[1]*

[1] Helmholtz Center for Environmental Research (UFZ), Leipzig, Germany

[2] Universität Leipzig (UL), Leipzig, Germany

[3] Ribeirão Preto Medical School (FMRP), University of São Paulo (USP), Ribeirão Preto, Brazil

*Correspondence: Ulisses Nunes da Rocha, ulisses.rocha@ufz.de

Table S4. The number of Transcription Factors (TFs) per TF family mapped to each of the 11 metatranscriptomes of reference from the same bioreactor where the metagenome (accession number PRJNA511011, NCBI) used to predict the putative TFs was collected. Their European Nucleotide Archive accession numbers represent the different metatranscriptomes.

	**SRR7091381**	**SRR7091385**	**SRR7091400**	**SRR7091401**	**SRR7091402**	**SRR7091406**	**SRR7523233**	**SRR7523243**	**SRR7523244**	**SRR7523245**	**SRR7523246**
	Collection dates (YYYY-MM-DD)[a]										
**TF family**	2016-08-11	2015-08-06	2016-06-21	2016-07-12	2016-08-30	2016-10-27	2015-11-19	2016-11-08	2016-02-12	2016-05-02	2016-11-17
**OmpR/PhoB**	40	18	16	37	29	15	19	2	15	13	25
**LacI/GalR**	31	6	14	19	16	8	9	0	9	9	9
**NarL/FixJ**	26	20	23	37	27	21	20	9	18	18	26
**NtrC/DctD**	17	5	14	21	9	12	8	8	7	12	10
**Fur**	12	5	7	13	7	11	0	3	3	7	10
**LysR**	7	3	8	12	3	3	5	3	7	4	8
**LexA**	7	3	3	8	5	4	4	0	2	2	5
**GntR**	5	2	1	4	2	3	1	1	2	1	1
**CopY**	5	2	3	6	3	1	3	3	3	4	4
**IclR**	4	3	4	5	4	3	4	0	4	3	3
**RelB**	2	0	1	1	0	0	1	0	1	1	1
**MarR/SlyA**	2	1	0	2	2	2	0	0	0	1	2
**Lrp**	2	1	1	3	0	2	1	1	2	2	3
**DtxR/MntR**	2	1	1	2	1	0	1	1	1	1	0
**CsoR**	2	2	2	2	4	3	2	1	1	2	3
**AbiEi**	2	1	1	2	2	2	0	0	1	0	0
**TetR**	1	0	0	0	0	0	0	0	0	0	1
**Rrf2**	1	0	1	3	0	2	0	2	1	0	3
**LuxR**	1	0	0	0	0	0	0	0	0	0	0
**FIS**	1	0	0	1	0	0	0	0	0	0	0
**FNR/CRP**	0	0	0	1	0	0	0	0	0	0	1
**MerR**	0	0	0	3	1	0	0	0	0	0	0
**ArsR**	0	0	0	2	1	0	1	0	1	0	0

[a] Collection dates of the different metatranscriptomes. YYYY, year. MM, month. DD, day.
