# Supplementary material for: PredicTF: prediction of bacterial transcription factors in complex microbial communities using deep learning
Source: Environ Microbiome. 2022 Feb 8;17:7. doi: 10.1186/s40793-021-00394-x (PMC8822659; doi:10.1186/s40793-021-00394-x)
Supplement: Supplementary file 8 — Additional file 8: Table S5. Description of the bacterial transcriptional factors database (BacTFDB) subsets used to train models to predict Transcription Factors in model organisms. [file 40793_2021_394_MOESM8_ESM.pdf]

PredicTF: prediction of bacterial transcription factors in complex microbial communities using deep learning

Lummy Maria Oliveira Monteiro[1,2,3], Joao Saraiva[1], Rodolfo Brizola Toscan[1], Peter F Stadler[2], Rafael Silva-Rocha[3], Ulisses Nunes da Rocha[1]*

[1] Helmholtz Center for Environmental Research (UFZ), Leipzig, Germany

[2] Universität Leipzig (UL), Leipzig, Germany

[3] Ribeirão Preto Medical School (FMRP), University of São Paulo (USP), Ribeirão Preto, Brazil

---

*Correspondence: Ulisses Nunes da Rocha, ulisses.rocha@ufz.de

Table S4. Description of BacTFDB subsets used to train models to predict TFs for model organisms

Organism	Database	Description	Model
Escherichia coli	bacTFDB-no-coli	bacTFDB without *E. coli* transcription factors	PredicTF-no-coli
Bacillus subtilis	bacTFDB-no-subtilis	bacTFDB without *B. subtilis* transcription factors	PredicTF-no-subtilis
Caulobacter crescentus	bacTFDB-no-crescentus	bacTFDB without *C. crescentus* transcription factors	PredicTF-no-crescentus
Pseudomonas fluorescens	bacTFDB-no-fluorescens	bacTFDB without *P. fluorescens* transcription factors	PredicTF-no-fluorescens
Azotobacter vinelandii	bacTFDB-no-vinelandii	bacTFDB without *A. vinelandii* transcription factors	PredicTF-no-vinelandii
