# Supplementary material for: PredicTF: prediction of bacterial transcription factors in complex microbial communities using deep learning
Source: Environ Microbiome. 2022 Feb 8;17:7. doi: 10.1186/s40793-021-00394-x (PMC8822659; doi:10.1186/s40793-021-00394-x)
Supplement: Supplementary file 9 — Additional file 9: Equations. The different equations we used to calculate PredicTF’s accuracy and performance. [file 40793_2021_394_MOESM9_ESM.pdf]

PredicTF: prediction of bacterial transcription factors in complex microbial communities using deep learning

Lummy Maria Oliveira Monteiro[1,2,3], Joao Saraiva[1], Rodolfo Brizola Toscan[1], Peter F Stadler[2], Rafael Silva-Rocha[3], Ulisses Nunes da Rocha[1]*

[1] Helmholtz Center for Environmental Research (UFZ), Leipzig, Germany
[2] Universität Leipzig (UL), Leipzig, Germany
[3] Ribeirão Preto Medical School (FMRP), University of São Paulo (USP), Ribeirão Preto, Brazil

---

*Correspondence: Ulisses Nunes da Rocha, ulisses.rocha@ufz.de

Supplementary Material Online: Equations

The different equations we used to calculate PredicTF's Accuracy and Performance.

Equation 1

$$Performance(\%) = \frac{PredictedTFs * 100}{AnnotatedTFs}$$

where, *Performance (%)* is calculated by the ratio of the total number of TFs predicted by PredicTF (*Predicted TFs*) to the total number of proteins annotated as TFs in NCBI (*Annotated TFs*) multiplied by 100.

Equation 2

$$Accuracy(\%) = \frac{TFspredictedcorrectly * 100}{TFspredicted}$$

where, *Accuracy (%)* is determined by the ratio of the total number of TFs predicted by PredicTF in agreement with NCBI annotation (*TFs predicted correctly*) to the total number of TFs predicted by PredicTF (*TFs predicted*) multiplied by 100.

Equation 3

$$AccuracyforputativeTFs(\%) = \frac{putativeTFspredictedcorrectly * 100}{putativeTFspredicted}$$

where, *Accuracy for putative TFs (%)* is determined by the total number of putative TFs predicted correctly divided by putative TFs predicted multiplied by 100; *Putative TFs predicted correctly* is the total number of putative TFs predicted correctly by PredicTF in agreement with NCBI annotation; and, *Putative TFs predicted* is the total number of putative TFs predicted by PredicTF.
